# Implementation of Recommendations on the Use of Corticosteroids in Severe COVID-19

**DOI:** 10.1001/jamanetworkopen.2023.46502

**Published:** 2023-12-26

**Authors:** Félix Camirand-Lemyre, Laura Merson, Bharath Kumar Tirupakuzhi Vijayaraghavan, Aidan J. C. Burrell, Barbara Wanjiru Citarella, Marie-Pier Domingue, Simon Lévesque, Effua Usuf, Evert-Jan Wils, Shinichiro Ohshimo, Ignacio Martin-Loeches, Oana Sǎndulescu, Jon Henrik Laake, François Lamontagne

**Affiliations:** 1 Université de Sherbrooke, Sherbrooke, Quebec, Canada; 2 ISARIC, Pandemic Sciences Institute, University of Oxford, Oxford, United Kingdom; 3 Department of Critical Care Medicine, Apollo Main Hospital, Chennai, India; 4 The George Institute for Global Health, New Delhi, India; 5 Department of Epidemiology and Preventive Medicine, Australian and New Zealand Intensive Care Research Centre (ANZIC-RC), Monash University, Melbourne, Victoria, Australia; 6 Alfred Hospital, Melbourne, Victoria, Australia; 7 Medical Research Council Unit The Gambia at London School of Hygiene and Tropical Medicine, Fajara, The Gambia; 8 Franciscus Gasthuis & Vlietland, Rotterdam, the Netherlands; 9 Erasmus MC-University Medical Center, Rotterdam, the Netherlands; 10 Department of Emergency and Critical Care Medicine, Graduate School of Biomedical and Health Sciences, Hiroshima University, Hiroshima, Japan; 11 Department of Intensive Care Medicine, Multidisciplinary Intensive Care Research Organization (MICRO), St James’s Hospital Dublin, Leinster, Ireland; 12 Department of Infectious Diseases I, Carol Davila University of Medicine and Pharmacy, Bucharest, Romania; 13 National Institute for Infectious Diseases Prof Dr Matei Balș, Bucharest, Romania; 14 Department of Anaesthesiology and Intensive Care Medicine, Division of Critical Care and Emergencies, Rikshospitalet Medical Centre, Oslo University Hospital, Oslo, Norway

## Abstract

**Importance:**

Research diversity and representativeness are paramount in building trust, generating valid biomedical knowledge, and possibly in implementing clinical guidelines.

**Objectives:**

To compare variations over time and across World Health Organization (WHO) geographic regions of corticosteroid use for treatment of severe COVID-19; secondary objectives were to evaluate the association between the timing of publication of the RECOVERY (Randomised Evaluation of COVID-19 Therapy) trial (June 2020) and the WHO guidelines for corticosteroids (September 2020) and the temporal trends observed in corticosteroid use by region and to describe the geographic distribution of the recruitment in clinical trials that informed the WHO recommendation.

**Design, Setting, and Participants:**

This prospective cohort study of 434 851 patients was conducted between January 31, 2020, and September 2, 2022, in 63 countries worldwide. The data were collected under the auspices of the International Severe Acute Respiratory and Emerging Infections Consortium (ISARIC)–WHO Clinical Characterisation Protocol for Severe Emerging Infections. Analyses were restricted to patients hospitalized for severe COVID-19 (a subset of the ISARIC data set).

**Exposure:**

Corticosteroid use as reported to the ISARIC-WHO Clinical Characterisation Protocol for Severe Emerging Infections.

**Main Outcomes and Measures:**

Number and percentage of patients hospitalized with severe COVID-19 who received corticosteroids by time period and by WHO geographic region.

**Results:**

Among 434 851 patients with confirmed severe or critical COVID-19 for whom receipt of corticosteroids could be ascertained (median [IQR] age, 61.0 [48.0-74.0] years; 53.0% male), 174 307 (40.1%) received corticosteroids during the study period. Of the participants in clinical trials that informed the guideline, 91.6% were recruited from the United Kingdom. In all regions, corticosteroid use for severe COVID-19 increased, but this increase corresponded to the timing of the RECOVERY trial (time-interruption coefficient 1.0 [95% CI, 0.9-1.2]) and WHO guideline (time-interruption coefficient 1.9 [95% CI, 1.7-2.0]) publications only in Europe. At the end of the study period, corticosteroid use for treatment of severe COVID-19 was highest in the Americas (5421 of 6095 [88.9%]; 95% CI, 87.7-90.2) and lowest in Africa (31 588 of 185 191 [17.1%]; 95% CI, 16.8-17.3).

**Conclusions and Relevance:**

The results of this cohort study showed that implementation of the guidelines for use of corticosteroids in the treatment of severe COVID-19 varied geographically. Uptake of corticosteroid treatment was lower in regions with limited clinical trial involvement. Improving research diversity and representativeness may facilitate timely knowledge uptake and guideline implementation.

## Key Points

**Question:**

How did the percentage of patients with severe COVID-19 who received treatment with corticosteroids vary over time (January 2020-September 2022) and across World Health Organization geographic regions?

**Findings:**

In this cohort study of 434 851 patients with severe COVID-19, corticosteroid use increased over time but unequally across geographic regions. The statistical associations between increased corticosteroid use and publication of the Randomised Evaluation of COVID-19 Therapy trial and World Health Organization guidelines were significant only in Europe, where most of the trial recruitment occurred.

**Meaning:**

The findings of this study suggest that a lack of research representativeness may hinder guideline implementation.

## Teaser

This large cohort study assesses variations over time and across World Health Organization geographic regions for the use of corticosteroids in treating patients with severe or critical COVID-19.

## Introduction

The COVID-19 pandemic was undeniably a global crisis. In every country, large numbers of individuals required hospitalization following SARS-CoV-2 infection, and many died.^1^ The response of the international research community was also unprecedented. A surge of studies emerged, many making use of novel designs, rapidly followed by evidence syntheses and, eventually, by clinical practice guidelines created under the auspices of the World Health Organization (WHO). To date, those guidelines include strong recommendations in favor of a limited number of therapeutic interventions for severe (ie, corticosteroids, interleukin 6 receptor blockers, and baricitinib) and nonsevere (nirmatrelvir-ritonavir) COVID-19.^2^

Clinical practice guideline methodology has evolved considerably, leading to the emergence of a series of criteria for “trustworthy” guidelines.^3,4,5,6,7^ However, guideline implementation may vary regardless of the underlying methodological rigor according to intrinsic (ie, related to the guideline) or extrinsic (ie, related to the context in which guidelines are implemented) barriers and facilitators.^8,9^ Given the global scale of the COVID-19 pandemic and the scope of clinical practice guidelines, questions related to the implementation of recommendations by region are particularly relevant.^10^

We undertook an analysis of observational data from the International Severe Acute Respiratory and Emerging Infections Consortium (ISARIC).^11,12,13,14^ The main objective of this cohort study was to compare variations over time and across WHO regions of corticosteroid use for severe COVID-19. The first secondary objective was to evaluate the association between publication of the RECOVERY (Randomised Evaluation of COVID-19 Therapy) trial^15^ and of the WHO guidelines^2^ and the temporal trends in corticosteroid use by region. The second secondary objective was to describe the geographical distribution of the recruitment in clinical trials that informed the WHO guidelines.

## Methods

### Study Design and Participants

This prospective observational cohort study is nested within the ISARIC-WHO Clinical Characterisation Protocol for Severe Emerging Infections.^16,17^ Information on informed patient consent, the case report forms, and the study protocol are available on the ISARIC website.^18^ As of September 2, 2022, investigators from 63 countries used the data collection instruments, which were adapted for a range of resource settings, to prospectively collect data using the ISARIC case report form built using the Research Electronic Data Capture (version 8.11.11, Vanderbilt University) hosted by the University of Oxford (Oxford, England). In addition, some collaborating investigators collected data using locally available systems and submitted data to ISARIC for centralized mapping. All investigators retained full rights to their data. The WHO Ethics Review Committee approved the ISARIC-WHO Clinical Characterisation Protocol. In addition, local ethics approval was obtained for each participating country and site according to local requirements. This report adheres to the Strengthening the Reporting of Observational Studies in Epidemiology (STROBE) reporting guideline for observational studies.

The inclusion criteria were laboratory confirmation of SARS-CoV-2 infection (as defined by the WHO^2,19^) and admission to the hospital for acute illness due to COVID-19. Patients were excluded if they did not have a country code or admission date. We collected information on the following variables defined in the ISARIC data dictionary: age, sex, use of corticosteroids, use of oxygen therapy, admission to the intensive care unit, a selection of comorbidities (hypertension, diabetes, HIV infection, asthma, chronic pulmonary disease, obesity, and tuberculosis), country, country’s income level (lower, lower-middle, and upper-middle vs high income), admission date, and site.

### Operational Definitions of Severe or Critical COVID-19 and of Corticosteroid Use

Cases were categorized as severe or critical COVID-19 if they met any of the following criteria: receipt of oxygen therapy (such as noninvasive positive pressure ventilation, high-flow oxygen nasal cannula, noninvasive ventilation, extracorporeal membrane oxygenation, artificial respiration, oxygen, prone body position, insertion of a tracheostomy tube, removal of an endotracheal tube, or intubation) or admission to an intensive care unit during hospitalization. Corticosteroid use was defined by the administration of any systemic (ie, intravenous or oral) corticosteroid agent at any point during the patients’ COVID-19 hospitalization episodes. The ISARIC database was reviewed for the following terms: *steroids*, *corticosteroids*, *hydrocortisone*, *dexamethasone*, *methylprednisolone*, *prednisolone*, or *prednisone*. Patients who were already treated with corticosteroids for chronic conditions before their hospitalization were not considered as having received corticosteroids for treatment of COVID-19. No restrictions were imposed based on the corticosteroid dose or duration.

### Statistical Analysis

The host repository at the University of Oxford curated data from contributing sites, which used a variety of local data systems, into the Study Data Tabulation Model standards, version 1.7 (Clinical Data Interchange Standards Consortium). The requested data fields for the data extraction period from January 31, 2020, to September 2, 2022, were sent to a secure server of the Study Design and Biostatistics Center at the Université de Sherbrooke, Quebec, Canada. Because the ISARIC collaboration process accommodates sites with a wide range of data collection capabilities, the proportion of complete case reports available for analysis varied substantially across sites and global regions. Our models were based on complete cases (ie, we did not impute missing data). Analyses were conducted using R, version 4.0.3 (R Project for Statistical Computing). To account for multiple testing, we applied Bonferroni corrections to all statistical tests. We reported continuous variables as means (SDs) or medians (IQRs), as appropriate, and categorical variables as counts (percentages).

To compare variations in corticosteroid use for severe COVID-19 across WHO regions (objective 1), we estimated the percentages and corresponding confidence intervals (CIs) of patients with severe COVID-19 who received corticosteroids overall and within WHO geographic regions. Two-sided Wald-type CIs were calculated, with Bonferroni correction applied to maintain a global joint CI of 95%. A χ^2^ test at a significance level of α = 5% was used to test for differences in the percentages of patients who received corticosteroids.

To evaluate the association between publication of the RECOVERY trial^15^ and of the WHO guidelines^2^ and the temporal trends in corticosteroid use by region (objective 2), we used time-varying curve estimates and defined 3 study periods: from the beginning of the COVID-19 pandemic (January 31, 2020) to June 1, 2020 (publication of the RECOVERY trial results for corticosteroids^15^); between June 1 and September 1, 2020 (publication of the WHO guidelines for use of corticosteroids in the treatment of COVID-19^2^); and from September 2, 2020, to September 2, 2022 (end of the study period). We looked for abrupt changes in trends of corticosteroid use over time and across regions using a time-interrupted logistic regression model. The model incorporated 3 variables: corticosteroid use status, time (grouped by month and year), and WHO region (Europe, Africa, Americas, South-East Asia, Eastern Mediterranean, and Western Pacific). Natural cubic spline additive components were used to model smooth time variations that could be attributed to region-specific effects, and time-interruption parameters together with their interactions with the WHO regions variable were added to allow for potential trend interruptions after June 1 and September 1, 2020. To compare region-specific time-interruption parameters, we computed 2-sided CIs for the model coefficients at a global level of 95% using the Wald method and Bonferroni correction. A likelihood ratio test based on the χ^2^ statistic was used at a significance level of α = 5% to determine the significance of any time-interruption parameter. We also compared the percentages of patients who received corticosteroids before June 1 and after September 1, 2020, overall and by WHO geographic region based on CIs. The time-interrupted logistic regression model was based on data collected between March 1, 2020, and May 31, 2022, despite the available data ranging from January 31, 2020, to September 2, 2022, because of insufficient data points in some regions before March 1, 2020, and after May 31, 2022.

To describe the geographic distribution of the recruitment in clinical trials that informed the WHO guidelines (objective 3), we reviewed the published reports of meta-analyses and clinical trials that informed the guidelines. In September 2020, the WHO recommendation in favor of corticosteroid use for patients with severe COVID-19 was informed by a meta-analysis^20^ of 8 randomized clinical trials.^21,22,23,24,25,26,27^ Information regarding the countries in which participants were recruited appears in the appendices of the meta-analysis as well as the primary clinical trials.^2,20,21^ The concurrent recommendation against corticosteroid use in nonsevere COVID-19 was informed entirely by the nonsevere subgroup of the RECOVERY trial,^15^ which enrolled participants in the United Kingdom exclusively. We created heat maps using R packages ggplot2, rgeos, rworldmap, and maps to report the distribution of clinical trial participants alongside the global distribution of ISARIC cases between January 31, 2020, and September 2, 2022.

## Results

The total ISARIC cohort comprised 823 771 patients hospitalized for COVID-19 (mean [SD] age, 55.6 [21.4] years; 50.6% female and 49.4% male). Of these, severity and corticosteroid use could be ascertained for 784 101 patients from 54 countries (eTable 1 in Supplement 1). The age and sex of 39 670 patients who were excluded from this analysis due to missing data regarding COVID-19 severity and corticosteroid use are provided in eTable 2 in Supplement 1.

Of 784 101 patients with ascertainable COVID-19 severity and corticosteroid use, 434 851 had severe or critical COVID-19 (median [IQR] age, 61.0 [48.0-74.0] years; 53.0% male and 46.9% female) (Table 1). Use of invasive mechanical ventilation varied across regions (Table 2).

**Table 1.** General Characteristics of the ISARIC Cohort of Patients With Severe COVID-19 by World Health Organization Geographic Region

Characteristic	Patients, No. (%)						
	Global	Africa	Americas	Eastern Mediterranean	Europe	South-East Asia	Western Pacific
**Demographic characteristic**							
Total	434 851 (100)	221 529 (50.9)	14 167 (3.3)	9345 (2.1)	180 185 (41.4)	8033 (1.8)	1592 (0.4)
Age							
Available	431 806 (99.3)	221 467 (100)	14 142 (99.8)	9345 (100)	177 227 (98.4)	8033 (100)	1592 (100)
Median (IQR), y	61.0 (48.0-74.0)	57.0 (45.0-68.0)	60.0 (47.0-71.0)	60.0 (49.0-70.0)	69.0 (55.0-81.0)	58.0 (46.0-69.0)	59.0 (48.0-68.0)
Sex							
Available	434 851 (100)	221 529 (100)	14 167 (100)	9345 (100)	180 185 (100)	8033 (100)	1592 (100)
Female	203 848 (46.9)	116 167 (52.4)	5493 (38.8)	3434 (36.7)	75 527 (41.9)	2652 (33.0)	575 (36.1)
Male	230 664 (53.0)	105 282 (47.5)	8653 (61.1)	5907 (63.2)	104 429 (58.0)	5380 (67.0)	1013 (63.9)
Unknown	339 (0.1)	80 (0.0)	21 (0.1)	4 (0.0)	229 (0.1)	1 (0.0)	4 (0.3)
**Severity indicator**							
Received oxygen							
Available	434 782 (100)	221 529 (100)	14 167 (100)	9345 (100)	180 118 (100)	8033 (100)	1590 (99.9)
Yes	404 942 (93.1)	199 266 (90.0)	13 354 (94.3)	9317 (99.7)	173 574 (96.4)	7935 (98.8)	1496 (94.1)
Treated in ICU							
Available	432 095 (99.4)	221 528 (100)	14 157 (99.9)	9345 (100)	177 440 (98.5)	8033 (100)	1592 (100)
Yes	164 160 (38.0)	86 842 (39.2)	9631 (68.0)	9343 (100)	49 661 (28.0)	7626 (94.9)	1057 (66.4)
**Comorbidity**							
Hypertension							
Available	350 101 (80.5)	174 692 (78.9)	13 696 (96.7)	9313 (99.7)	143 552 (79.7)	7705 (95.9)	1143 (71.8)
Yes	156 204 (44.6)	76 207 (43.6)	7065 (51.6)	4423 (47.5)	64 914 (45.2)	3034 (39.4)	561 (49.1)
Diabetes							
Available	360 380 (82.9)	166 526 (75.2)	13 936 (98.4)	9238 (98.9)	161 411 (89.6)	7696 (95.8)	1573 (98.8)
Yes	101 888 (28.3)	45 657 (27.4)	7793 (55.9)	2103 (22.8)	44 309 (27.5)	1526 (19.8)	500 (31.8)
HIV infection							
Available	342 066 (78.7)	157 790 (71.2)	11 648 (82.2)	8522 (91.2)	156 515 (86.9)	6277 (78.1)	1314 (82.5)
Yes	14 225 (4.2)	13 475 (8.5)	119 (1.0)	2 (0.0)	603 (0.4)	21 (0.3)	5 (0.4)
Asthma							
Available	351 830 (80.9)	158 263 (71.4)	13 861 (97.8)	9327 (99.8)	161 090 (89.4)	7707 (95.9)	1582 (99.4)
Yes	31 782 (9.0)	7624 (4.8)	1294 (9.3)	409 (4.4)	22 199 (13.8)	129 (1.7)	127 (8.0)
Chronic pulmonary disease							
Available	350 751 (80.7)	154 864 (69.9)	13 876 (97.9)	9326 (99.8)	163 390 (90.7)	7713 (96.0)	1582 (99.4)
Yes	33 413 (9.5)	3969 (2.6)	1291 (9.3)	159 (1.7)	27 584 (16.9)	338 (4.4)	72 (4.6)
Obesity							
Available	227 973 (52.4)	54 513 (24.6)	8491 (59.9)	9299 (99.5)	146 818 (81.5)	7322 (91.1)	1530 (96.1)
Yes	42 506 (18.6)	11 957 (21.9)	2559 (30.1)	171 (1.8)	27 393 (18.7)	193 (2.6)	233 (15.2)
Tuberculosis							
Available	201 270 (46.3)	156 180 (70.5)	10 209 (72.1)	9314 (99.7)	17 253 (9.6)	7697 (95.8)	617 (38.8)
Yes	4229 (2.1)	3981 (2.5)	72 (0.7)	27 (0.3)	78 (0.5)	64 (0.8)	7 (1.1)

Abbreviations: ICU, intensive care unit; ISARIC, International Severe Acute Respiratory and Emerging Infections Consortium.

**Table 2.** Characteristics of Participating Sites by World Health Organization Geographic Region

Characteristic	Sites, No. (%)						
	Overall	Africa^a^	Americas	Eastern Mediterranean	Europe	South-East Asia	Western Pacific
Distribution of contributing sites	1437 of 1437 (100)	642 of 1437 (44.7)	174 of 1437 (12.1)	70 of 1437 (4.9)	467 of 1437 (32.5)	41 of 1437 (2.9)	44 of 1437 (3.1)
In a country with low, lower-middle, or upper-middle income	789 of 1437 (54.9)	642 of 642 (100)	23 of 174 (13.2)	63 of 70 (90.0)	6 of 467 (1.3)	41 of 41 (100)	14 of 44 (31.8)
In a region with ≥80% of severe COVID-19 cases treated in the ICU	384 of 806 (47.6)	7 of 11 (63.6)	93 of 174 (53.4%)	70 of 70 (100)	140 of 467 (30.0)	36 of 41 (87.8)	38 of 44 (86.4)
In a region with at least 1 severe or critical COVID-19 case treated with invasive mechanical ventilation	699 of 806 (86.7)	9 of 11 (81.8)	168 of 174 (96.6)	70 of 70 (100)	372 of 467 (79.6)	40 of 41 (97.6)	40 of 44 (90.9)

Abbreviation: ICU, intensive care unit.

^a^ Patients from South Africa were treated across 631 hospitals. Although information was obtained regarding the treatments and outcomes of individual patients, the distribution of patients across South African sites, as well as site-level characteristics, are missing. Accordingly, 631 sites from South Africa were removed from these proportions.

### Variations in Corticosteroid Use

**Figure 1.**
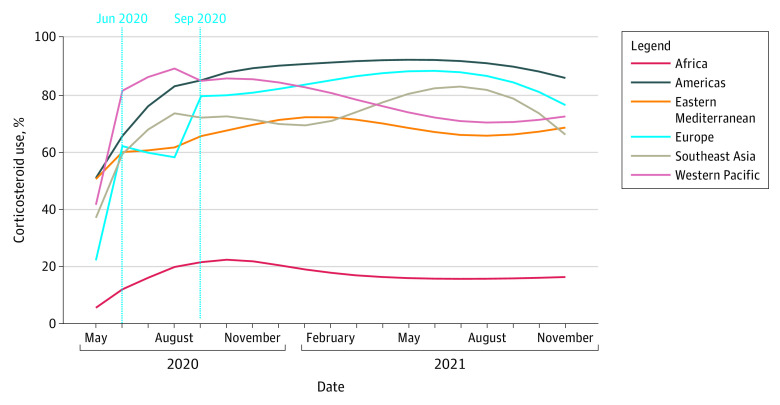
Corticosteroid Use Among Severe Cases of COVID-19 in the International Severe Acute Respiratory and Emerging Infections Consortium (ISARIC) Cohort

Among 434 851 patients with confirmed severe or critical COVID-19 for whom receipt of corticosteroids could be ascertained, 174 307 (40.1%) received corticosteroids during the study period. In all regions, corticosteroid use for treatment of severe cases increased over time (Table 3; Figure 1). Globally, the number of severe or critical patients in the ISARIC data set who received corticosteroids was 16 312 of 63 017 (25.9%; Bonferroni-corrected 95% CI, 25.3%-26.4%) from January 31 to May 31, 2020, and increased to 146 204 of 328 070 (44.6%; Bonferroni-corrected 95% CI, 44.3%-44.8%) from September 1, 2020, to September 1, 2022. At the end of the study period, corticosteroid use varied across regions (likelihood ratio test: *P* < .001), with the highest percentage in the Americas (5421 of 6095; 88.9% [Bonferroni-corrected 95% CI, 87.7%-90.2%]) and the lowest percentage in Africa (31 588 of 185 191; 17.1% [Bonferroni-corrected 95% CI, 16.8%-17.3%]).

**Table 3.** Receipt of Corticosteroids Among Patients in ISARIC Data Set With Severe or Critical COVID-19 Disease, by Time Period and World Health Organization Geographic Region

Period	Patients, No. (%) [Bonferroni-corrected 95% CI]						
	Global	Africa	Americas	Eastern Mediterranean	Europe	South-East Asia	Western Pacific
January 31 to May 31, 2020	16 312 of 63 017 (25.9) [25.3-26.4]	119 of 2363 (5.0) [3.7-6.4]	2098 of 5970 (35.1) [33.2-37.0]	501 of 999 (50.2) [45.3-55.0]	13 321 of 52 650 (25.3) [24.7-25.9]	67 of 242 (27.7) [18.8-36.5]	206 of 793 (26.0) [21.2-30.8]
June 1 to August 31, 2020	11 791 of 43 764 (26.9) [26.3-27.6]	5495 of 33 975 (16.2) [15.6-16.8]	1528 of 2102 (72.7) [69.7-75.7]	1149 of 1912 (60.1) [56.6-63.5]	2675 of 4458 (60.0) [57.7-62.3]	735 of 1076 (68.3) [63.9-72.7]	209 of 241 (86.7) [80.0-93.5]
September 1, 2020, to September 1, 2022	146 204 of 328 070 (44.6) [44.3-44.8]	31 588 of 185 191 (17.1) [16.8-17.3]	5421 of 6095 (88.9) [87.7-90.2]	4447 of 6434 (69.1) [67.3-70.9]	99 434 of 123 077 (80.8) [80.4-81.1]	4895 of 6715 (72.9) [71.2-74.6]	419 of 558 (75.1) [69.5-80.7]

Abbreviation: ISARIC, International Severe Acute Respiratory and Emerging Infections Consortium.

Corticosteroid use increased abruptly and significantly in Europe both in June 2020 (time-interruption coefficient for the uptake, 1.0 [Bonferroni-corrected 95% CI, 0.9-1.2]) and September 2020 (time-interruption coefficient for uptake, 1.9 [95% Bonferroni-corrected CI, 1.7-2.0]). The increase was not associated with those dates in the other regions (ie, all other Bonferroni-corrected 95% CIs for the associated time-interruption coefficients contained the value 0 and were, therefore, not statistically significant).

### Geographic Distribution of Trial Participants vs ISARIC Cases

**Figure 2.**
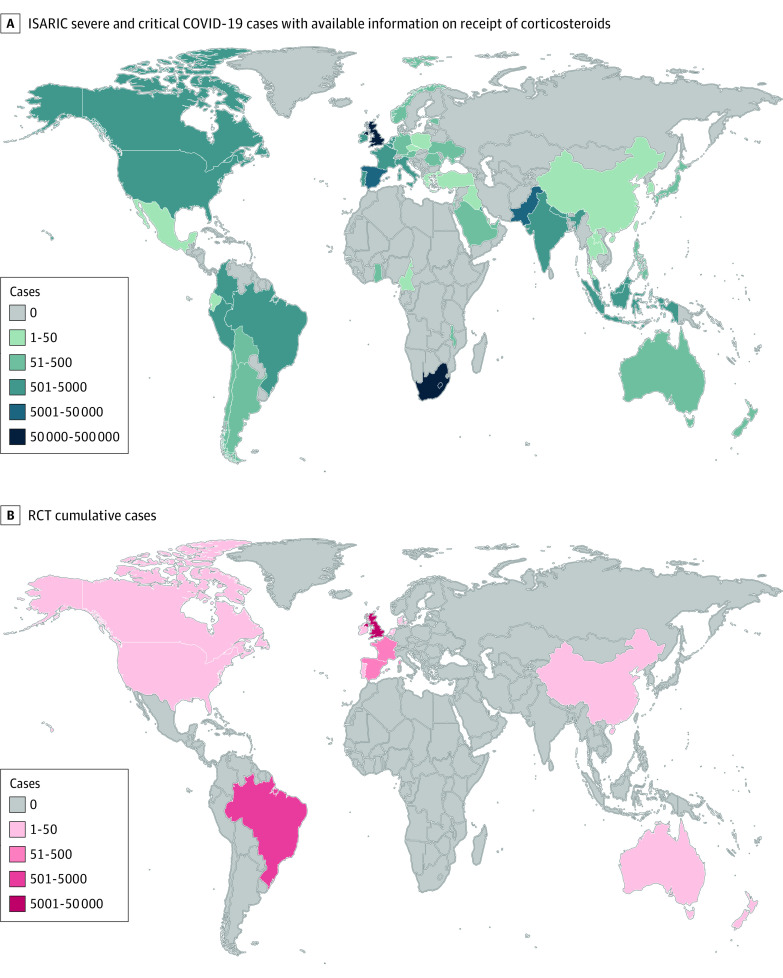
Global Distribution of Severe COVID-19 Cases in the International Severe Acute Respiratory and Emerging Infections Consortium (ISARIC) Database and in the Clinical Trials That Informed World Health Organization Guidelines for Corticosteroid Use in COVID-19 RCT indicates randomized clinical trial.

The WHO recommendation to administer corticosteroids to patients with severe and critical COVID-19 hinged on data from 8 clinical trials^21,22,23,24,25,26,27^ that enrolled a total of 7184 participants (including 1 trial^28^ that remained unpublished as of publication of the present study). Of the trial participants included, 91.6% were recruited from the United Kingdom. Figure 2 illustrates the geographic distribution of patients hospitalized for COVID-19 who were included in the ISARIC database and who were recruited in clinical trials of corticosteroids.

## Discussion

The results of this cohort study including 434 851 patients hospitalized for severe COVID-19 across 54 countries from all regions of the globe suggest that while corticosteroid use increased in all regions, important geographic variations persisted until the end of the study period, September 2022. Moreover, the timing of changes observed in corticosteroid use coincided with publication of the RECOVERY trial^15^ and of the WHO guidelines^2^ for corticosteroids, most evidently in Europe, which is also where the clinical trials recruited a majority of the research participants.

Although many other factors influence guideline implementation, these results underscore the need to address the issue of global clinical research representativeness. Overwhelmingly, clinical trial data are derived mainly from a limited number of high-income countries, even when these countries bear a relatively small portion of the global burden of a disease (eg, sepsis^29^). In comparison with many other therapies, corticosteroids are widely regarded as safe, available, and inexpensive. In this context, it is noteworthy that this study found that uptake of the WHO recommendation was more modest and slower in regions that were underrepresented in the clinical trials that informed the guidelines. While it is unclear to what extent a lack of trust in the regional applicability of the underlying trial evidence explains the heterogeneous guideline implementation, it is widely accepted that diversity and representativeness in research is paramount in “building trust, promoting fairness, and generating biomedical knowledge.”^30^ Ultimately, it may prove an uphill battle to argue for global implementation of guidelines informed by studies that recruited research participants in a small number of mostly high-income countries. And if concerns regarding the limited applicability of research data played a role in the uptake of corticosteroids for treatment of COVID-19, it is plausible that such concerns would be of an order of magnitude greater for interventions that are costlier, less widely available, and riskier. Therefore, while the pandemic spurred an unprecedented collaboration between the research and guideline communities, it should also serve as a reckoning that high-quality health care hinges on research data that accurately reflect the target populations and environments in which interventions will be delivered. Potential ways to enhance clinical research capacity are discussed elsewhere^31,32^ and are beyond the scope of this work. From the perspective of scientific journals and guidelines, there exists an opportunity to raise awareness regarding issues of poor trial representativeness. The GRADE (Grading of Recommendations, Assessment, Development, and Evaluations) framework already provides a structure to evaluate the extent to which research findings apply to the population targeted by a guideline (ie, directness).^33^ A more systematic appraisal of trial representativeness by journal editorial boards and guideline panels, as well as potential repercussions on the likelihood of publication and the strength and direction of ensuing recommendations, may provide strong incentives for trialists and research networks to invest in the diversification of research populations. Meanwhile, the real-time monitoring of the global clinical research portfolio—including its gaps and redundancies—in parallel with an accurate depiction of health interventions delivered around the world (ie, guideline implementation) is an unmet need.

### Limitations

This work was derived from the largest and most representative data set of hospitalized patients with COVID-19, spanning all surges of the COVID-19 pandemic. Notwithstanding, we acknowledge the following study limitations. Although the ISARIC data set provides information on all WHO regions and is more globally representative than the clinical trials that informed WHO recommendations, not all regions were equally represented, and regional representation varied over time. In some cases, a single country eclipsed all others within their region (eg, South Africa contributed 99.4% of the records included from Africa). In such instances, inferences may not be applicable to the entire region. In the absence of a reliable global registry of hospitalization for COVID-19, we were unable to evaluate the representativeness of the ISARIC data set. Similar information regarding corticosteroid use may be available on the WHO COVID-19 dashboard.^34^ However, the WHO data are not available for external access, and the ISARIC data set makes up in data quality and depth for what it lacks in comprehensiveness (enabling estimates of data missingness). Moreover, considering that the corticosteroid guidelines were created under the auspices of the WHO, the use of the ISARIC data set may have increased the risk of reporting bias from member states potentially tempted to report high adherence to WHO recommendations when reporting to the WHO. Guideline implementation is potentially influenced by many barriers and facilitators beyond clinical trial representativeness. While it was beyond the scope of this analysis to fully explain the global variability in corticosteroid use, implementation science and operational research could further shed light on this issue in support of improved guideline implementation and monitoring strategies. Having focused exclusively on corticosteroids, this analysis may overestimate the uptake of WHO recommendations for severe COVID-19 given the lesser availability and higher costs of other recommended therapies, such as interleukin 6 receptor blockers and baricitinib.

## Conclusions

This cohort study found that corticosteroid use for treatment of severe and critical COVID-19 increased over time in all WHO geographic regions. However, it remained low in Africa compared with Europe, where clinical trials assessing corticosteroids for treatment of COVID-19 enrolled the most participants. The findings of this study suggest that encouraging research diversity and representativeness may facilitate timely knowledge uptake and guideline implementation.

## Notes

Supplement 1. **eTable 1.** Countries Represented in the ISARIC Cohort Study Conducted Between January 31st 2020 and September 2nd 2022 **eTable 2.** Age and Sex of Cases With Missing Data for Illness Severity and/or Corticosteroid Use (Excluded From This Analysis)

Supplement 2.
Nonauthor Collaborators

Supplement 3.
Data Sharing Statement
