# Supplementary material for: Implementation of Recommendations on the Use of Corticosteroids in Severe COVID-19
Source: JAMA Netw Open. 2023 Dec 26;6(12):e2346502. doi: 10.1001/jamanetworkopen.2023.46502 (PMC10751594; doi:10.1001/jamanetworkopen.2023.46502)
Supplement: Supplement 1. — eTable 1. Countries Represented in the ISARIC Cohort Study Conducted Between January 31st 2020 and September 2nd 2022 eTable 2. Age and Sex of Cases With Missing Data for Illness Severity and/or Corticosteroid Use (Excluded From This Analysis) [file jamanetwopen-e2346502-s001.pdf]

Supplementary Online Content

Camirand-Lemyre F, Merson L, Tirupakuzhi Vijayaraghavan BK, et al; ISARIC Clinical Characterisation Group. Implementation of recommendations on the use of corticosteroids in severe COVID-19. *JAMA Netw Open*. 2023;6(12):e2346502. doi:10.1001/jamanetworkopen.2023.46502

eTable 1. Countries Represented in the ISARIC Cohort Study Conducted Between January 31st 2020 and September 2nd 2022
eTable 2. Age and Sex of Cases With Missing Data for Illness Severity and/or Corticosteroid Use (Excluded From This Analysis)

This supplementary material has been provided by the authors to give readers additional information about their work.

© 2023 Camirand-Lemyre F et al. *JAMA Network Open*.

eTable 1. Countries represented in the ISARIC cohort study conducted between January 31st 2020 and September 2nd 2022

Country	Number of ISARIC cases	Number of ISARIC COVID-19 cases	Number of cases with available information on illness severity and corticosteroid use	Number of cases included in this analysis
Argentina	165	140	137	137
Australia	695	419	417	412
Austria	68	56	56	56
Bangladesh	3	0	0	0
Belgium	1341	857	824	705
Bolivia	142	142	141	140
Brazil	5431	4471	3962	3540
Cameroon	1560	619	254	3
Canada	6004	5731	5417	4168
Chile	138	113	111	110
China	12	12	12	12
Colombia	966	745	602	591
Croatia	8	0	0	0
Czechia	21	18	18	18
Dominican Republic	13	0	0	0
Ecuador	31	9	9	5
Estonia	155	139	63	63
France	5271	5101	3584	2766
Gambia	187	71	71	71
Germany	192	185	184	145
Ghana	52	51	51	51
Gibraltar	476	441	0	0
Greece	41	39	36	21
Hong Kong SAR China	20	19	19	19
India	9170	8576	4313	3649
Indonesia	1314	1106	1089	912
Iraq	2	2	2	2
Ireland	1956	1467	1274	895
Israel	2342	2173	404	119
Italy	6622	4029	3802	2788
Japan	245	101	98	91
Kenya	1	0	0	0
Kuwait	821	801	744	744
Laos	2677	500	32	13

© 2023 Camirand-Lemyre F et al. *JAMA Network Open*.

Luxembourg	735	735	0	0
Malawi	1072	324	310	210
Malaysia	6680	6543	6447	724
Mexico	24	22	22	22
Nepal	3469	3469	3469	3469
Netherlands	2780	2188	2088	1861
New Zealand	146	127	119	114
Norway	7532	4178	594	458
Pakistan	8266	8266	8266	8266
Peru	1675	1653	1563	1275
Philippines	489	363	352	190
Poland	542	117	29	11
Portugal	1986	1374	1052	1005
Qatar	154	57	2	2
Romania	1287	1090	1082	207
Russia	4100	2104	0	0
Saudi Arabia	343	293	277	275
Singapore	1	0	0	0
South Africa	485440	465738	465729	221194
South Korea	44	17	16	16
Spain	17805	15262	15190	13454
Sudan	7	4	0	0
Taiwan	1	1	1	1
Thailand	8	8	8	3
Turkey	57	51	51	49
Ukraine	212	212	211	210
United Arab Emirates	62	58	56	56
United Kingdom	294278	266547	244761	155354
United States	5737	4837	4680	4179
**Total**	**893074**	**823771**	**784101**	**434851**

© 2023 Camirand-Lemyre F et al. *JAMA Network Open*.

eTable 2. Age and sex of cases with missing data for illness severity and/or corticosteroid use (excluded from this analysis)

n=39670		
Age; mean, standard deviation	56.6	20.0
Female sex; n, %	18293	46.1

© 2023 Camirand-Lemyre F et al. *JAMA Network Open*.
