# Supplementary material for: Implementation of Recommendations on the Use of Corticosteroids in Severe COVID-19
Source: JAMA Netw Open. 2023 Dec 26;6(12):e2346502. doi: 10.1001/jamanetworkopen.2023.46502 (PMC10751594; doi:10.1001/jamanetworkopen.2023.46502)
Supplement: Supplement 2. — Nonauthor Collaborators [file jamanetwopen-e2346502-s002.pdf]

*Group Name(s): ISARIC Clinical Characterisation Group							
***First Name and Middle Initial(s)**	***Last Name**	***Suffix (eg, Jr, III)**	Academic Degrees	Institution	Location (city, state/province, country)	Role or Contribution, eg, chair, principal investigator	Group (if more than 1 Group listed in the byline) and/or Subgroup (eg, Steering Committee)
Sheryl Ann	Abdukahil			King Abdulaziz Medical City, Riyadh, Saudi Arabia	Saudi Arabia		
Nurul Najmee	Abdulkadir			Tuanku Fauziah Hospital, Perlis, Malaysia	Malaysia		
Ryuzo	Abe			Chiba University Hospital, Chiba, Japan	Japan		
Laurent	Abel			INSERM, Paris, France	France		
Amal	Abrous			INSERM, Paris, France	France		
Lara	Absil			CUB-Hopital Erasme, Anderlecht, Belgium	Belgium		
Kamal	Abu Jabal			Ziv Medical Centre Safed, Israel	Israel		
Nashat	Abu Salah			Ziv Medical Centre Safed, Israel	Israel		
Andrew	Acker			Perelman School of Medicine at the University of Pennsylvania, Philadelphia, United States of America	United States of America		
Elisabeth	Adam			Uniklinik University Hospital, Frankfurt, Germany	Germany		
Diana	Adrião			Centro Hospitalar Vila Nova de Gaia/Espinho, Espinho, Portugal	Portugal		
Saleh Al	Ageel			King Faisal Hospital Research Center, Riyadh, Saudi Arabia	Saudi Arabia		
Shakeel	Ahmed			University Hospital, Kerry, Ireland	Ireland		
Kate	Ainscough			St Vincents University Hospital, Dublin, Ireland	Ireland		
Eka	Airlangga			Murni Teguh Memorial Hospital and Bunda Thamrin Hospital, North Sumatera, Indonesia	Indonesia		
Tharwat	Aisa			Our lady of Lourdes Drogheda, Drogheda, Ireland	Ireland		
Ali	Ait Hssain			Hamad General Hospital, Doha, Qatar	Qatar		
Younes	Ait Tamlihat			Centre Hospitalier de Saintonge, Saintes, France	France		
Takako	Akimoto			Teine Keijinkai Hospital, Sapporo, Japan	Japan		
Ernita	Akmal			Persahabatan Hospital, Jakarta, Indonesia	Indonesia		
Chika	Akwani			University of Nebraska Medical Centre	United States of America		

*First Name and Middle Initial(s)	*Last Name	*Suffix (eg, Jr, III)	Academic Degrees	Institution	Location (city, state/province, country)	Role or Contribution, eg, chair, principal investigator	Group (if more than 1 Group listed in the byline) and/or Subgroup (eg, Steering Committee)
Eman	Al Qasim			King Abdulaziz Medical City, Riyadh, Saudi Arabia	Saudi Arabia		
Razi	Alalqam			Beaumont Hospital, Dublin, Ireland	Ireland		
Aliya Mohammed	Alameen			Al-Neelain university, Khartoum, Sudan	Sudan		
Angela	Alberti			Northwell Health, New York, United States of America	United States of America		
Tala	Al-dabbous			Al-Adan Hospital, Hadiya, Kuwait	Kuwait		
Senthilkumar	Alegesan			Galway University Hospital, Galway, Ireland	Ireland		
Marta	Alessi			University Hospital Policlinico Paolo Giaccone, Palermo, Italy	Italy		
Beatrice	Alex			School of Informatics, University of Edinburgh, Edinburgh, UK	United Kingdom		
Kévin	Alexandre			Centre Hospitalier Universitaire Rouen (Center Hospitalier Universitaire de Rouen), Rouen, France	France		
Abdulrahman	Al-Fares			Al-Amiri & Jaber Al-Ahmed Hospitals, Kuwait City, Kuwait	Kuwait		
Huda	Alfoudri			Al-Adan Hospital, Hadiya, Kuwait	Kuwait		
Imran	Ali			Sligo University Hospital (Saolta), Sligo, Ireland	Ireland		
Adam	Ali			St Bernard's Hospital, Gibraltar, Gibraltar	Gibraltar		
Kazali Enagnon	Alidjnou			Centre Hospitalier Universitaire de Lille, Lille, France	France		
Qabas	Alkhafajee			University Hospital, Kerry, Ireland	Ireland		
Clotilde	Allavena			Centre Hospitalier Universitaire de Nantes (Hôpital femme-enfant-adolescent), Nantes, France	France		
Nathalie	Allou			Centre Hospitalier Félix-Guyon, Saint-Denis, Réunion	Réunion		
João	Alves			Centro Hospital e Universitário de Coimbra, Coimbra, Portugal	Portugal		
Rita	Alves			Hospital Curry Cabral - Intensive Care Unit - UCIP7, Lisbon, Portugal	Portugal		

*First Name and Middle Initial(s)	*Last Name	*Suffix (eg, Jr, III)	Academic Degrees	Institution	Location (city, state/province, country)	Role or Contribution, eg, chair, principal investigator	Group (if more than 1 Group listed in the byline) and/or Subgroup (eg, Steering Committee)
João Melo	Alves			Hospital de São José -U.U.M., Lisbon, Portugal	Portugal		
Joana	Alves Cabrita			Hospital Curry Cabral - Intensive Care Unit - UCIP7, Lisbon, Portugal	Portugal		
Maria	Amaral			Hospital Curry Cabral - Intensive Care Unit - UCIP7, Lisbon, Portugal	Portugal		
Nur	Amira			Sungai Buloh Hospital, Selangor, Malaysia	Malaysia		
Roberto	Andini			University of Campania, Carseta, Italy	Italy		
Claire	Andréjak			Centre Hospitalier Universitaire Amiens-Picardie, Amiens, France	France		
Andrea	Angheben			Department of Infectious - Tropical Diseases and Microbiology - IRCCS Sacro Cuore Hospital - Negrar - Italy	Italy		
François	Angoulvant			INSERM, Paris, France	France		
Séverine	Ansart			Centre Hospitalier Universitaire de Brest, Brest, France	France		
Sivanesen	Anthonidass			Kuala Lumpur Hospital, WPKL, Malaysia	Malaysia		
Massimo	Antonelli			Fondazione Policlinico Universitario Agostino Gemelli IRCCS, Rome, Italy	Italy		
Carlos Alexandre	Antunes de Brito			Centro de Pesquisa Aggeu Magalhães, Fiocruz, Recife, Brazil	Brazil		
Ardiyan	Apriyana			National Cardiovascular Center Harapan Kita Jakarta Indonesia, Jakarta, Indonesia	Indonesia		
Yaseen	Arabi			Intensive Care Department, Ministry of National Guard Health Affairs, Riyadh, Saudi Arabia	Saudi Arabia		
Irene	Aragao			Centro Hospitalar Universitário do Porto (CHUP), Porto, Portugal	Portugal		
Francisco	Arancibia			Instituto Nacional Del Tórax, Santiago, Chile	Chile		
Carolline	Araujo			Centro de Pesquisa Aggeu Magalhães, Fiocruz, Recife, Brazil	Brazil		
Antonio	Arcadipane			Istituto Mediterraneo per i Trapianti e Terapie ad Alta Specializzazione, Palermo, Italy	Italy		

*First Name and Middle Initial(s)	*Last Name	*Suffix (eg, Jr, III)	Academic Degrees	Institution	Location (city, state/province, country)	Role or Contribution, eg, chair, principal investigator	Group (if more than 1 Group listed in the byline) and/or Subgroup (eg, Steering Committee)
Patrick	Archambault			CISSS Chaudière-Appalaches, Canada	Canada		
Lukas	Arenz			LMU Hospital Munich, Medical Department II, Campus Großhadern, Munich, Germany	Germany		
Jean-Benoît	Arlet			Hôpital Européen Georges-Pompidou AP-HP, Paris, France	France		
Christel	Arnold-Day			Groote Schuur Hospital, Cape Town, South Africa	South Africa		
Lovkesh	Arora			University of Iowa, Iowa City, United States of America	United States of America		
Rakesh	Arora			St. Boniface Hospital, Manitoba, Canada	Canada		
Elise	Artaud-Macari			Centre Hospitalier Universitaire Rouen (Center Hospitalier Universitaire de Rouen), Rouen, France	France		
Angel	Asensio			Hospital Puerta de Hierro Majadahonda, Madrid, Spain	Spain		
Elizabeth A.	Ashley			Lao-Oxford-Mahosot Hospital-Wellcome Trust Research Unit, Vientiane, Laos	Laos		
Muhammad	Ashraf			Sungai Buloh Hospital, Selangor, Malaysia	Malaysia		
Jean Baptiste	Assie			Centre Hospitalier intercommunal de Créteil, Créteil, France	France		
Amirul	Asyraf			Sungai Buloh Hospital, Selangor, Malaysia	Malaysia		
Minahel	Atif			Galway University Hospital, Galway, Ireland	Ireland		
Anika	Atique			McGill University Health Centre, Montreal, Canada	Canada		
AM Udara Lakshan	Attanyake			CCA Network	Unknown		
Johann	Auchabie			Centre Hospitalier de Cholet, Cholet, France	France		
Hugues	Aumaitre			Centre Hospitalier de Perpignan, Perpignan, France	France		
Adrien	Auvet			Hôpital Européen Georges-Pompidou AP-HP, Paris, France	France		
Eyvind W.	Axelsen			The Norwegian Corona Cohort, Oslo, Norway	Norway		
Laurène	Azemar			Hôpital Lariboisière AP-HP, Paris, France	France		

*First Name and Middle Initial(s)	*Last Name	*Suffix (eg, Jr, III)	Academic Degrees	Institution	Location (city, state/province, country)	Role or Contribution, eg, chair, principal investigator	Group (if more than 1 Group listed in the byline) and/or Subgroup (eg, Steering Committee)
Cecile	Azoulay			Hôpital Cochin AP-HP, Paris, France	France		
Hakeem	Babatunde			Clinical Services Department Fajara, Gambia	Gambia		
Benjamin	Bach			School of Informatics, University of Edinburgh, Edinburgh, UK	United Kingdom		
Delphine	Bachelet			INSERM, Paris, France	France		
Claudine	Badr			Centre Hospitalier Intercommunal Villeneuve-Saint-Georges, Villeneuve-Saint-Georges, France	France		
Roar	Bævre-Jensen			The Norwegian Corona Cohort, Oslo, Norway	Norway		
Nadia	Baig			Grande Prairie Queen Elizabeth II, Grande Prairie, Canada	Canada		
John Kenneth	Baillie			Roslin Institute, University of Edinburgh, Edinburgh, UK	United Kingdom		
J Kevin	Baird			Murni Teguh Memorial Hospital and Bunda Thamrin Hospital, North Sumatera, Indonesia	Indonesia		
Erica	Bak			Rush University Medical Center, Chicago, United States of America	United States of America		
Agamemnon	Bakakos			Sotiria General Hospital, Athens, Greece	Greece		
Nazreen Abu	Bakar			Tuanku Fauziah Hospital, Perlis, Malaysia	Malaysia		
Andriy	Bal			Unidade Local de Saúde de Alto Minho, Viana Do Castelo, Portugal	Portugal		
Mohanaprasanth	Balakrishnan			Sungai Buloh Hospital, Selangor, Malaysia	Malaysia		
Firouzé	Bani-Sadr			Centre Hospitalier Universitaire de Reims, Reims, France	France		
Renata	Barbalho			Instituto de Infectologia Emílio Ribas, Sao Paulo, Brazil	Brazil		
Nicholas Yuri	Barbosa			Caja Nacional De Salud, Trinidad, Bolivia	Bolivia		
Wendy S.	Barclay			Section of Molecular Virology, Imperial College London, London, UK	United Kingdom		
Saef Umar	Barnett			Sungai Buloh Hospital, Selangor, Malaysia	Malaysia		
Michaela	Barnikel			LMU Hospital Munich, Medical Department II, Campus Großhadern, Munich, Germany	Germany		
Helena	Barrasa			Hospital Universitario de Alava, Araba, Spain	Spain		

*First Name and Middle Initial(s)	*Last Name	*Suffix (eg, Jr, III)	Academic Degrees	Institution	Location (city, state/province, country)	Role or Contribution, eg, chair, principal investigator	Group (if more than 1 Group listed in the byline) and/or Subgroup (eg, Steering Committee)
Cleide	Barrigoto			Hospital de São José -U.U.M., Lisbon, Portugal	Portugal		
Marie	Bartoli			INSERM, Paris, France	France		
Joaquín	Baruch			ISARIC, Pandemic Sciences Institute, University of Oxford, Oxford, UK	United Kingdom		
Romain	Basmaci			INSERM, Paris, France	France		
Muhammad Fadhli Hassin	Basri			Sungai Buloh Hospital, Selangor, Malaysia	Malaysia		
Denise	Battaglini			San Martino Hospital, Genoa, Italy	Italy		
Jules	Bauer			Centre Hospitalier Universitaire de Lille, Lille, France	France		
Diego Fernando	Bautista Rincon			Clinica Valle de Lilli, Valle del Cauca, Colombia	Colombia		
Denisse	Bazan Dow			University of Washington Medical Center - Northwest, Seattle, United States of America	United States of America		
Abigail	Beane			Critical Care Asia - Thailand	Unknown		
Alexandra	Bedossa			Grand Hôpital de l'Est Francilien (Site de Marne-la-Vallée), Jossigny, France	France		
Ker Hong	Bee			Raja Permaisuri Bainun Hospital, Perak, Malaysia	Malaysia		
Husna	Begum			WHO-ISARIC Clinical Characterisation Protocol & SPRINT-SARI, United Kingdom	United Kingdom		
Sylvie	Behilill			INSERM, Paris, France	France		
Albertus	Beishuizen			Medisch Spectrum Twente, Zutphen, Netherlands	Netherlands		
Aleksandr	Beljantsev			Tartu University Hospital, Tartu, Estonia	Estonia		
David	Bellemare			Hôpital de l'Enfant-Jésus, Quebec, Canada	Canada		
Anna	Beltrame			Follow Up Study Working Group, United Kingdom	United Kingdom		
Beatriz Amorim	Beltrão			Sao Camilo Cura D'ars, Fortaleza, Brazil	Brazil		
Marine	Beluze			INSERM, Paris, France	France		
Nicolas	Benech			Centre Hospitalier Universitaire de Lyon - HCL, Lyon, France	France		
Lionel Eric	Benjiman			Sarawak General Hospital, Sarawak, Malaysia	Malaysia		

*First Name and Middle Initial(s)	*Last Name	*Suffix (eg, Jr, III)	Academic Degrees	Institution	Location (city, state/province, country)	Role or Contribution, eg, chair, principal investigator	Group (if more than 1 Group listed in the byline) and/or Subgroup (eg, Steering Committee)
Suzanne	Bennett			University of Cincinnati, Cincinnati, United States of America	United States of America		
Luís	Bento			Hospital de São José -U.U.M., Lisbon, Portugal	Portugal		
Jan-Erik	Berdal			Akershus University Hospital, Nordbyhagen, Norway	Norway		
Delphine	Bergeaud			Centre Hospitalier de Saintonge, Saintes, France	France		
Hazel	Bergin			St Vincents University Hospital, Dublin, Ireland	Ireland		
José Luis	Bernal Sobrino			Hospital 12 de Octubre, Madrid, Spain	Spain		
Giulia	Bertoli			Ospedale Sacro Cuore Don Calabria, Negrar Di Valpolicella, Italy	Italy		
Lorenzo	Bertolino			University of Campania, Carseta, Italy	Italy		
Simon	Bessis			Hôpital Raymond-Poincaré, Garches, France	France		
Sybille	Bevilcaqua			Centre Hospitalier Régional et Universitaire de Nancy - Hôpitaux de Brabois, Nancy, France	France		
Karine	Bezulier			Hôpital de la Timone, Marseille, France	France		
Amar	Bhatt			Ohio State University, Columbus, United States of America	United States of America		
Krishna	Bhavsar			INSERM, Paris, France	France		
Claudia	Bianco			Istituto Mediterraneo per i Trapianti e Terapie ad Alta Specializzazione, Palermo, Italy	Italy		
Farah Nadiah	Bidin			Sungai Buloh Hospital, Selangor, Malaysia	Malaysia		
Felwa	Bin Humaid			King Abdulaziz Medical City, Riyadh, Saudi Arabia	Saudi Arabia		
Mohd Nazlin	Bin Kamarudin			Sungai Buloh Hospital, Selangor, Malaysia	Malaysia		
Zeno	Bisoffi			Department of Infectious, Tropical Diseases and Microbiology, IRCCS Sacro Cuore Don Calabria Hospital, Negrar, Italy	Italy		
Patrick	Biston			Civil Hospital Marie Curie, Charleroi, Belgium	Belgium		
Laurent	Bitker			Hôpital Lyon Sud - HCL, Lyon, France	France		

*First Name and Middle Initial(s)	*Last Name	*Suffix (eg, Jr, III)	Academic Degrees	Institution	Location (city, state/province, country)	Role or Contribution, eg, chair, principal investigator	Group (if more than 1 Group listed in the byline) and/or Subgroup (eg, Steering Committee)
Mustapha	Bittaye			Clinical Services Department Fajara, Gambia	Gambia		
Jonathan	Bitton			The Centre hospitalier universitaire Sainte-Justine, Montreal, Canada	Canada		
Pablo	Blanco-Schweizer			Rio Hortega University Hospital, Valladolid, Spain	Spain		
Catherine	Blier			Hôpital de l'Enfant-Jésus, Quebec, Canada	Canada		
Frank	Bloos			Jena University Hospital, Jena, Germany	Germany		
Mathieu	Blot			Centre Hospitalier Universitaire Mitterrand Dijon-Bourgogne, Dijon, France	France		
Filomena	Boccia			University of Campania, Carseta, Italy	Italy		
Laetitia	Bodenes			Centre Hospitalier Universitaire de Brest, Brest, France	France		
Debby	Bogaert			Centre for Inflammation Research, The Queen's Medical Research Institute, University of Edinburgh, 47 Little France Crescent, Edinburgh, UK	United Kingdom		
Anne-Hélène	Boivin			Centre Hospitalier de Dax - Côte d'Argent, Dax, France	France		
Isabela	Bolaños			Universidad del Cauca, Cauca, Colombia	Colombia		
Pierre-Adrien	Bolze			Hôpital Lyon Sud - HCL, Lyon, France	France		
François	Bompart			INSERM, Paris, France	France		
Aurelius	Bonfasius			Pratama Rada Bolo Hospital, Karitas Hospital and Waikabubak Hospital, Sumba, Indonesia	Indonesia		
Diogo	Borges			Hospital Curry Cabral - Intensive Care Unit - UCIP7, Lisbon, Portugal	Portugal		
Raphaël	Borie			Hôpital Bichat Claude-Bernard AP-HP, Paris, France	France		
Hans Martin	Bosse			University Hospital Dusseldorf, Dusseldorf, Germany	Germany		
Elisabeth	Botelho-Nevers			Centre Hospitalier Universitaire de Saint-Étienne, Saint-Étienne, France	France		
Lila	Bouadma			INSERM, Paris, France	France		
Olivier	Bouchaud			Hôpital Avicenne, Bobigny, France	France		

*First Name and Middle Initial(s)	*Last Name	*Suffix (eg, Jr, III)	Academic Degrees	Institution	Location (city, state/province, country)	Role or Contribution, eg, chair, principal investigator	Group (if more than 1 Group listed in the byline) and/or Subgroup (eg, Steering Committee)
Sabelline	Bouchez			Centre Hospitalier Universitaire de Nantes (Hôpital femme-enfant-adolescent), Nantes, France	France		
Dounia	Bouhmani			Centre hospitalier de l'université de Montréal, Montreal, Canada	Canada		
Damien	Bouhour			Centre Hospitalier de Bourg-en-Bresse, Bourg-en-Bresse, France	France		
Kévin	Bouiller			Centre Hospitalier Universitaire de Besançon, Besançon, France	France		
Laurence	Bouillet			Centre Hospitalier Universitaire Grenoble-Alpes, Grenoble, France	France		
Camile	Bouisse			Centre Hospitalier de Bourg-en-Bresse, Bourg-en-Bresse, France	France		
Latsaniphone	Bountthasavong			Xieng Khouang Provincial Hospital, Phonsavan, Laos	Laos		
Anne-Sophie	Boureau			Centre Hospitalier Universitaire de Nantes (Hôtel-Dieu), Nantes, France	France		
John	Bourke			Galway University Hospital, Galway, Ireland	Ireland		
Maude	Bouscambert			INSERM, Paris, France	France		
Aurore	Bousquet			Hôpital d'Instruction des Armées Bégin, Saint-Mandé, France	France		
Jason	Bouziotis			CUB-Hopital Erasme, Anderlecht, Belgium	Belgium		
Marielle	Boyer-Besseyre			Centre Hospitalier Universitaire de Lille, Lille, France	France		
Maria	Boylan			Galway University Hospital, Galway, Ireland	Ireland		
Fernando Augusto	Bozza			National Institute of Infectious Disease Evandro Chagas, Oswaldo Cruz Foundation (INI-FIOCRUZ), Ministry of Health, and D'Or Institute of Research and Education (IDOR), Rio de Janeiro, Brazil	Brazil		
Axelle	Braconnier			Centre Hospitalier de Mayotte, Mamoudzou, Mayotte	Mayotte		

*First Name and Middle Initial(s)	*Last Name	*Suffix (eg, Jr, III)	Academic Degrees	Institution	Location (city, state/province, country)	Role or Contribution, eg, chair, principal investigator	Group (if more than 1 Group listed in the byline) and/or Subgroup (eg, Steering Committee)
Cynthia	Braga			Centro de Pesquisa Aggeu Magalhães, Fiocruz, Recife, Brazil	Brazil		
Timo	Brandenburger			University Hospital Dusseldorf, Dusseldorf, Germany	Germany		
Filipa	Brás Monteiro			Hospital Egas Moniz, Lisboa, Portugal	Portugal		
Luca	Brazzi			Ospedale Molinette, Torino, Italy	Italy		
Patrick	Breen			Beacon Hospital, Dublin, Ireland	Ireland		
Dorothy	Breen			Cork University Hospital, Cork, Ireland	Ireland		
David	Brewster			Monash University, Melbourne, Australia	Australia		
Kathy	Brickell			St Vincents University Hospital, Dublin, Ireland	Ireland		
Tessa	Broadley			Monash University, Melbourne, Australia	Australia		
Helen	Brotherton			Clinical Services Department Fajara, Gambia	Gambia		
Shaunagh	Browne			Cork University Hospital, Cork, Ireland	Ireland		
Alex	Browne			Nelson Hospital, Nelson, New Zealand	New Zealand		
Nicolas	Brozzi			Cleveland Clinic, Weston, United States of America	United States of America		
Sonja Hjellegjerde	Brunvoll			The Norwegian Corona Cohort, Oslo, Norway	Norway		
Marjolein	Brusse-Keizer			Medisch Spectrum Twente, Zutphen, Netherlands	Netherlands		
Petra	Bryda			Galway University Hospital, Galway, Ireland	Ireland		
Nina	Buchtele			Medical University of Vienna, Vienna, Austria	Austria		
Polina	Bugaeva			Sechenov University, Moscow, Russia	Russia		
Marielle	Buisson			Centre Hospitalier Universitaire Mitterrand Dijon-Bourgogne, Dijon, France	France		
Danilo	Buonsenso			Follow Up Study Working Group, United Kingdom	United Kingdom		
Erlina	Burhan			Persahabatan Hospital, Jakarta, Indonesia	Indonesia		
Ingrid G.	Bustos			Clinica Universidad de La Sabana, Chia, Colombia	Colombia		
Denis	Butnaru			Sechenov University, Moscow, Russia	Russia		

*First Name and Middle Initial(s)	*Last Name	*Suffix (eg, Jr, III)	Academic Degrees	Institution	Location (city, state/province, country)	Role or Contribution, eg, chair, principal investigator	Group (if more than 1 Group listed in the byline) and/or Subgroup (eg, Steering Committee)
André	Cabie			Centre Hospitalier Universitaire de Martinique, Fort-de-France, Saint Martin (French)	Saint Martin (French)		
Susana	Cabral			Centro Hospital e Universitário de Coimbra, Coimbra, Portugal	Portugal		
Joana	Cabrita			Hospital Curry Cabral - Intensive Care Unit - UCIP7, Lisbon, Portugal	Portugal		
Eder	Caceres			Clinica Universidad de La Sabana, Chia, Colombia	Colombia		
Cyril	Cadoz			Centre Hospitalier Régional Metz-Thionville, Metz, France	France		
Rui	Caetano Garcês			Hospital Curry Cabral - Intensive Care Unit - UCIP7, Lisbon, Portugal	Portugal		
Kate	Calligy			Johns Hopkins, Baltimore, United States of America	United States of America		
Jose Andres	Calvache			Universidad del Cauca, Cauca, Colombia	Colombia		
João	Camões			Comissão de Ética - Unidade Local de Saúde de Matosinhos, Porto, Portugal	Portugal		
Valentine	Campana			Centre Hospitalier Universitaire de Martinique, Fort-de-France, Saint Martin (French)	Saint Martin (French)		
Paul	Campbell			Presbyterian Hospital Services, Alberquerque, United States of America	United States of America		
Josie	Campisi			McGill University Health Centre, Montreal, Canada	Canada		
Cecilia	Canepa			Hospital del Mar, Barcelona, Spain	Spain		
Mireia	Cantero			Hospital Puerta de Hierro Majadahonda, Madrid, Spain	Spain		
Janice	Caoili			Makati Medical Centre, Makati, Philippines	The Philippines		
Pauline	Caraux-Paz			Centre Hospitalier Intercommunal Villeneuve-Saint-Georges, Villeneuve-Saint-Georges, France	France		

*First Name and Middle Initial(s)	*Last Name	*Suffix (eg, Jr, III)	Academic Degrees	Institution	Location (city, state/province, country)	Role or Contribution, eg, chair, principal investigator	Group (if more than 1 Group listed in the byline) and/or Subgroup (eg, Steering Committee)
Sheila	Cárcel			Reina Sofia University Hospital, Cordoba, Spain	Spain		
Sofia	Cardoso			Hospital Curry Cabral - Intensive Care Unit - UCIP7, Lisbon, Portugal	Portugal		
Filipe	Cardoso			Hospital Curry Cabral - Intensive Care Unit - UCIP7, Lisbon, Portugal	Portugal		
Filipa	Cardoso			Hospital Curry Cabral - Intensive Care Unit - UCIP7, Lisbon, Portugal	Portugal		
Nelson	Cardoso			Hospital Espírito Santo de Évora, Évora, Portugal	Portugal		
Simone	Carelli			Fondazione Policlinico Universitario Agostino Gemelli IRCCS, Rome, Italy	Italy		
Nicolas	Carlier			Hôpital Cochin AP-HP, Paris, France	France		
Thierry	Carmoi			Hôpital Américain de Paris, Neuilly-sur-Seine, France	France		
Gayle	Carney			Vancouver Island Health, Vancouver, Canada	Canada		
Inês	Carqueja			Comissão de Ética - Unidade Local de Saúde de Matosinhos, Porto, Portugal	Portugal		
Marie-Christine	Carret			Centre Hospitalier Métropole Savoie, Chambéry, France	France		
François Martin	Carrier			Department of Anesthesiology, Centre hospitalier de l'Université de Montréal	Canada		
Ida	Carroll			University Hospital - Limerick, Limerick, Ireland	Ireland		
Gail	Carson			ISARIC, Pandemic Sciences Institute, University of Oxford, Oxford, UK	United Kingdom		
Maire-Laure	Casanova			Centre Hospitalier de Béziers, Béziers, France	France		
Mariana	Cascão			Hospital Curry Cabral - Intensive Care Unit - UCIP7, Lisbon, Portugal	Portugal		
Siobhan	Casey			Galway University Hospital, Galway, Ireland	Ireland		
José	Casimiro			Hospital Curry Cabral - Intensive Care Unit - UCIP7, Lisbon, Portugal	Portugal		

*First Name and Middle Initial(s)	*Last Name	*Suffix (eg, Jr, III)	Academic Degrees	Institution	Location (city, state/province, country)	Role or Contribution, eg, chair, principal investigator	Group (if more than 1 Group listed in the byline) and/or Subgroup (eg, Steering Committee)
Bailey	Cassandra			University of Cincinnati, Cincinnati, United States of America	United States of America		
Silvia	Castañeda			Hospital del Mar, Barcelona, Spain	Spain		
Nidyanara	Castanheira			Instituto de Infectologia Emílio Ribas, Sao Paulo, Brazil	Brazil		
Guylaine	Castor-Alexandre			Hôpital Lariboisière AP-HP, Paris, France	France		
Ivo	Castro			Hospital São Francisco Xavier, Lisbon, Portugal	Portugal		
Ana	Catarino			Centro Hospital e Universitário de Coimbra, Coimbra, Portugal	Portugal		
François-Xavier	Catherine			Centre Hospitalier Universitaire Mitterrand Dijon-Bourgogne, Dijon, France	France		
Paolo	Cattaneo			Ospedale Sacro Cuore Don Calabria, Negrar Di Valpolicella, Italy	Italy		
Roberta	Cavalin			Instituto de Infectologia Emílio Ribas, Sao Paulo, Brazil	Brazil		
Giulio Giovanni	Cavalli			Policlinicodi Orsola Universitàdi Bologna, Bologna, Italy	Italy		
Alexandros	Cavayas			Hospital du Sacre Coeur, Montreal, Canada	Canada		
Adrian	Ceccato			Hospital Universitari Sagrat Cor, Barcelona, Spain	Spain		
Masaneh	Ceesay			Clinical Services Department Fajara, Gambia	Gambia		
Minerva	Cervantes-Gonzalez			INSERM, Paris, France	France		
Muge	Cevik			Makati Medical Centre, Makati, Philippines	The Philippines		
Anissa	Chair			INSERM, Paris, France	France		
Catherine	Chakveatze			Centre Hospitalier de Melun, Melun, France	France		
Adrienne	Chan			Sunnybrook Health Sciences Centre, Toronto, Canada	Canada		
Meera	Chand			Antimicrobial Resistance and Hospital Acquired Infection Department, Public Health England, London, UK	United Kingdom		

*First Name and Middle Initial(s)	*Last Name	*Suffix (eg, Jr, III)	Academic Degrees	Institution	Location (city, state/province, country)	Role or Contribution, eg, chair, principal investigator	Group (if more than 1 Group listed in the byline) and/or Subgroup (eg, Steering Committee)
Jean-Marc	Chapplain			Centre Hospitalier Universitaire Rennes (Hôpital Pontchaillou), Rennes, France	France		
Charlotte	Charpentier			Hôpital Bichat Claude-Bernard AP-HP, Paris, France	France		
Julie	Chas			Hôpital Tenon AP-HP, Paris, France	France		
Jonathan Samuel	Chávez Iñiguez			University of Guadalajara Health Sciences Center, Guadalajara, Mexico	Mexico		
Anjellica	Chen			McGill University Health Centre, Montreal, Canada	Canada		
Yih-Sharng	Chen			National Taiwan University Hospital, Taipei City, Taiwan	Taiwan		
Léo	Chenard			Hôpital Bichat Claude-Bernard AP-HP, Paris, France	France		
Matthew Pellan	Cheng			McGill University Health Centre, Montreal, Canada	Canada		
Antoine	Cheret			Hôpital Kremlin-Bicêtre, Le Kremlin-Bicêtre, France	France		
Thibault	Chiarabini			Hôpital Saint-Antoine AP-HP, Paris, France	France		
Julian	Chica			Clinica Valle de Lilli, Valle del Cauca, Colombia	Colombia		
Suresh Kumar	Chidambaram			National Institutes of Health (NIH), Ministry of Health Malaysia, Setia Alam, Malaysia	Malaysia		
Leong	Chin Tho			Kuala Lumpur Hospital, WPKL, Malaysia	Malaysia		
Catherine	Chirouze			Centre Hospitalier Universitaire de Besançon, Besançon, France	France		
Davide	Chiumello			Ospedale San Paolo, Milan, Italy	Italy		
Sung-Min	Cho			Johns Hopkins, Baltimore, United States of America	United States of America		
Bernard	Cholley			Hôpital Européen Georges-Pompidou AP-HP, Paris, France	France		
Danoy	Chommanam			Salavan Provincial Hospital, Salavan, Laos	Laos		
Marie-Charlotte	Chopin			Centre Hospitalier Universitaire de Lille, Lille, France	France		
Ting Soo	Chow			Pulau Pinang Hospital, Pulau Pinang, Malaysia	Malaysia		

***First Name and Middle Initial(s)**	***Last Name**	***Suffix (eg, Jr, III)**	Academic Degrees	Institution	Location (city, state/province, country)	Role or Contribution, eg, chair, principal investigator	Group (if more than 1 Group listed in the byline) and/or Subgroup (eg, Steering Committee)
Nathaniel	Christy			Salavan Provincial Hospital, Salavan, Laos	Laos		
Hiu Jian	Chua			National Institutes of Health (NIH), Ministry of Health Malaysia, Setia Alam, Malaysia	Malaysia		
Jonathan	Chua			Sungai Buloh Hospital, Selangor, Malaysia	Malaysia		
Jose Pedro	Cidade			Hospital São Francisco Xavier, Lisbon, Portugal	Portugal		
José Miguel	Cisneros Herreros			University Hospital Virgen del Rocío / Institute of Biomedicine of Seville, Seville, Spain	Spain		
Anna	Ciullo			University of Utah, Salt Lake City, United States of America	United States of America		
Jennifer	Clarke			Beaumont Hospital, Dublin, Ireland	Ireland		
Rolando	Claure-Del Granado			Caja Nacional De Salud, Trinidad, Bolivia	Bolivia		
Sara	Clohisey			Oxford University (ISARIC4C), Oxford, United Kingdom	United Kingdom		
Cassidy	Codan			Foothills Medical Centre, Calgary, Canada	Canada		
Caitriona	Cody			Connolly Hospital Blanchardstown, Dublin, Ireland	Ireland		
Jennifer	Coles			Monash University, Melbourne, Australia	Australia		
Gwenhaël	Colin			Centre Hospitalier Départemental Vendée, La Roche-sur-Yon, France	France		
Michael	Collins			Allegheny General Hospital, Pittsburgh, United States of America	United States of America		
Pamela	Combs			University of Chicago, Chicago, United States of America	United States of America		
Jennifer	Connolly			Cork University Hospital, Cork, Ireland	Ireland		
Marie	Connor			Oxford University (ISARIC4C), Oxford, United Kingdom	United Kingdom		
Anne	Conrad			Centre Hospitalier Universitaire de Lyon - HCL, Lyon, France	France		
Elaine	Conway			University Hospital - Limerick, Limerick, Ireland	Ireland		

*First Name and Middle Initial(s)	*Last Name	*Suffix (eg, Jr, III)	Academic Degrees	Institution	Location (city, state/province, country)	Role or Contribution, eg, chair, principal investigator	Group (if more than 1 Group listed in the byline) and/or Subgroup (eg, Steering Committee)
Graham S.	Cooke			Department of Infectious Disease, Imperial College London, London, UK	United Kingdom		
Hugues	Cordel			Hôpital Avicenne, Bobigny, France	France		
Amanda	Corley			University of Queensland, Brisbane, Australia	Australia		
Sabine	Cornelis			CUB-Hopital Erasme, Anderlecht, Belgium	Belgium		
Alexander Daniel	Cornet			Medisch Spectrum Twente, Zutphen, Netherlands	Netherlands		
Arianne Joy	Corpuz			Al-Adan Hospital, Hadiya, Kuwait	Kuwait		
Andrea	Cortegiani			University Hospital Policlinico Paolo Giaccone, Palermo, Italy	Italy		
Grégory	Corvaisier			Centre Hospitalier Bretagne Atlantique, Vannes, France	France		
Camille	Couffignal			INSERM, Paris, France	France		
Sandrine	Couffin-Cadiergues			INSERM, Paris, France	France		
Roxane	Courtois			Centre Hospitalier de Cholet, Cholet, France	France		
Stéphanie	Cousse			Hôpital Jacques Monod, Le Havre, France	France		
Rachel	Cregan			Children's Health Ireland, Dublin, Ireland	Ireland		
Cosimo	Cristella			PREPARE and RECOVER EU Consortium, Belgium	Belgium		
Gloria	Crowl			Michael Garron Hospital, Toronto, Canada	Canada		
Jonathan	Crump			University of Utah, Salt Lake City, United States of America	United States of America		
Claudina	Cruz			Hospital de Curry Cabral - Infectious Diseases, Lisbon, Portugal	Portugal		
Juan Luis	Cruz Bermúdez			Hospital 12 de Octubre, Madrid, Spain	Spain		
Jaime	Cruz Rojo			Hospital 12 de Octubre, Madrid, Spain	Spain		
Marc	Csete			Mount Sinai Medical Center, Miami, FL, United States of America	United States of America		
Ailbhe	Cullen			Beaumont Hospital, Dublin, Ireland	Ireland		
Matthew	Cummings			Columbia University, New York, United States of America	United States of America		
Gerard	Curley			Beaumont Hospital, Dublin, Ireland	Ireland		

*First Name and Middle Initial(s)	*Last Name	*Suffix (eg, Jr, III)	Academic Degrees	Institution	Location (city, state/province, country)	Role or Contribution, eg, chair, principal investigator	Group (if more than 1 Group listed in the byline) and/or Subgroup (eg, Steering Committee)
Elodie	Curlier			Centre Hospitalier Universitaire de Guadeloupe, Pointe-à-Pitre, Guadeloupe	Guadeloupe		
Colleen	Curran			Cork University Hospital, Cork, Ireland	Ireland		
Paula	Custodio			Instituto de Infectologia Emílio Ribas, Sao Paulo, Brazil	Brazil		
Ana	da Silva Filipe			MRC-University of Glasgow Centre for Virus Research, 464 Bearsden Road, Glasgow, UK	United Kingdom		
Charlene	Da Silveira			INSERM, Paris, France	France		
Al-Awwab	Dabaliz			UH Cleveland Hospital, Cleveland, United States of America	United States of America		
Andrew	Dagens			ISARIC, Pandemic Sciences Institute, University of Oxford, Oxford, UK	United Kingdom		
John Arne	Dahl			The Norwegian Corona Cohort, Oslo, Norway	Norway		
Darren	Dahly			University Hospital - Waterford, Waterford, Ireland	Ireland		
Umberto	D'Alessandro			Clinical Services Department Fajara, Gambia	Gambia		
Peter	Daley			Memorial University, St. John's, Newfoundland, St John's, Canada	Canada		
Jo	Dalton			Oxford University (ISARIC4C), Oxford, United Kingdom	United Kingdom		
Heidi	Dalton			University of Queensland, Brisbane, Australia	Australia		
Seamus	Daly			Galway University Hospital, Galway, Ireland	Ireland		
Juliana	Damas			Hospital Curry Cabral - Intensive Care Unit - UCIP7, Lisbon, Portugal	Portugal		
Nick	Daneman			Sunnybrook Health Sciences Centre, Toronto, Canada	Canada		
Emmanuelle A	Dankwa			Department of Statistics, University of Oxford, Oxford, UK	United Kingdom		
Jorge	Dantas			Hospital Curry Cabral - Intensive Care Unit - UCIP7, Lisbon, Portugal	Portugal		
Frédérick	D'Aragon			Centre hospitalier Universitaire de Sherbrooke, Sherbrooke, Canada	Canada		

*First Name and Middle Initial(s)	*Last Name	*Suffix (eg, Jr, III)	Academic Degrees	Institution	Location (city, state/province, country)	Role or Contribution, eg, chair, principal investigator	Group (if more than 1 Group listed in the byline) and/or Subgroup (eg, Steering Committee)
Menno	de Jong			PREPARE and RECOVER EU Consortium, Belgium	Belgium		
Gillian	de Loughry			University Hospital - Waterford, Waterford, Ireland	Ireland		
Diego	de Mendoza			Hospital Universitari Sagrat Cor, Barcelona, Spain	Spain		
Etienne	De Montmollin			Hôpital Bichat Claude-Bernard AP-HP, Paris, France	France		
Rafael Freitas	de Oliveira França			Centro de Pesquisa Aggeu Magalhães, Fiocruz, Recife, Brazil	Brazil		
Ana Isabel	de Pinho Oliveira			Centro Hospitalar de Tondela-Viseu, Viseu, Portugal	Portugal		
Rosanna	De Rosa			Monaldi Hospital, Napoli, Italy	Italy		
Cristina	De Rose			Università Cattolica del Sacro Cuore, Rome, Italy	Italy		
Thushan	de Silva			The Florey Institute for Host-Pathogen Interactions, Department of Infection, Immunity and Cardiovascular Disease, University of Sheffield, Sheffield, UK	United Kingdom		
Peter	de Vries			Tergooi Hospital, Hilversum, Netherlands	Netherlands		
Jillian	Deacon			St Christopher's Hospital for Children, Philadelphia, United States of America	United States of America		
David	Dean			Piedmont Atlanta Hospital, Atlanta, Georgia, United States of America	United States of America		
Alexa	Debard			Hôpital Purpan, Toulouse, France	France		
Bianca	DeBenedictis			Beaumont Hospital, Dublin, Ireland	Ireland		
Marie-Pierre	Debray			INSERM, Paris, France	France		
Nathalie	DeCastro			Hôpital Saint-Louis AP-HP, Paris, France	France		
William	Dechert			Brantford General Hospital, Brantford, Canada	Canada		
Romain	Decours			Centre Hospitalier Départemental Vendée, La Roche-sur-Yon, France	France		

*First Name and Middle Initial(s)	*Last Name	*Suffix (eg, Jr, III)	Academic Degrees	Institution	Location (city, state/province, country)	Role or Contribution, eg, chair, principal investigator	Group (if more than 1 Group listed in the byline) and/or Subgroup (eg, Steering Committee)
Eve	Defous			Centre Hospitalier Emile Roux, Le Puy-en-Velay, France	France		
Isabelle	Delacroix			Centre Hospitalier intercommunal de Créteil, Créteil, France	France		
Eric	Delaveuve			Hôpital Bel-Air, Thionville, France	France		
Karen	Delavigne			Centre Hospitalier Universitaire Toulouse (IUCT), Toulouse, France	France		
Nathalie M.	Delfos			Alrijne Hospital, Leiden, Netherlands	Netherlands		
Ionna	Deligiannis			Uniklinik University Hospital, Frankfurt, Germany	Germany		
Andrea	Dell'Amore			Policlinico of Padova, Padova, Italy	Italy		
Christelle	Delmas			INSERM, Paris, France	France		
Pierre	Delobel			Hôpital Purpan, Toulouse, France	France		
Corine	Delsing			Medisch Spectrum Twente, Zutphen, Netherlands	Netherlands		
Elisa	Demonchy			Centre Hospitalier Universitaire de Nice (Hôpital Archet), Nice, France	France		
Emmanuelle	Denis			ISARIC Global Support Centre, Oxford, United Kingdom	United Kingdom		
Dominique	Deplanque			INSERM, Paris, France	France		
Pieter	Depuydt			Universitair Ziekenhuis, Gent, Belgium	Belgium		
Mehul	Desai			INOVA Fairfax Medical Center, Fairfax, Virginia, United States of America	United States of America		
Diane	Descamps			INSERM, Paris, France	France		
Mathilde	Desvallées			INSERM, Paris, France	France		
Santi	Dewayanti			National Cardiovascular Center Harapan Kita Jakarta Indonesia, Jakarta, Indonesia	Indonesia		
Pathik	Dhangar			All India Institute of Medical Sciences, Rishikesh, India	India		
Alpha	Diallo			INSERM, Paris, France	France		
Sylvain	Diamantis			Centre Hospitalier de Melun, Melun, France	France		
André	Dias			Hospital de Curry Cabral - Infectious Diseases, Lisbon, Portugal	Portugal		

*First Name and Middle Initial(s)	*Last Name	*Suffix (eg, Jr, III)	Academic Degrees	Institution	Location (city, state/province, country)	Role or Contribution, eg, chair, principal investigator	Group (if more than 1 Group listed in the byline) and/or Subgroup (eg, Steering Committee)
Fernanda	Dias Da Silva			INSERM, Paris, France	France		
Juan Jose	Diaz			Hospital Universitario Dr Negrín, Las Palmas, Spain	Spain		
Rodrigo	Diaz			Clinica Las Condes, Santiago, Chile	Chile		
Priscila	Diaz			Hospital Professor Doutor Fernando Fonseca, Amadora, Portugal	Portugal		
Bakary K	Dibba			Clinical Services Department Fajara, Gambia	Gambia		
Kévin	Didier			Centre Hospitalier Universitaire de Reims, Reims, France	France		
Jean-Luc	Diehl			Hôpital Européen Georges-Pompidou AP-HP, Paris, France	France		
Wim	Dieperink			University Medical Center Groningen, Groningen, Netherlands	Netherlands		
Jérôme	Dimet			Centre Hospitalier Mont-de-Marsan, Mont-de-Marsan, France	France		
Vincent	Dinot			Centre Hospitalier Régional Metz-Thionville, Metz, France	France		
Fara	Diop			Centre Hospitalier Intercommunal Villeneuve-Saint-Georges, Villeneuve-Saint-Georges, France	France		
Alphonsine	Diouf			INSERM, Paris, France	France		
Yael	Dishon			Rambam Hospital, Haifa, Israel	Israel		
Félix	Djossou			Centre Hospitalier Andrée Rosemon, Cayenne, French Guiana	French Guiana		
Annemarie B.	Docherty			Centre for Medical Informatics, The Usher Institute, University of Edinburgh, Edinburgh, UK	United Kingdom		
Helen	Doherty			Beaumont Hospital, Dublin, Ireland	Ireland		
Arjen M	Dondorp			CCA Network	Unknown		
Christl A.	Donnelly			Department of Statistics, University of Oxford, Oxford, UK	United Kingdom		
Sean	Donohue			Galway University Hospital, Galway, Ireland	Ireland		
Yoann	Donohue			Galway University Hospital, Galway, Ireland	Ireland		

*First Name and Middle Initial(s)	*Last Name	*Suffix (eg, Jr, III)	Academic Degrees	Institution	Location (city, state/province, country)	Role or Contribution, eg, chair, principal investigator	Group (if more than 1 Group listed in the byline) and/or Subgroup (eg, Steering Committee)
Peter	Doran			St Vincents University Hospital, Dublin, Ireland	Ireland		
Céline	Dorival			INSERM, Paris, France	France		
Eric	D'Ortenzio			INSERM, Paris, France	France		
Yash	Doshi			Long COVID India - Terna Specialty Hospital and Research Centre, Mumbai, India	India		
Phouvieng	Douangdala			Luang Namtha Provincial Hospital, Luang Namtha, Laos	Laos		
James Joshua	Douglas			Lions Gate Hospital, Vancouver, Canada	Canada		
Renee	Douma			Flevoziekenhuis, Almere, Netherlands	Netherlands		
Nathalie	Dournon			Hôpital Avicenne, Bobigny, France	France		
Joanne	Downey			St Vincents University Hospital, Dublin, Ireland	Ireland		
Mark	Downing			St Joseph's Health Center, Sherbrooke, Canada	Canada		
Thomas	Drake			Oxford University (ISARIC4C), Oxford, United Kingdom	United Kingdom		
Aoife	Driscoll			University Hospital - Limerick, Limerick, Ireland	Ireland		
Amiel A.	Dror			Bar-Ilan University, Ramat Gan, Israel	Israel		
Murray	Dryden			National Institute for Communicable Diseases, Johannesburg, South Africa	South Africa		
Claudio	Duarte Fonseca			Centro de Pesquisa Aggeu Magalhães, Fiocruz, Recife, Brazil	Brazil		
Vincent	Dubee			Centre Hospitalier Universitaire d'Angers, Angers, France	France		
François	Dubos			INSERM, Paris, France	France		
Audrey	Dubot-Pérès			Salavan Provincial Hospital, Salavan, Laos	Laos		
Alexandre	Ducancelle			Centre Hospitalier Universitaire d'Angers, Angers, France	France		
Toni	Duculan			Houston Methodist Hospital, Texas, United States of America	United States of America		
Susanne	Dudman			Oslo University Hospital, Oslo, Norway	Norway		

*First Name and Middle Initial(s)	*Last Name	*Suffix (eg, Jr, III)	Academic Degrees	Institution	Location (city, state/province, country)	Role or Contribution, eg, chair, principal investigator	Group (if more than 1 Group listed in the byline) and/or Subgroup (eg, Steering Committee)
Abhijit	Duggal			Cleveland Clinic, Ohio, Ohio, OH, United States of America	United States of America		
Paul	Dunand			Hôpital Bel-Air, Thionville, France	France		
Jake	Dunning			ISARIC, Pandemic Sciences Institute, University of Oxford, Oxford, UK	United Kingdom		
Mathilde	Duplaix			Hospital du Sacre Coeur, Montreal, Canada	Canada		
Emanuele	Durante-Mangoni			University of Campania, Carseta, Italy	Italy		
Lucian	Durham III			Medical College of Wisconsin, Wisconsin, United States of America	United States of America		
Bertrand	Dussol			Hôpital de la Conception, Marseille, France	France		
Juliette	Duthoit			Centre Hospitalier de Tourcoing, Tourcoing, France	France		
Xavier	Duval			INSERM, Paris, France	France		
Anne Margarita	Dyrhol-Riise			Oslo University Hospital, Oslo, Norway	Norway		
Sim Choon	Ean			Pulau Pinang Hospital, Pulau Pinang, Malaysia	Malaysia		
Marco	Echeverria-Villalobos			Ohio State University, Columbus, United States of America	United States of America		
Michael	Edelstein			Follow Up Study Working Group, United Kingdom	United Kingdom		
Siobhan	Egan			University Hospital - Limerick, Limerick, Ireland	Ireland		
Linn Margrete	Eggesbø			The Norwegian Corona Cohort, Oslo, Norway	Norway		
Carla	Eira			Centro Hospitalar de Tondela-Viseu, Viseu, Portugal	Portugal		
Mohammed	El Sanharawi			Centre Hospitalier De Chateaudun, Route De Jallans, 28200 Chateaudun, France	France		
Subbarao	Elapavaluru			Allegheny General Hospital, Pittsburgh, United States of America	United States of America		
Brigitte	Elharrar			Centre Hospitalier intercommunal de Créteil, Créteil, France	France		
Jacobien	Ellerbroek			Reinier de Graaf Gasthuis, Delft, Netherlands	Netherlands		
Merete	Ellingjord-Dale			The Norwegian Corona Cohort, Oslo, Norway	Norway		

*First Name and Middle Initial(s)	*Last Name	*Suffix (eg, Jr, III)	Academic Degrees	Institution	Location (city, state/province, country)	Role or Contribution, eg, chair, principal investigator	Group (if more than 1 Group listed in the byline) and/or Subgroup (eg, Steering Committee)
Loubna	Elotmani			CHU Carémeau, Nimes, France	France		
Philippine	Eloy			INSERM, Paris, France	France		
Tarek	Elshazly			UH Cleveland Hospital, Cleveland, United States of America	United States of America		
Iqbal	Elyazar			Pratama Rada Bolo Hospital, Karitas Hospital and Waikabubak Hospital, Sumba, Indonesia	Indonesia		
Isabelle	Enderle			Centre Hospitalier Universitaire Rennes (Hôpital Sud), Rennes, France	France		
Tomoyuki	Endo			Tohoku Medical and Pharmaceutical University, Sendai, Japan	Japan		
Chan Chee	Eng			Kuala Lumpur Hospital, WPKL, Malaysia	Malaysia		
Ilka	Engelmann			Centre Hospitalier Universitaire de Lille, Lille, France	France		
Vincent	Enouf			INSERM, Paris, France	France		
Olivier	Epaulard			Centre Hospitalier Universitaire Grenoble-Alpes, Grenoble, France	France		
Martina	Escher			ISARIC, Pandemic Sciences Institute, University of Oxford, Oxford, UK	United Kingdom		
Mariano	Esperatti			Mar del Plata Medical Foundation Private Community Hospital, Mar Del Plata, Argentina	Argentina		
Hélène	Esperou			INSERM, Paris, France	France		
Catarina	Espírito Santo			Hospital Curry Cabral - Intensive Care Unit - UCIP7, Lisbon, Portugal	Portugal		
Marina	Esposito-Farese			INSERM, Paris, France	France		
João	Estevão			Hospital Curry Cabral - Intensive Care Unit - UCIP7, Lisbon, Portugal	Portugal		
Manuel	Etienne			INSERM, Paris, France	France		
Anna Greti	Everding			Hospitales Puerta de Hierro, Jalisco, Mexico	Mexico		
Mirjam	Evers			Canisius Wilhelmina Ziekenhuis, Nijmenjen, Netherlands	Netherlands		
Marc	Fabre			Centre Hospitalier Pierre Oudot, Bourgoin-Jallieu, France	France		

*First Name and Middle Initial(s)	*Last Name	*Suffix (eg, Jr, III)	Academic Degrees	Institution	Location (city, state/province, country)	Role or Contribution, eg, chair, principal investigator	Group (if more than 1 Group listed in the byline) and/or Subgroup (eg, Steering Committee)
Isabelle	Fabre			Centre Hospitalier Universitaire de Guadeloupe, Pointe-à-Pitre, Guadeloupe	Guadeloupe		
Ismaila	Fadera			Clinical Services Department Fajara, Gambia	Gambia		
Amna	Faheem			North York General Hospital, Toronto, Canada	Canada		
Arabella	Fahy			Tallaght University Hospital, Dublin, Ireland	Ireland		
Cameron J.	Fairfield			Oxford University (ISARIC4C), Oxford, United Kingdom	United Kingdom		
Pedro	Faria			Hospital Curry Cabral - Intensive Care Unit - UCIP7, Lisbon, Portugal	Portugal		
Hanan	Fateena			Sungai Buloh Hospital, Selangor, Malaysia	Malaysia		
Arie Zainul	Fatoni			Adult ICU Saiful Anwar Hospital, Malang, Indonesia	Indonesia		
Karine	Faure			Centre Hospitalier Universitaire de Lille, Lille, France	France		
Raphaël	Favory			Centre Hospitalier Universitaire de Lille, Lille, France	France		
Mohamed	Fayed			University of California San Francisco - Fresno, Fresno, United States of America	United States of America		
Niamh	Feely			University Hospital, Kerry, Ireland	Ireland		
Jorge	Fernandes			Hospital Curry Cabral - Intensive Care Unit - UCIP7, Lisbon, Portugal	Portugal		
Marília Andreia	Fernandes			Hospital Curry Cabral - Intensive Care Unit - UCIP7, Lisbon, Portugal	Portugal		
Susana	Fernandes			Hospital Santa Maria, Centro Hospitalar Universitário Lisboa Norte, Amadora, Portugal	Portugal		
François-Xavier	Ferrand			Centre Hospitalier Techer, Calais, France	France		
Eglantine	Ferrand Devouge			Centre Hospitalier Universitaire Rouen (Center Hospitalier Universitaire de Rouen), Rouen, France	France		
Joana	Ferrão			Hospital Curry Cabral - Intensive Care Unit - UCIP7, Lisbon, Portugal	Portugal		

*First Name and Middle Initial(s)	*Last Name	*Suffix (eg, Jr, III)	Academic Degrees	Institution	Location (city, state/province, country)	Role or Contribution, eg, chair, principal investigator	Group (if more than 1 Group listed in the byline) and/or Subgroup (eg, Steering Committee)
Mário	Ferraz			Hospital Curry Cabral - Intensive Care Unit - UCIP7, Lisbon, Portugal	Portugal		
Sílvia	Ferreira			Comissão de Ética - Unidade Local de Saúde de Matosinhos, Porto, Portugal	Portugal		
Bernardo	Ferreira			Hospital Curry Cabral - Intensive Care Unit - UCIP7, Lisbon, Portugal	Portugal		
Benigno	Ferreira			Hospitales Puerta de Hierro, Jalisco, Mexico	Mexico		
Isabel	Ferreira			Hospital Professor Doutor Fernando Fonseca, Amadora, Portugal	Portugal		
Nicolas	Ferriere			Centre Hospitalier Universitaire de Brest, Brest, France	France		
Céline	Ficko			Hôpital d'Instruction des Armées Bégin, Saint-Mandé, France	France		
Claudia	Figueiredo-Mello			Instituto de Infectologia Emílio Ribas, Brazil	Brazil		
William	Finlayson			St Bernard's Hospital, Gibraltar, Gibraltar	Gibraltar		
Thomas	Flament			Centre Hospitalier Régional et Universitaire de Tours, Tours, France	France		
Tom	Fletcher			Liverpool School of Tropical Medicine, Liverpool, UK	United Kingdom		
Aline-Marie	Florence			Hôpital Bichat Claude-Bernard AP-HP, Paris, France	France		
Letizia Lucia	Florio			University of Campania, Carseta, Italy	Italy		
Deirdre	Flynn			Cork University Hospital, Cork, Ireland	Ireland		
Brigid	Flynn			University of Kansas Medical Center, Kansas, United States of America	United States of America		
Jean	Foley			Cork University Hospital, Cork, Ireland	Ireland		
Victor	Fomin			Sechenov University, Moscow, Russia	Russia		
Tatiana	Fonseca			Centro Hospitalar Vila Nova de Gaia/Espinho, Espinho, Portugal	Portugal		
Patricia	Fontela			The Montreal Children's Hospital, Montreal, Canada	Canada		
Karen	Forrest			Clinical Services Department Fajara, Gambia	Gambia		

*First Name and Middle Initial(s)	*Last Name	*Suffix (eg, Jr, III)	Academic Degrees	Institution	Location (city, state/province, country)	Role or Contribution, eg, chair, principal investigator	Group (if more than 1 Group listed in the byline) and/or Subgroup (eg, Steering Committee)
Simon	Forsyth			University of Queensland, Brisbane, Australia	Australia		
Denise	Foster			Vancouver General Hospital, Vancouver, Canada	Canada		
Giuseppe	Foti			Ospedale San Gerardo, Monza, Italy	Italy		
Erwan	Fourn			Hôpital Foch, Suresnes, France	France		
Robert A.	Fowler			University of Toronto, Toronto, Canada	Canada		
Marianne	Fraher			Bon Secours Hospital, Cork, Ireland	Ireland		
Diego	Franch-Llasat			Hospital Verge de la Cinta, Tortosa, Spain	Spain		
John F.	Fraser			University of Queensland, Brisbane, Australia	Australia		
Christophe	Fraser			ISARIC Global Support Centre, Oxford, United Kingdom	United Kingdom		
Marcela Vieira	Freire			Centro de Pesquisa Aggeu Magalhães, Fiocruz, Recife, Brazil	Brazil		
Ana	Freitas Ribeiro			Instituto de Infectologia Emílio Ribas, Sao Paulo, Brazil	Brazil		
Caren	Friedrich			Hospital Escola da Universidade Federal de Pelotas, Pelotas, Brazil	Brazil		
Ricardo	Fritz			Instituto Nacional Del Tórax, Santiago, Chile	Chile		
Stéphanie	Fry			Hôpital Albert Calmette, Lille, France	France		
Nora	Fuentes			Mar del Plata Medical Foundation Private Community Hospital, Mar Del Plata, Argentina	Argentina		
Masahiro	Fukuda			Saiseikai Senri Hospital, Tochigi, Japan	Japan		
Argin	G			Manipal Hospital Whitefield, Bangalore, India	India		
Valérie	Gaborieau			Centre Hospitalier de Pau, Pau, France	France		
Rostane	Gaci			Centre Hospitalier Régional Metz-Thionville, Metz, France	France		
Massimo	Gagliardi			University of Campania, Carseta, Italy	Italy		
Jean-Charles	Gagnard			Hôpital privé d'Antony, Antony, France	France		
Amandine	Gagneux-Brunon			Centre Hospitalier Universitaire de Saint-Étienne, Saint-Étienne, France	France		
Abdou	Gai			Clinical Services Department Fajara, Gambia	Gambia		
Sérgio	Gaião			São João Hospital Centre, Porto, Portugal	Portugal		
Linda	Gail Skeie			Oslo University Hospital, Oslo, Norway	Norway		

***First Name and Middle Initial(s)**	***Last Name**	***Suffix (eg, Jr, III)**	Academic Degrees	Institution	Location (city, state/province, country)	Role or Contribution, eg, chair, principal investigator	Group (if more than 1 Group listed in the byline) and/or Subgroup (eg, Steering Committee)
Phil	Gallagher			St Vincents University Hospital, Dublin, Ireland	Ireland		
Carrol	Gamble			Oxford University (ISARIC4C), Oxford, United Kingdom	United Kingdom		
Yasmin	Gani			Sungai Buloh Hospital, Selangor, Malaysia	Malaysia		
Arthur	Garan			Beth Israel Deaconess Medical Center, Boston, United States of America	United States of America		
Rebekha	Garcia			University of California San Francisco - Fresno, Fresno, United States of America	United States of America		
Noelia	García Barrio			Hospital 12 de Octubre, Madrid, Spain	Spain		
Julia	Garcia-Diaz			Ochsner Clinic Foundation, New Orleans, United States of America	United States of America		
Esteban	Garcia-Gallo			Clinica Universidad de La Sabana, Chia, Colombia	Colombia		
Navya	Garimella			University of Washington Medical Center - Northwest, Seattle, United States of America	United States of America		
Denis	Garot			Centre Hospitalier Régional et Universitaire de Tours, Tours, France	France		
Valérie	Garrait			Centre Hospitalier intercommunal de Créteil, Créteil, France	France		
Anatoliy	Gavrylov			Lugansk State Medical University - Department of Internal Medicine No2, Lugansk, Ukraine	Ukraine		
Alexandre	Gaymard			INSERM, Paris, France	France		
Johannes	Gebauer			Klinikum Passau, Germant, Germany	Germany		
Eva	Geraud			Centre Hospitalier Emile Roux, Le Puy-en-Velay, France	France		
Louis	Gerbaud Morlaes			Centre Hospitalier Mont-de-Marsan, Mont-de-Marsan, France	France		
Nuno	Germano			Hospital Curry Cabral - Intensive Care Unit - UCIP7, Lisbon, Portugal	Portugal		
Praveen Kumar	Ghisulal			Cleveland Clinic Abu Dhabi, Abu Dhabi, United Arab Emirates	United Arab Emirates		

*First Name and Middle Initial(s)	*Last Name	*Suffix (eg, Jr, III)	Academic Degrees	Institution	Location (city, state/province, country)	Role or Contribution, eg, chair, principal investigator	Group (if more than 1 Group listed in the byline) and/or Subgroup (eg, Steering Committee)
Jade	Ghosn			INSERM, Paris, France	France		
Marco	Giani			Ospedale San Gerardo, Monza, Italy	Italy		
Tristan	Gigante			INSERM, Paris, France	France		
Elaine	Gilroy			Mater Misericordiae University, Dublin, Ireland	Ireland		
Guillermo	Giordano			Centre Hospitalier Henri Duffaut, Avignon, France	France		
Michelle	Girvan			Oxford University (ISARIC4C), Oxford, United Kingdom	United Kingdom		
Valérie	Gissot			Centre Hospitalier Régional et Universitaire de Tours, Tours, France	France		
Daniel	Glikman			The Baruch Padeh Medical Center Poriya, Tiberias, Israel	Israel		
Petr	Glybochko			Sechenov University, Moscow, Russia	Russia		
Eric	Gnall			Lankenau Institute of Medical Research, Wynnewood, United States of America	United States of America		
Geraldine	Goco			The Hospital for Sick Children (SickKids), Toronto, Canada	Canada		
François	Goehringer			Centre Hospitalier Régional et Universitaire de Nancy - Hôpitaux de Brabois, Nancy, France	France		
Siri	Goepel			University Hospital of Tubingen, Tubingen, Germany	Germany		
Jean-Christophe	Goffard			CUB-Hôpital Erasme, Bruxelles, Belgium	Belgium		
Jin Yi	Goh			Permai Hospital, Johor, Malaysia	Malaysia		
Jonathan	Golob			University of Michigan Schools of Medicine & Public Health, AnnArbor, United States of America	United States of America		
Rui	Gomes			Hospital Garcia de Orta, Almada, Portugal	Portugal		
Kyle	Gomez			St Bernard's Hospital, Gibraltar, Gibraltar	Gibraltar		

*First Name and Middle Initial(s)	*Last Name	*Suffix (eg, Jr, III)	Academic Degrees	Institution	Location (city, state/province, country)	Role or Contribution, eg, chair, principal investigator	Group (if more than 1 Group listed in the byline) and/or Subgroup (eg, Steering Committee)
Joan	Gómez-Junyent			Department of Infectious Diseases, Hospital del Mar, Infectious Pathology and Antimicrobial Research Group (IPAR), Institut Hospital del Mar d'Investigacions Mèdiques (IMIM), Universitat Autònoma de Barcelona (UAB), CEXS-Universitat Pompeu Fabra, Barcelona, Spain.	Spain		
Marie	Gominet			Hôpital d'Instruction des Armées Bégin, Saint-Mandé, France	France		
Alicia	Gonzalez			Ohio State University, Columbus, United States of America	United States of America		
Patricia	Gordon			Wexford General Hospital, Wexford, Ireland	Ireland		
Yanay	Gorelik			Bar-Ilan University, Ramat Gan, Israel	Israel		
Isabelle	Gorenne			INSERM, Paris, France	France		
Laure	Goubert			Hôpital Jacques Monod, Le Havre, France	France		
Cécile	Goujard			Hôpital Kremlin-Bicêtre, Le Kremlin-Bicêtre, France	France		
Tiphaine	Goulenok			Hôpital Bichat Claude-Bernard AP-HP, Paris, France	France		
Margarite	Grable			Baylor Scott & White Health, Temple, United States of America	United States of America		
Jeronimo	Graf			Clinica Alemana DeSantiago, Santiago, Chile	Chile		
Edward Wilson	Grandin			Beth Israel Deaconess Medical Center, Boston, United States of America	United States of America		
Pascal	Granier			Centre Hospitalier du Pays d'Aix, Aix-en-Provence, France	France		
Giacomo	Grasselli			Fondazione IRCCS Ca, Milan, Italy	Italy		
Christopher A.	Green			Institute of Microbiology and Infection, University of Birmingham, Birmingham, UK	United Kingdom		
Courtney	Greene			Children's Health Ireland, Dublin, Ireland	Ireland		
William	Greenhalf			Oxford University (ISARIC4C), Oxford, United Kingdom	United Kingdom		

*First Name and Middle Initial(s)	*Last Name	*Suffix (eg, Jr, III)	Academic Degrees	Institution	Location (city, state/province, country)	Role or Contribution, eg, chair, principal investigator	Group (if more than 1 Group listed in the byline) and/or Subgroup (eg, Steering Committee)
Segolène	Greffe			Centre Hospitalier Universitaire Ambroise-Paré, Boulogne-Billancourt, France	France		
Domenico Luca	Grieco			Fondazione Policlinico Universitario Agostino Gemelli IRCCS, Rome, Italy	Italy		
Matthew	Griffee			University of Utah, Salt Lake City, United States of America	United States of America		
Fiona	Griffiths			Oxford University (ISARIC4C), Oxford, United Kingdom	United Kingdom		
Ioana	Grigoras			Grigore T Popa University of Medicine and Pharmacy, Bucharest, Romania	Romania		
Albert	Groenendijk			Erasmus Medical Centre, Rotterdam, Netherlands	Netherlands		
Heidi	Gruner			Hospital de Curry Cabral - Internal Medicine, Lisbon, Portugal	Portugal		
Yusing	Gu			McGill University Health Centre, Montreal, Canada	Canada		
Jérémie	Guedj			INSERM, Paris, France	France		
Martin	Guego			Centre Hospitalier Techer, Calais, France	France		
Anne-Marie	Guerguerian			The Hospital for Sick Children (SickKids), Toronto, Canada	Canada		
Daniela	Guerreiro			Hospital Curry Cabral - Intensive Care Unit - UCIP7, Lisbon, Portugal	Portugal		
Romain	Guery			Centre Hospitalier Universitaire de Nantes (Hôpital femme-enfant-adolescent), Nantes, France	France		
Anne	Guillaumot			Centre Hospitalier Régional et Universitaire de Nancy - Hôpitaux de Brabois, Nancy, France	France		
Laurent	Guilleminault			Centre Hospitalier Universitaire Toulouse (Larrey), Toulouse, France	France		
Maisa	Guimarães de Castro			Hospital de Amor, Sao Paulo, Brazil	Brazil		

*First Name and Middle Initial(s)	*Last Name	*Suffix (eg, Jr, III)	Academic Degrees	Institution	Location (city, state/province, country)	Role or Contribution, eg, chair, principal investigator	Group (if more than 1 Group listed in the byline) and/or Subgroup (eg, Steering Committee)
Thomas	Guimard			Centre Hospitalier Départemental Vendée, La Roche-sur-Yon, France	France		
Marieke	Haalboom			Medisch Spectrum Twente, Zutphen, Netherlands	Netherlands		
Daniel	Haber			Rambam Hospital, Haifa, Israel	Israel		
Ali	Hachemi			Centre Hospitalier de Soissons, Soissons, France	France		
Nadir	Hadri			Hôpital Kremlin-Bicêtre, Le Kremlin-Bicêtre, France	France		
Sheeba	Hakak			University Hospital - Waterford, Waterford, Ireland	Ireland		
Adam	Hall			Red Deer Regional Hospital, Red Deer, Canada	Canada		
Matthew	Hall			Big Data Institute, Nuffield Department of Medicine, University of Oxford, Oxford, UK	United Kingdom		
Sophie	Halpin			Oxford University (ISARIC4C), Oxford, United Kingdom	United Kingdom		
Ansley	Hamer			McLeod Healthcare System, Florence, United States of America	United States of America		
Raph L.	Hamers			Murni Teguh Memorial Hospital and Bunda Thamrin Hospital, North Sumatera, Indonesia	Indonesia		
Rebecca	Hamidfar			Centre Hospitalier Universitaire Grenoble-Alpes, Grenoble, France	France		
Bato	Hammarström			The Norwegian Corona Cohort, Oslo, Norway	Norway		
Terese	Hammond			Providence Saint John's Health Centre, Santa Monica, United States of America	United States of America		
Naomi	Hammond			Monash University, Melbourne, Australia	Australia		
Lim Yuen	Han			Kluang Hospital, Johor, Malaysia	Malaysia		
Rashan	Haniffa			CCA Network	Unknown		
Kok Wei	Hao			Kluang Hospital, Johor, Malaysia	Malaysia		
Hayley	Hardwick			Oxford University (ISARIC4C), Oxford, United Kingdom	United Kingdom		

*First Name and Middle Initial(s)	*Last Name	*Suffix (eg, Jr, III)	Academic Degrees	Institution	Location (city, state/province, country)	Role or Contribution, eg, chair, principal investigator	Group (if more than 1 Group listed in the byline) and/or Subgroup (eg, Steering Committee)
Ewen M.	Harrison			Centre for Medical Informatics, The Usher Institute, University of Edinburgh, Edinburgh, UK	United Kingdom		
Janet	Harrison			Oxford University (ISARIC4C), Oxford, United Kingdom	United Kingdom		
Samuel Bernard Ekow	Harrison			Kintampo Health Research Centre, Kintampo, Ghana	Ghana		
Alan	Hartman			Northwell Health, New York, United States of America	United States of America		
Junaid	Hashmi			Our lady of Lourdes Drogheda, Drogheda, Ireland	Ireland		
Ailbhe	Hayes			Sligo University Hospital (Saolta), Sligo, Ireland	Ireland		
Leanne	Hays			St Vincents University Hospital, Dublin, Ireland	Ireland		
Jan	Heerman			AZ Maria Middelares, Gent, Belgium	Belgium		
Lars	Heggelund			Drammen Hospital, Drammen, Norway	Norway		
Ross	Hendry			Oxford University (ISARIC4C), Oxford, United Kingdom	United Kingdom		
Martina	Hennessy			St James's Hospital, Dublin, Ireland	Ireland		
Aquiles Rodrigo	Henriquez-Trujillo			Universidad de Las Américas, Quito, Ecuador	Ecuador		
Maxime	Hentzien			Centre Hospitalier Universitaire de Reims, Reims, France	France		
Diana	Hernandez			Universidad del Cauca, Cauca, Colombia	Colombia		
Daniel	Herr			University of Maryland, Baltimore, United States of America	United States of America		
Andrew	Hershey			Lancaster General Health, Pennsylvania, United States of America	United States of America		
Liv	Hesstvedt			Oslo University Hospital, Oslo, Norway	Norway		
Astarini	Hidayah			PICU Saiful Anwar Hospital, Malang, Indonesia	Indonesia		
Eibhlin	Higgins			Galway University Hospital, Galway, Ireland	Ireland		

*First Name and Middle Initial(s)	*Last Name	*Suffix (eg, Jr, III)	Academic Degrees	Institution	Location (city, state/province, country)	Role or Contribution, eg, chair, principal investigator	Group (if more than 1 Group listed in the byline) and/or Subgroup (eg, Steering Committee)
Rupert	Higgins			ISARIC Global Support Centre, Oxford, United Kingdom	United Kingdom		
Samuel	Hinton			University of Queensland, Brisbane, Australia	Australia		
Hiroaki	Hiraiwa			Nagoya University Hospital, Nagoya, Japan	Japan		
Haider	Hirkani			Long COVID India - Terna Specialty Hospital and Research Centre, Mumbai, India	India		
Hikombo	Hitoto			Centre Hospitalier Le Mans, Le Mans, France	France		
Antonia	Ho			Medical Research Council-University of Glasgow Centre for Virus Research, Glasgow, UK	United Kingdom		
Yi Bin	Ho			Sultanah Bahiyah Hospital, Kedah, Malaysia	Malaysia		
Alexandre	Hoctin			INSERM, Paris, France	France		
Isabelle	Hoffmann			INSERM, Paris, France	France		
Wei Han	Hoh			Tuanku Ja'afar, Negeri Sembilan, Malaysia	Malaysia		
Oscar	Hoiting			Canisius Wilhelmina Ziekenhuis, Nijmenjen, Netherlands	Netherlands		
Rebecca	Holt			University of Alabama at Birmingham Hospital, Birmingham, United States of America	United States of America		
Jan Cato	Holter			Oslo University Hospital, Oslo, Norway	Norway		
Peter	Horby			ISARIC, Pandemic Sciences Institute, University of Oxford, Oxford, UK	United Kingdom		
Juan Pablo	Horcajada			Hospital del Mar, Barcelona, Spain	Spain		
Ikram	Houas			INSERM, Paris, France	France		
Catherine L.	Hough			US NHLBI PETAL Network, Boston, United States of America	United States of America		
Stuart	Houltham			Cleveland Clinic, Ohio, Ohio, OH, United States of America	United States of America		
Jimmy Ming-Yang	Hsu			McGill University Health Centre, Montreal, Canada	Canada		
Jean-Sébastien	Hulot			INSERM, Paris, France	France		
Stella	Huo			University of California - San Francisco (UCSF), San Francisco, United States of America	United States of America		

*First Name and Middle Initial(s)	*Last Name	*Suffix (eg, Jr, III)	Academic Degrees	Institution	Location (city, state/province, country)	Role or Contribution, eg, chair, principal investigator	Group (if more than 1 Group listed in the byline) and/or Subgroup (eg, Steering Committee)
Abby	Hurd			St. Joseph's Healthcare Hamilton, Hamilton, Canada	Canada		
Samreen	Ijaz			Virology Reference Department, National Infection Service, Public Health England, Colindale Avenue, London, United Kingdom	United Kingdom		
M. Arfan	Ikram			Erasmus Medical Centre, Rotterdam, Netherlands	Netherlands		
Carlos Cañada	Illana			La Paz Hospital, Madrid, Spain	Spain		
Patrick	Imbert			Centre Hospitalier Annecy Genevois, Annecy, France	France		
Hugo	Inácio			Hospital Curry Cabral - Intensive Care Unit - UCIP7, Lisbon, Portugal	Portugal		
Carmen	Infante Dominguez			University Hospital Virgen del Rocío / Institute of Biomedicine of Seville, Seville, Spain	Spain		
Yun Sii	Ing			Sungai Buloh Hospital, Selangor, Malaysia	Malaysia		
Elias	Iosifidis			Hippokration Hospital, Thessaloniki, Greece	Greece		
Mariachiara	Ippolito			University Hospital Policlinico Paolo Giaccone, Palermo, Italy	Italy		
Sarah	Isgett			McLeod Healthcare System, Florence, United States of America	United States of America		
Tiago	Isidoro			Hospital Curry Cabral - Intensive Care Unit - UCIP7, Lisbon, Portugal	Portugal		
Nadiah	Ismail			Kuala Lumpur Hospital, WPKL, Malaysia	Malaysia		
Margaux	Isnard			Centre Hospitalier Métropole Savoie, Chambéry, France	France		
Mette Stausland	Istre			The Norwegian Corona Cohort, Oslo, Norway	Norway		
Junji	Itai			Hiroshima University, Hiroshima, Japan	Japan		
Daniel	Ivulich			Hospital Aleman, Buenos Aires, Argentina	Argentina		
Danielle	Jaafar			Centre Hospitalier Intercommunal Villeneuve-Saint-Georges, Villeneuve-Saint-Georges, France	France		
Salma	Jaafoura			INSERM, Paris, France	France		

*First Name and Middle Initial(s)	*Last Name	*Suffix (eg, Jr, III)	Academic Degrees	Institution	Location (city, state/province, country)	Role or Contribution, eg, chair, principal investigator	Group (if more than 1 Group listed in the byline) and/or Subgroup (eg, Steering Committee)
Julien	Jabot			Centre Hospitalier Félix-Guyon, Saint-Denis, Réunion	Réunion		
Clare	Jackson			Oxford University (ISARIC4C), Oxford, United Kingdom	United Kingdom		
Abubacarr	Jagne			Clinical Services Department Fajara, Gambia	Gambia		
Victoria	Janes			PREPARE and RECOVER EU Consortium, Belgium	Belgium		
Stéphane	Jaureguiberry			Hôpital Kremlin-Bicêtre, Le Kremlin-Bicêtre, France	France		
Denise	Jaworsky			Mills Memorial Hospital, Terrace, Canada	Canada		
Florence	Jego			Centre Hospitalier Métropole Savoie, Chambéry, France	France		
Anilawati Mat	Jelani			Raja Perempuan Zainab II Hospital, Kelantan, Malaysia	Malaysia		
Synne	Jenum			Oslo University Hospital, Oslo, Norway	Norway		
Ruth	Jimbo-Sotomayor			Universidad de Las Américas, Quito, Ecuador	Ecuador		
Ong Yiaw	Joe			Kuala Lumpur Hospital, WPKL, Malaysia	Malaysia		
Ruth Noemí	Jorge García			Hospital Nuestra Señora de Gracia, Zaragoza, Spain	Spain		
Silje Bakken	Jørgensen			The Norwegian Corona Cohort, Oslo, Norway	Norway		
Mark	Joseph			Carilion Clinic, Roanoke, United States of America	United States of America		
Cédric	Joseph			Centre Hospitalier Universitaire Amiens-Picardie, Amiens, France	France		
Swosti	Joshi			St Christopher's Hospital for Children, Philadelphia, United States of America	United States of America		
Mercé	Jourdain			Centre Hospitalier Universitaire de Lille, Lille, France	France		
Philippe	Jouvet			The Centre hospitalier universitaire Sainte-Justine, Montreal, Canada	Canada		
Anna	Jung			Providence Saint John's Health Centre, Santa Monica, United States of America	United States of America		

*First Name and Middle Initial(s)	*Last Name	*Suffix (eg, Jr, III)	Academic Degrees	Institution	Location (city, state/province, country)	Role or Contribution, eg, chair, principal investigator	Group (if more than 1 Group listed in the byline) and/or Subgroup (eg, Steering Committee)
Hanna	Jung			Kyungpook National University Hospital, Daegu, South Korea	South Korea		
Dafsah	Juzar			National Cardiovascular Center Harapan Kita Jakarta Indonesia, Jakarta, Indonesia	Indonesia		
Ouifiya	Kafif			INSERM, Paris, France	France		
Florentia	Kaguelidou			INSERM, Paris, France	France		
Neerusha	Kaisbain			Permai Hospital, Johor, Malaysia	Malaysia		
Thavamany	Kaleesvran			Tuanku Fauziah Hospital, Perlis, Malaysia	Malaysia		
Sabina	Kali			INSERM, Paris, France	France		
Alina	Kalicinska			Consortium IMGEN, Piaseczno, Poland	Poland		
Karl Trygve	Kalleberg			The Norwegian Corona Cohort, Oslo, Norway	Norway		
Smaragdi	Kalomoiri			Sotiria General Hospital, Athens, Greece	Greece		
Muhammad Aisar Ayadi	Kamaluddin			Sungai Buloh Hospital, Selangor, Malaysia	Malaysia		
Zul Amali Che	Kamaruddin			Tawau Hospital, Sabah, Malaysia	Malaysia		
Nadiah	Kamarudin			Melaka Hospital, Melaka, Malaysia	Malaysia		
Darshana Hewa	Kandamby			Princess Margaret Hospital, Kwai Hung, China	China		
Kong Yeow	Kang			Pulau Pinang Hospital, Pulau Pinang, Malaysia	Malaysia		
Dyah	Kanyawati			Sanglah General Hospital (Paediatric), Bali, Indonesia	Indonesia		
Pratap	Karpayah			Sungai Buloh Hospital, Selangor, Malaysia	Malaysia		
Christiana	Kartsonaki			MRC Population Health Research Unit, Clinical Trials Service Unit and Epidemiological Studies Unit, Nuffield Department of Population Health, University of Oxford, Oxford, UK	United Kingdom		
Daisuke	Kasugai			Nagoya University Hospital, Nagoya, Japan	Japan		
Kevin	Katz			North York General Hospital, Toronto, Canada	Canada		
Christy	Kay			Washington University in St. Louis, St Louis, Missouri, United States of America	United States of America		
Lamees	Kayyali			Galway University Hospital, Galway, Ireland	Ireland		

*First Name and Middle Initial(s)	*Last Name	*Suffix (eg, Jr, III)	Academic Degrees	Institution	Location (city, state/province, country)	Role or Contribution, eg, chair, principal investigator	Group (if more than 1 Group listed in the byline) and/or Subgroup (eg, Steering Committee)
Seán	Keating			Oxford University (ISARIC4C), Oxford, United Kingdom	United Kingdom		
Pulak	Kedia			Long COVID India - Terna Specialty Hospital and Research Centre, Mumbai, India	India		
Claire	Kelly			Galway University Hospital, Galway, Ireland	Ireland		
Yvelynne	Kelly			Tallaght University Hospital, Dublin, Ireland	Ireland		
Andrea	Kelly			St James's Hospital, Dublin, Ireland	Ireland		
Niamh	Kelly			Cork University Hospital, Cork, Ireland	Ireland		
Aoife	Kelly			Mater Misericordiae University, Dublin, Ireland	Ireland		
Sadie	Kelly			ISARIC Global Support Centre, Oxford, United Kingdom	United Kingdom		
Maeve	Kelsey			Cork University Hospital, Cork, Ireland	Ireland		
Kalynn	Kennon			ISARIC Global Support Centre, Oxford, United Kingdom	United Kingdom		
Sommay	Keomany			Salavan Provincial Hospital, Salavan, Laos	Laos		
Maeve	Kernan			Galway University Hospital, Galway, Ireland	Ireland		
Younes	Kerroumi			Groupe Hospitalier Diaconesses Croix Saint-Simon, Paris, France	France		
Sharma	Keshav			Wexford General Hospital, Wexford, Ireland	Ireland		
Imrana	Khalid			Unity Health Toronto, Toronto, Canada	Canada		
Antoine	Khalil			INSERM, Paris, France	France		
Irfan	Khan			Presbyterian Hospital Services, Alberquerque, United States of America	United States of America		
Krish	Kherajani			Long COVID India - Terna Specialty Hospital and Research Centre, Mumbai, India	India		
Michelle E.	Kho			McMaster University, Hamilton, Canada	Canada		
Saye	Khoo			Department of Pharmacology, University of Liverpool, Liverpool, UK	United Kingdom		
Ryan	Khoo			Lahad Datu Hospital, Sabah, Malaysia	Malaysia		
Denisa	Khoo			Sungai Buloh Hospital, Selangor, Malaysia	Malaysia		
Khor How	Kiat			Kluang Hospital, Johor, Malaysia	Malaysia		

*First Name and Middle Initial(s)	*Last Name	*Suffix (eg, Jr, III)	Academic Degrees	Institution	Location (city, state/province, country)	Role or Contribution, eg, chair, principal investigator	Group (if more than 1 Group listed in the byline) and/or Subgroup (eg, Steering Committee)
Yuri	Kida			University of Utah, Salt Lake City, United States of America	United States of America		
Peter	Kiiza			Sunnybrook Health Sciences Centre, Toronto, Canada	Canada		
Beathe	Kiland Granerud			Oslo University Hospital, Oslo, Norway	Norway		
Anders Benjamin	Kildal			University Hospital of North Norway, Tromso, Norway	Norway		
Jae Burm	Kim			Keimyung University Dong San Hospital, Daegu, South Korea	South Korea		
Antoine	Kimmoun			Centre Hospitalier Régional et Universitaire de Nancy - Hôpitaux de Brabois, Nancy, France	France		
Detlef	Kindgen-Milles			University Hospital Dusseldorf, Dusseldorf, Germany	Germany		
Nobuya	Kitamura			Kimitsu Chuo Hospital, Chiba, Japan	Japan		
Eyrun Floerecke Kjetland	Kjetland			The Norwegian Corona Cohort, Oslo, Norway	Norway		
Paul	Klenerman			Nuffield Department of Medicine, Peter Medawar Building for Pathogen Research, University of Oxford, UK	United Kingdom		
Rob	Klont			Medisch Spectrum Twente, Zutphen, Netherlands	Netherlands		
Gry	Kloumann Bekken			Drammen Hospital, Drammen, Norway	Norway		
Stephen R	Knight			Oxford University (ISARIC4C), Oxford, United Kingdom	United Kingdom		
Robin	Kobbe			University Children's Hospital, University Medical Center Hamburg-Eppendorf, Hamburg, Germany	Germany		
Chamira	Kodippily			CCA Network	Unknown		
Malte	Kohns Vasconcelos			University Hospital Dusseldorf, Dusseldorf, Germany	Germany		

*First Name and Middle Initial(s)	*Last Name	*Suffix (eg, Jr, III)	Academic Degrees	Institution	Location (city, state/province, country)	Role or Contribution, eg, chair, principal investigator	Group (if more than 1 Group listed in the byline) and/or Subgroup (eg, Steering Committee)
Mamoru	Komatsu			Obihiro-Kosei General Hospital, Obihiro, Japan	Japan		
Volkan	Korten			Marmara University Hospital, Istanbul, Turkey	Turkey		
Arsène	Kpangon			Centre Hospitalier Andrée Rosemon, Cayenne, French Guiana	French Guiana		
Karolina	Krawczyk			St Vincents University Hospital, Dublin, Ireland	Ireland		
Sudhir	Krishnan			Cleveland Clinic, Ohio, Ohio, OH, United States of America	United States of America		
Vinothini	Krishnan			Sungai Buloh Hospital, Selangor, Malaysia	Malaysia		
Oksana	Kruglova			Lugansk State Medical University - Department of Internal Medicine No2, Lugansk, Ukraine	Ukraine		
Pei Xuan	Kuan			Sungai Buloh Hospital, Selangor, Malaysia	Malaysia		
Deepali	Kumar			University Health Network, Toronto, Canada	Canada		
Ganesh	Kumar			Kuala Lumpur Hospital, WPKL, Malaysia	Malaysia		
Dinesh	Kuriakose			Our lady of Lourdes Drogheda, Drogheda, Ireland	Ireland		
Ethan	Kurtzman			Hartford HealthCare, Hartford, United States of America	United States of America		
Neurinda Permata	Kusumastuti			University Airlangga Hospital (Paediatric), Surabaya, Indonesia	Indonesia		
Demetrios	Kutsogiannis			Royal Alexandra Hospital, Edmonton, Canada	Canada		
Galyna	Kutsyna			Lugansk State Medical University - Department of Internal Medicine No2, Lugansk, Ukraine	Ukraine		
Konstantinos	Kyriakoulis			Sotiria General Hospital, Athens, Greece	Greece		
Marie	Lachatre			Hôpital Cochin AP-HP, Paris, France	France		
Marie	Lacoste			Centre Hospitalier Alpes-Leman, Contamine-sur-Arve, France	France		
John G.	Laffey			Galway University Hospital, Galway, Ireland	Ireland		
Nadhem	Lafhej			Hôpital Bichat Claude-Bernard AP-HP, Paris, France	France		

*First Name and Middle Initial(s)	*Last Name	*Suffix (eg, Jr, III)	Academic Degrees	Institution	Location (city, state/province, country)	Role or Contribution, eg, chair, principal investigator	Group (if more than 1 Group listed in the byline) and/or Subgroup (eg, Steering Committee)
Marie	Lagrange			Centre Hospitalier Félix-Guyon, Saint-Denis, Réunion	Réunion		
Fabrice	Laine			Centre Hospitalier Universitaire Rennes (Hôpital Pontchaillou), Rennes, France	France		
Olivier	Lairez			Centre Hospitalier Universitaire Toulouse (Rangueil), Toulouse, France	France		
Antonio	Lalueza			Hospital 12 de Octubre, Madrid, Spain	Spain		
Marc	Lambert			Hôpital Albert Calmette, Lille, France	France		
Marie	Langelot-Richard			Centre Hospitalier Bretagne Atlantique, Vannes, France	France		
Vincent	Langlois			Hôpital Jacques Monod, Le Havre, France	France		
Eka Yudha	Lantang			Prof Dr R. D. Kandou Central Hospital (Adult), Manado, Indonesia	Indonesia		
Marina	Lanza			Instituto de Infectologia Emílio Ribas, Sao Paulo, Brazil	Brazil		
Cédric	Laouénan			Université de Paris, France	France		
Samira	Laribi			INSERM, Paris, France	France		
Delphine	Lariviere			Centre Hospitalier Bretagne Atlantique, Vannes, France	France		
Stéphane	Lasry			Hôpital Américain de Paris, Neuilly-sur-Seine, France	France		
Sakshi	Lath			Long COVID India - Terna Specialty Hospital and Research Centre, Mumbai, India	India		
Odile	Launay			Hôpital Cochin AP-HP, Paris, France	France		
Didier	Laureillard			Centre Hospitalier Universitaire de Nîmes, Nîmes, France	France		
Yoan	Lavie-Badie			Centre Hospitalier Universitaire Toulouse (Rangueil), Toulouse, France	France		
Andy	Law			The Roslin Institute, University of Edinburgh, Edinburgh, UK	United Kingdom		
Teresa	Lawrence			University of Alberta Adult ICU, Edmonton, Canada	Canada		

*First Name and Middle Initial(s)	*Last Name	*Suffix (eg, Jr, III)	Academic Degrees	Institution	Location (city, state/province, country)	Role or Contribution, eg, chair, principal investigator	Group (if more than 1 Group listed in the byline) and/or Subgroup (eg, Steering Committee)
Cassie	Lawrence			Wellington Regional Hospital, Wellington, New Zealand	New Zealand		
Minh	Le			INSERM, Paris, France	France		
Clément	Le Bihan			Centre Hospitalier Universitaire de Montpellier, Montpellier, France	France		
Cyril	Le Bris			Centre Hospitalier de Béziers, Béziers, France	France		
Georges	Le Falher			Centre Hospitalier de Béziers, Béziers, France	France		
Lucie	Le Fevre			Hôpital Bichat Claude-Bernard AP-HP, Paris, France	France		
Quentin	Le Hingrat			INSERM, Paris, France	France		
Marion	Le Maréchal			Centre Hospitalier Universitaire Grenoble-Alpes, Grenoble, France	France		
Soizic	Le Mestre			INSERM, Paris, France	France		
Gwenaël	Le Moal			Centre Hospitalier Universitaire de Poitiers, Poitiers, France	France		
Vincent	Le Moing			Centre Hospitalier Universitaire de Montpellier, Montpellier, France	France		
Hervé	Le Nagard			INSERM, Paris, France	France		
Ema	Leal			Hospital de Curry Cabral - Infectious Diseases, Lisbon, Portugal	Portugal		
Marta	Leal Santos			Hospital de Curry Cabral - Infectious Diseases, Lisbon, Portugal	Portugal		
Todd C.	Lee			McGill University Health Centre, Montreal, Canada	Canada		
Su Hwan	Lee			Severance Hospital, Seoul, South Korea	South Korea		
James	Lee			ISARIC Global Support Centre, Oxford, United Kingdom	United Kingdom		
Heng Gee	Lee			Queen Elizabeth Hospital, Sabah, Malaysia	Malaysia		
Biing Horng	Lee			Raja Permaisuri Bainun Hospital, Perak, Malaysia	Malaysia		
Yi Lin	Lee			Sungai Buloh Hospital, Selangor, Malaysia	Malaysia		
Jennifer	Lee			ISARIC Global Support Centre, Oxford, United Kingdom	United Kingdom		

*First Name and Middle Initial(s)	*Last Name	*Suffix (eg, Jr, III)	Academic Degrees	Institution	Location (city, state/province, country)	Role or Contribution, eg, chair, principal investigator	Group (if more than 1 Group listed in the byline) and/or Subgroup (eg, Steering Committee)
Gary	Leeming			Oxford University (ISARIC4C), Oxford, United Kingdom	United Kingdom		
Laurent	Lefebvre			Centre Hospitalier du Pays d'Aix, Aix-en-Provence, France	France		
Bénédicte	Lefebvre			Hôpital Saint-Antoine AP-HP, Paris, France	France		
Benjamin	Lefèvre			Centre Hospitalier Régional et Universitaire de Nancy - Hôpitaux de Brabois, Nancy, France	France		
Sylvie	LeGac			Hôpital Bichat Claude-Bernard AP-HP, Paris, France	France		
Jean-Daniel	Lelievre			Hôpital Henri-Mondor, Créteil, France	France		
François	Lellouche			University Institute of Cardiology and Respirology, Quebec, Canada	Canada		
Adrien	Lemaignen			Centre Hospitalier Régional et Universitaire de Tours, Tours, France	France		
Véronique	Lemee			Centre Hospitalier Universitaire Rouen (Center Hospitalier Universitaire de Rouen), Rouen, France	France		
Anthony	Lemeur			Centre Hospitalier de Cholet, Cholet, France	France		
Gretchen	Lemmink			University of Cincinnati, Cincinnati, United States of America	United States of America		
Ha Sha	Lene			Sultanah Nur Zahirah Hospital, Terengganu, Malaysia	Malaysia		
Jenny	Lennon			Children's Health Ireland, Dublin, Ireland	Ireland		
Rafael	León			Reina Sofia University Hospital, Cordoba, Spain	Spain		
Marc	Leone			Hôpital de la Timone, Marseille, France	France		
Quentin	Lepiller			Centre Hospitalier Universitaire de Besançon, Besançon, France	France		
François-Xavier	Lescure			INSERM, Paris, France	France		
Olivier	Lesens			Centre Hospitalier Universitaire Gabriel Montpied, Clermont-Ferrand, France	France		

*First Name and Middle Initial(s)	*Last Name	*Suffix (eg, Jr, III)	Academic Degrees	Institution	Location (city, state/province, country)	Role or Contribution, eg, chair, principal investigator	Group (if more than 1 Group listed in the byline) and/or Subgroup (eg, Steering Committee)
Mathieu	Lesouhaitier			Centre Hospitalier Universitaire Rennes (Hôpital Pontchaillou), Rennes, France	France		
Amy	Lester-Grant			St Vincents University Hospital, Dublin, Ireland	Ireland		
Andrew	Letizia			Salavan Provincial Hospital, Salavan, Laos	Laos		
Sophie	Letrou			Hôpital Bichat Claude-Bernard AP-HP, Paris, France	France		
Yves	Levy			INSERM, Paris, France	France		
Bruno	Levy			Centre Hospitalier Régional et Universitaire de Nancy - Hôpitaux de Brabois, Nancy, France	France		
Claire	Levy-Marchal			INSERM, Paris, France	France		
Katarzyna	Lewandowska			Institute of TB and Lung Diseases, Warsaw, Poland	Poland		
Erwan	L'Her			Centre Hospitalier Universitaire de Brest, Brest, France	France		
Gianluigi	Li Bassi			University of Queensland, Brisbane, Australia	Australia		
Janet	Liang			Waitemata District Health Board, Auckland, New Zealand	New Zealand		
Geoffrey	Liegeon			Hôpital Saint-Louis AP-HP, Paris, France	France		
Wei Shen	Lim			Nottingham University Hospitals NHS Trust, Nottingham, United Kingdom	United Kingdom		
Kah Chuan	Lim			Sungai Buloh Hospital, Selangor, Malaysia	Malaysia		
Chantre	Lima			Hospital Espírito Santo de Évora, Évora, Portugal	Portugal		
Lim	Lina			Melaka Hospital, Melaka, Malaysia	Malaysia		
Bruno	Lina			INSERM, Paris, France	France		
Andreas	Lind			Oslo University Hospital, Oslo, Norway	Norway		
Maja Katherine	Lingad			Angeles University Foundation Medical Center, Angeles, Philippines	The Philippines		
Guillaume	Lingas			INSERM, Paris, France	France		
Sylvie	Lion-Daolio			Centre Hospitalier Universitaire Amiens-Picardie, Amiens, France	France		

*First Name and Middle Initial(s)	*Last Name	*Suffix (eg, Jr, III)	Academic Degrees	Institution	Location (city, state/province, country)	Role or Contribution, eg, chair, principal investigator	Group (if more than 1 Group listed in the byline) and/or Subgroup (eg, Steering Committee)
Samantha	Lissauer			Malawi-Liverpool Wellcome Trust, Blantyre, Malawi	Malawi		
Keibun	Liu			Saiseikai Utsunomiya Hospital, Tochigi, Japan	Japan		
Marine	Livrozet			Hôpital Européen Georges-Pompidou AP-HP, Paris, France	France		
Patricia	Lizotte			University Institute of Cardiology and Respirology, Quebec, Canada	Canada		
Antonio	Loforte			Policlinicodi Orsola Universitàdi Bologna, Bologna, Italy	Italy		
Navy	Lolong			Persahabatan Hospital, Jakarta, Indonesia	Indonesia		
Leong Chee	Loon			Kuala Lumpur Hospital, WPKL, Malaysia	Malaysia		
Diogo	Lopes			Hospital Curry Cabral - Intensive Care Unit - UCIP7, Lisbon, Portugal	Portugal		
Dalia	Lopez-Colon			University of Florida, Gainesville, United States of America	United States of America		
Jose W.	Lopez-Revilla			Instituto Nacional del Niño San Borja, Lima, Peru	Peru		
Anthony L.	Loschner			Carilion Clinic, Roanoke, United States of America	United States of America		
Paul	Loubet			Centre Hospitalier Universitaire de Nîmes, Nîmes, France	France		
Bouchra	Loufti			Centre Hospitalier Mont-de-Marsan, Mont-de-Marsan, France	France		
Guillame	Louis			Centre Hospitalier Régional Metz-Thionville, Metz, France	France		
Silvia	Lourenco			Hospital Espírito Santo de Évora, Évora, Portugal	Portugal		
Lara	Lovelace-Macon			University of Washington Medical Center - Northwest, Seattle, United States of America	United States of America		
Lee Lee	Low			Sultanah Bahiyah Hospital, Kedah, Malaysia	Malaysia		
Marije	Lowik			Medisch Spectrum Twente, Zutphen, Netherlands	Netherlands		
Jia Shyi	Loy			Melaka Hospital, Melaka, Malaysia	Malaysia		

*First Name and Middle Initial(s)	*Last Name	*Suffix (eg, Jr, III)	Academic Degrees	Institution	Location (city, state/province, country)	Role or Contribution, eg, chair, principal investigator	Group (if more than 1 Group listed in the byline) and/or Subgroup (eg, Steering Committee)
Jean Christophe	Lucet			INSERM, Paris, France	France		
Carlos	Lumbreras Bermejo			Hospital 12 de Octubre, Madrid, Spain	Spain		
Carlos M.	Luna			Hospital de Clínicas, Buenos Aires, Argentina	Argentina		
Olguta	Lungu			Grigore T Popa University of Medicine and Pharmacy, Bucharest, Romania	Romania		
Miles	Lunn			ISARIC, Pandemic Sciences Institute, University of Oxford, Oxford, UK	United Kingdom		
Liem	Luong			Hôpital Cochin AP-HP, Paris, France	France		
Nestor	Luque			Hospital Emergencia Ate Vitarte, Lima, Peru	Peru		
Dominique	Luton			Hôpital Bichat Claude-Bernard AP-HP, Paris, France	France		
Olavi	Maasikas			Tartu University Hospital, Tartu, Estonia	Estonia		
Sara	Machado			Hospital Curry Cabral - Intensive Care Unit - UCIP7, Lisbon, Portugal	Portugal		
Moïse	Machado			Grand Hôpital de l'Est Francilien (Site de Marne-la-Vallée), Jossigny, France	France		
Gabriel	Macheda			Centre Hospitalier Annecy Genevois, Annecy, France	France		
Guillermo	Maestro de la Calle			Hospital 12 de Octubre, Madrid, Spain	Spain		
Rafael	Mahieu			Centre Hospitalier Universitaire d'Angers, Angers, France	France		
Sophie	Mahy			Centre Hospitalier Universitaire Mitterrand Dijon-Bourgogne, Dijon, France	France		
Ana Raquel	Maia			Hospital Professor Doutor Fernando Fonseca, Amadora, Portugal	Portugal		
Lars S.	Maier			Klinik und Poliklinik für Innere Medizin II, University Hospital Regensburg, Kiel, Germany	Germany		
Mylène	Maillet			Centre Hospitalier Annecy Genevois, Annecy, France	France		

*First Name and Middle Initial(s)	*Last Name	*Suffix (eg, Jr, III)	Academic Degrees	Institution	Location (city, state/province, country)	Role or Contribution, eg, chair, principal investigator	Group (if more than 1 Group listed in the byline) and/or Subgroup (eg, Steering Committee)
Thomas	Maitre			Centre Hospitalier intercommunal de Créteil, Créteil, France	France		
Maximilian	Malfertheiner			Klinik und Poliklinik für Innere Medizin II, University Hospital Regensburg, Kiel, Germany	Germany		
Nadia	Malik			William Osler Health Sciences System - Etobicoke General Hospital, Toronto, Canada	Canada		
Paddy	Mallon			St Vincents University Hospital, Dublin, Ireland	Ireland		
Fernando	Maltez			Hospital de Curry Cabral - Infectious Diseases, Lisbon, Portugal	Portugal		
Denis	Malvy			INSERM, Paris, France	France		
Victoria	Manda			Hôpital Lariboisière AP-HP, Paris, France	France		
Jose M.	Mandei			Prof Dr R. D. Kandou Central Hospital (Paediatric), Manado, Indonesia	Indonesia		
Laurent	Mandelbrot			Hôpital Louis-Mourier, Colombes, France	France		
Frank	Manetta			Northwell Health, New York, United States of America	United States of America		
Julie	Mankikian			Centre Hospitalier Régional et Universitaire de Tours, Tours, France	France		
Edmund	Manning			Cork University Hospital, Cork, Ireland	Ireland		
Aldric	Manuel			Centre Hospitalier Annecy Genevois, Annecy, France	France		
Ceila	Maria Sant`Ana Malaque			Instituto de Infectologia Emílio Ribas, Sao Paulo, Brazil	Brazil		
Flávio	Marino			Hospital Vila Franca de Xira, Lisbon, Portugal	Portugal		
Samuel	Markowicz			Centre Hospitalier Universitaire de Guadeloupe, Pointe-à-Pitre, Guadeloupe	Guadeloupe		
Ana	Marques			Centro Hospital e Universitário de Coimbra, Coimbra, Portugal	Portugal		
Catherine	Marquis			Centre hospitalier Universitaire de Sherbrooke, Sherbrooke, Canada	Canada		

*First Name and Middle Initial(s)	*Last Name	*Suffix (eg, Jr, III)	Academic Degrees	Institution	Location (city, state/province, country)	Role or Contribution, eg, chair, principal investigator	Group (if more than 1 Group listed in the byline) and/or Subgroup (eg, Steering Committee)
Brian	Marsh			Mater Misericordiae University, Dublin, Ireland	Ireland		
Megan	Marshal			Connolly Hospital Blanchardstown, Dublin, Ireland	Ireland		
John	Marshall			Unity Health Toronto, Toronto, Canada	Canada		
Celina Turchi	Martelli			Centro de Pesquisa Aggeu Magalhães, Fiocruz, Recife, Brazil	Brazil		
Dori-Ann	Martin			Alberta Children's Hospital, Calgary, Canada	Canada		
Emily	Martin			University of Michigan Schools of Medicine & Public Health, AnnArbor, United States of America	United States of America		
Guillaume	Martin-Blondel			Hôpital Purpan, Toulouse, France	France		
F. Eduardo	Martinez			Monash University, Melbourne, Australia	Australia		
Martin	Martinot			Centre Hospitalier de Colmar, Colmar, France	France		
Alejandro	Martín-Quiros			Emergency Department. Hospital Universitario La Paz - IdiPAZ	Spain		
João	Martins			Centro Hospital e Universitário de Coimbra, Coimbra, Portugal	Portugal		
Ana	Martins			Hospital Curry Cabral - Intensive Care Unit - UCIP7, Lisbon, Portugal	Portugal		
Nuno	Martins			Hospital Professor Doutor Fernando Fonseca, Amadora, Portugal	Portugal		
Caroline	Martins Rego			Instituto de Infectologia Emílio Ribas, Sao Paulo, Brazil	Brazil		
Gennaro	Martucci			Istituto Mediterraneo per i Trapianti e Terapie ad Alta Specializzazione, Palermo, Italy	Italy		
Olga	Martynenko			Lugansk State Medical University - Department of Internal Medicine No2, Lugansk, Ukraine	Ukraine		
Eva Miranda	Marwali			National Cardiovascular Center Harapan Kita Jakarta Indonesia, Jakarta, Indonesia	Indonesia		
Marsilla	Marzukie			Tawau Hospital, Sabah, Malaysia	Malaysia		

*First Name and Middle Initial(s)	*Last Name	*Suffix (eg, Jr, III)	Academic Degrees	Institution	Location (city, state/province, country)	Role or Contribution, eg, chair, principal investigator	Group (if more than 1 Group listed in the byline) and/or Subgroup (eg, Steering Committee)
David	Maslove			Kingston Health Sciences Centre, Kingston, Canada	Canada		
Sabina	Mason			Tallaght University Hospital, Dublin, Ireland	Ireland		
Moshe	Matan			The Baruch Padeh Medical Center Poriya, Tiberias, Israel	Israel		
Henrique	Mateus Fernandes			Hospital Sirio-Libanes, Sao Paulo, Brazil	Brazil		
Mathieu	Mattei			Centre Hospitalier Régional et Universitaire de Nancy - Hôpitaux de Brabois, Nancy, France	France		
Laurence	Maulin			Centre Hospitalier du Pays d'Aix, Aix-en-Provence, France	France		
Javier	Maynar			Hospital Universitario de Alava, Araba, Spain	Spain		
Mayfong	Mayxay			Salavan Provincial Hospital, Salavan, Laos	Laos		
Thierry	Mazzoni			Centre Hospitalier de Cholet, Cholet, France	France		
Lisa	Mc Sweeney			Cork University Hospital, Cork, Ireland	Ireland		
Colin	McArthur			Waikato Hospital, Hamilton, New Zealand	New Zealand		
Naina	McCann			Clinical Services Department Fajara, Gambia	Gambia		
Peter	McCanny			Monash University, Melbourne, Australia	Australia		
Anne	McCarthy			The Ottawa Hospital, Ottawa, Canada	Canada		
Aine	McCarthy			Galway University Hospital, Galway, Ireland	Ireland		
Colin	McCloskey			UH Cleveland Hospital, Cleveland, United States of America	United States of America		
Rachael	McConnochie			Auckland City Hospital (DCCM 82), Auckland, New Zealand	New Zealand		
Sherry	McDermott			Lankenau Institute of Medical Research, Wynnewood, United States of America	United States of America		
Sarah E.	McDonald			Oxford University (ISARIC4C), Oxford, United Kingdom	United Kingdom		
Aine	McElroy			Mater Misericordiae University, Dublin, Ireland	Ireland		

*First Name and Middle Initial(s)	*Last Name	*Suffix (eg, Jr, III)	Academic Degrees	Institution	Location (city, state/province, country)	Role or Contribution, eg, chair, principal investigator	Group (if more than 1 Group listed in the byline) and/or Subgroup (eg, Steering Committee)
Samuel	McElwee			University of Alabama at Birmingham Hospital, Birmingham, United States of America	United States of America		
Natalie	McEvoy			Beaumont Hospital, Dublin, Ireland	Ireland		
Allison	McGeer			Mount Sinai Hospital, Toronto, Canada	Canada		
Kenneth A.	McLean			Oxford University (ISARIC4C), Oxford, United Kingdom	United Kingdom		
Paul	McNally			Children's Health Ireland, Dublin, Ireland	Ireland		
Bairbre	McNicholas			Galway University Hospital, Galway, Ireland	Ireland		
Edel	Meaney			St Vincents University Hospital, Dublin, Ireland	Ireland		
Cécile	Mear-Passard			Centre Hospitalier Universitaire de Nantes (Hôpital femme-enfant-adolescent), Nantes, France	France		
Maggie	Mechlin			University of Cincinnati, Cincinnati, United States of America	United States of America		
Omar	Mehkri			Cleveland Clinic, Ohio, Ohio, OH, United States of America	United States of America		
Ferruccio	Mele			University of Campania, Carseta, Italy	Italy		
Luis	Melo			Hospital Professor Doutor Fernando Fonseca, Amadora, Portugal	Portugal		
João João	Mendes			Hospital Professor Doutor Fernando Fonseca, Amadora, Portugal	Portugal		
Ogechukwu	Menkiti			St Christopher's Hospital for Children, Philadelphia, United States of America	United States of America		
Kusum	Menon			Children's Hospital of Eastern Ontario, Ottawa, Canada	Canada		
France	Mentré			Université de Paris, France	France		
Alexander J.	Mentzer			Wellcome Centre for Human Genetics, University of Oxford, Oxford, UK	United Kingdom		
Noémie	Mercier			INSERM, Paris, France	France		
Emmanuelle	Mercier			Centre Hospitalier Régional et Universitaire de Tours, Tours, France	France		

*First Name and Middle Initial(s)	*Last Name	*Suffix (eg, Jr, III)	Academic Degrees	Institution	Location (city, state/province, country)	Role or Contribution, eg, chair, principal investigator	Group (if more than 1 Group listed in the byline) and/or Subgroup (eg, Steering Committee)
Antoine	Merckx			Centre Hospitalier de Cahors, Cahors, France	France		
Mayka	Mergeay-Fabre			Centre Hospitalier Andrée Rosemon, Cayenne, French Guiana	French Guiana		
Blake	Mergler			Perelman School of Medicine at the University of Pennsylvania, Philadelphia, United States of America	United States of America		
António	Mesquita			Hospital Curry Cabral - Intensive Care Unit - UCIP7, Lisbon, Portugal	Portugal		
Roberta	Meta			St Bernard's Hospital, Gibraltar, Gibraltar	Gibraltar		
Osama	Metwally			Galway University Hospital, Galway, Ireland	Ireland		
Agnès	Meybeck			Centre Hospitalier de Tourcoing, Tourcoing, France	France		
Dan	Meyer			Baylor Scott & White Health, Temple, United States of America	United States of America		
Alison M.	Meynert			MRC Human Genetics Unit, MRC Institute of Genetics and Molecular Medicine, University of Edinburgh, Edinburgh, UK	United Kingdom		
Vanina	Meysonnier			Groupe Hospitalier Diaconesses Croix Saint-Simon, Paris, France	France		
Mehdi	Mezidi			Hôpital Lyon Sud - HCL, Lyon, France	France		
Céline	Michelanglei			Centre Hospitalier Universitaire de Nice (Hôpital Archet), Nice, France	France		
Isabelle	Michelet			Centre Hospitalier Universitaire Rouen (Hôpital Charles Nicolle), Rouen, France	France		
Efstathia	Mihelis			Northwell Health, New York, United States of America	United States of America		
Vladislav	Mihnovit			Tartu University Hospital, Tartu, Estonia	Estonia		
Jennene	Miller			Monash University, Melbourne, Australia	Australia		
Hugo	Miranda-Maldonado			Universidad de Las Américas, Quito, Ecuador	Ecuador		
Nor Arisah	Misnan			Sungai Buloh Hospital, Selangor, Malaysia	Malaysia		
Tahira Jamal	Mohamed			Kuala Lumpur Hospital, WPKL, Malaysia	Malaysia		
Nik Nur Eliza	Mohamed			Kuala Lumpur Hospital, WPKL, Malaysia	Malaysia		

*First Name and Middle Initial(s)	*Last Name	*Suffix (eg, Jr, III)	Academic Degrees	Institution	Location (city, state/province, country)	Role or Contribution, eg, chair, principal investigator	Group (if more than 1 Group listed in the byline) and/or Subgroup (eg, Steering Committee)
Asma	Moin			William Osler Health Sciences System - Etobicoke General Hospital, Toronto, Canada	Canada		
Elena	Molinos			Comissão de Ética - Unidade Local de Saúde de Matosinhos, Porto, Portugal	Portugal		
Brenda	Molloy			St Vincents University Hospital, Dublin, Ireland	Ireland		
Mary	Mone			University of Utah, Salt Lake City, United States of America	United States of America		
Agostinho	Monteiro			Hospital Curry Cabral - Intensive Care Unit - UCIP7, Lisbon, Portugal	Portugal		
Claudia	Montes			Clinica Valle de Lilli, Valle del Cauca, Colombia	Colombia		
Giorgia	Montrucchio			Ospedale Molinette, Torino, Italy	Italy		
Shona C.	Moore			Institute of Infection, Veterinary and Ecological Sciences, University of Liverpool, Liverpool, UK	United Kingdom		
Sarah	Moore			ISARIC Global Support Centre, Oxford, United Kingdom	United Kingdom		
Lina	Morales Cely			Clinica Universidad de La Sabana, Chia, Colombia	Colombia		
Lucia	Moro			Ospedale Sacro Cuore Don Calabria, Negrar Di Valpolicella, Italy	Italy		
Ben	Morton			Liverpool School of Tropical Medicine, Liverpool, UK	Malawi		
Catherine	Motherway			University Hospital - Limerick, Limerick, Ireland	Ireland		
Ana	Motos			Hospital Clinic, Barcelona, Spain	Spain		
Hugo	Mouquet			INSERM, Paris, France	France		
Clara	Mouton Perrot			Centre Hospitalier Henri Duffaut, Avignon, France	France		
Julien	Moyet			Centre Hospitalier Universitaire Amiens-Picardie, Amiens, France	France		
Caroline	Mudara			National Institute for Communicable Diseases, Johannesburg, South Africa	South Africa		

*First Name and Middle Initial(s)	*Last Name	*Suffix (eg, Jr, III)	Academic Degrees	Institution	Location (city, state/province, country)	Role or Contribution, eg, chair, principal investigator	Group (if more than 1 Group listed in the byline) and/or Subgroup (eg, Steering Committee)
Ng Yong	Muh			Kuala Lumpur Hospital, WPKL, Malaysia	Malaysia		
Dzawani	Muhamad			Tengku Ampuan Afzan Hospital, Pahang, Malaysia	Malaysia		
Jimmy	Mullaert			INSERM, Paris, France	France		
Fredrik	Müller			Oslo University Hospital, Oslo, Norway	Norway		
Karl Erik	Müller			Drammen Hospital, Drammen, Norway	Norway		
Daniel	Munblit			Care for Long Term Conditions Division, Florence Nightingale Faculty of Nursing, Midwifery and Palliative Care, King's College London, London, United Kingdom	Russia		
Laveena	Munshi			Sinai Health Systems, Toronto, Canada	Canada		
Aisling	Murphy			University Hospital - Waterford, Waterford, Ireland	Ireland		
Patrick	Murray			St Vincents University Hospital, Dublin, Ireland	Ireland		
Marlène	Murris			Centre Hospitalier Universitaire Toulouse (Larrey), Toulouse, France	France		
Srinivas	Murthy			Faculty of Medicine, University of British Columbia, Vancouver, Canada	United Kingdom		
Himed	Musaab			Wexford General Hospital, Wexford, Ireland	Ireland		
Himasha	Muvindi			CCA Network	Unknown		
Dimitra Melia	Myrodia			Sotiria General Hospital, Athens, Greece	Greece		
Behzad	Nadjm			Clinical Services Department Fajara, Gambia	Gambia		
Dave	Nagpal			London Health Sciences Centre, London, Canada	Canada		
Alex	Nagrebetsky			Baystate MC, Springfield, United States of America	United States of America		
Mangala	Narasimhan			Northwell Health, New York, United States of America	United States of America		
Nageswaran	Narayanan			Our lady of Lourdes Drogheda, Drogheda, Ireland	Ireland		
Alasdair	Nazerali-Maitland			University Hospital Northern British Columbia, Prince George, Canada	Canada		

*First Name and Middle Initial(s)	*Last Name	*Suffix (eg, Jr, III)	Academic Degrees	Institution	Location (city, state/province, country)	Role or Contribution, eg, chair, principal investigator	Group (if more than 1 Group listed in the byline) and/or Subgroup (eg, Steering Committee)
Nadège	Neant			INSERM, Paris, France	France		
Holger	Neb			Uniklinik University Hospital, Frankfurt, Germany	Germany		
Nikita	Nekliudov			Sechenov University, Moscow, Russia	Russia		
Matthew	Nelder			Memorial University, St. John's, Newfoundland, St John's, Canada	Canada		
Erni	Nelwan			Pratama Rada Bolo Hospital, Karitas Hospital and Waikabubak Hospital, Sumba, Indonesia	Indonesia		
Raul	Neto			Centro Hospitalar Vila Nova de Gaia/Espinho, Espinho, Portugal	Portugal		
Emily	Neumann			Medical College of Wisconsin, Wisconsin, United States of America	United States of America		
Pauline Yeung	Ng			Queen Mary Hospital, Pok Fu Lam, China	China		
Wing Yiu	Ng			Queen Elizabeth Hospital, Yau Ma Tei, China	China		
Anthony	Nghi			Centre Hospitalier Techer, Calais, France	France		
Duc	Nguyen			Hôpital Pellegrin, Bordeaux, France	France		
Orna	Ni Choileain			Beaumont Hospital, Dublin, Ireland	Ireland		
Niamh	Ni Leathlobhair			Galway University Hospital, Galway, Ireland	Ireland		
Alistair D	Nichol			Irish Critical Care Critical Clinical Trials Network, Dublin, Ireland, UK	Ireland		
Prompak	Nitayavardhana			Siriraj Piyamaharajkarun Hospital (SiPH), Bangkok, Thailand	Thailand		
Stephanie	Nonas			Oregon Health & Science University, Portland, United States of America	United States of America		
Nurul Amani Mohd	Noordin			Melaka Hospital, Melaka, Malaysia	Malaysia		
Nurul Faten Izzati	Norharizam			Sungai Buloh Hospital, Selangor, Malaysia	Malaysia		
Alessandra	Notari			Hospital Escola da Universidade Federal de Pelotas, Pelotas, Brazil	Brazil		
Mahdad	Noursadeghi			Division of Infection and Immunity, University College London, UK	United Kingdom		
Adam	Nowinski			Institute of TB and Lung Diseases, Warsaw, Poland	Poland		

*First Name and Middle Initial(s)	*Last Name	*Suffix (eg, Jr, III)	Academic Degrees	Institution	Location (city, state/province, country)	Role or Contribution, eg, chair, principal investigator	Group (if more than 1 Group listed in the byline) and/or Subgroup (eg, Steering Committee)
Saad	Nseir			Centre Hospitalier Universitaire de Lille, Lille, France	France		
Nurnaningsih	Nurnaningsih			Dr Sardjito Government Hospital (Paediatric), Yogyakarta, Indonesia	Indonesia		
Dwi Utomo	Nusantara			RSUD Pasar Minggu, South Jakarta, Indonesia	Indonesia		
Elsa	Nyamankolly			Centre Hospitalier de Dax - Côte d'Argent, Dax, France	France		
Anders Benteson	Nygaard			The Norwegian Corona Cohort, Oslo, Norway	Norway		
Fionnuala	O Brien			Cork University Hospital, Cork, Ireland	Ireland		
Annmarie	O Callaghan			Cork University Hospital, Cork, Ireland	Ireland		
Giovanna	Occhipinti			Istituto Mediterraneo per i Trapianti e Terapie ad Alta Specializzazione, Palermo, Italy	Italy		
Derbrenn	OConnor			Bon Secours Hospital, Cork, Ireland	Ireland		
Max	O'Donnell			Columbia University, New York, United States of America	United States of America		
Tawnya	Ogston			Legacy Emanuel Medical Center, Portland, United States of America	United States of America		
Takayuki	Ogura			Saiseikai Utsunomiya Hospital, Tochigi, Japan	Japan		
Tak-Hyuk	Oh			Kyung Pook National University Chilgok Hospital, Daegu, South Korea	South Korea		
Sophie	O'Halloran			Connolly Hospital Blanchardstown, Dublin, Ireland	Ireland		
Katie	O'Hearn			Children's Hospital of Eastern Ontario, Ottawa, Canada	Canada		
Agnieszka	Oldakowska			Department of Children's Infectious Diseases, Warsaw, Poland	Poland		
João	Oliveira			Hospital de São José -U.U.M., Lisbon, Portugal	Portugal		
Piero L.	Olliaro			ISARIC, Pandemic Sciences Institute, University of Oxford, Oxford, UK	United Kingdom		
David S. Y.	Ong			Department of Medical Microbiology and Infection Control, Franciscus Gasthuis & Vlietland, Rotterdam, Netherlands	Netherlands		

*First Name and Middle Initial(s)	*Last Name	*Suffix (eg, Jr, III)	Academic Degrees	Institution	Location (city, state/province, country)	Role or Contribution, eg, chair, principal investigator	Group (if more than 1 Group listed in the byline) and/or Subgroup (eg, Steering Committee)
Jee Yan	Ong			Sarawak General Hospital, Sarawak, Malaysia	Malaysia		
Wilna	Oosthuyzen			Oxford University (ISARIC4C), Oxford, United Kingdom	United Kingdom		
Anne	Opavsky			Joseph Brant Hospital, Burlington, Canada	Canada		
Peter	Openshaw			National Heart and Lung Institute, Imperial College London, London, UK	United Kingdom		
Claudia Milena	Orozco-Chamorro			Universidad del Cauca, Cauca, Colombia	Colombia		
Jamel	Ortoleva			Tufts Medical Centre, Boston, United States of America	United States of America		
Javier	Osatnik			Hospital Aleman, Buenos Aires, Argentina	Argentina		
Linda	O'Shea			Wexford General Hospital, Wexford, Ireland	Ireland		
Miriam	O'Sullivan			Sligo University Hospital (Saolta), Sligo, Ireland	Ireland		
Siti Zubaidah	Othman			Kuala Lumpur Hospital, WPKL, Malaysia	Malaysia		
Paul	Otiku			Bar-Ilan University, Ramat Gan, Israel	Israel		
Nadia	Ouamara			Centre Hospitalier Régional Metz-Thionville, Metz, France	France		
Rachida	Ouissa			Centre Hospitalier Universitaire de Guadeloupe, Pointe-à-Pitre, Guadeloupe	Guadeloupe		
Eric	Oziol			Centre Hospitalier de Béziers, Béziers, France	France		
Maïder	Pagadoy			Centre Hospitalier Universitaire de Besançon, Besançon, France	France		
Justine	Pages			INSERM, Paris, France	France		
Massimo	Palmarini			MRC-University of Glasgow Centre for Virus Research, 464 Bearsden Road, Glasgow, UK	United Kingdom		
Giovanna	Panarello			Istituto Mediterraneo per i Trapianti e Terapie ad Alta Specializzazione, Palermo, Italy	Italy		
Prasan Kumar	Panda			All India Institute of Medical Sciences (AIIMS), Rishikesh, India	India		
Lai Hui	Pang			Sungai Buloh Hospital, Selangor, Malaysia	Malaysia		
Mauro	Panigada			Fondazione IRCCS Ca, Milan, Italy	Italy		

*First Name and Middle Initial(s)	*Last Name	*Suffix (eg, Jr, III)	Academic Degrees	Institution	Location (city, state/province, country)	Role or Contribution, eg, chair, principal investigator	Group (if more than 1 Group listed in the byline) and/or Subgroup (eg, Steering Committee)
Nathalie	Pansu			Centre Hospitalier Universitaire de Montpellier, Montpellier, France	France		
Aurélie	Papadopoulos			INSERM, Paris, France	France		
Rachael	Parke			Auckland City Hospital (CVICU), Auckland, New Zealand	New Zealand		
Melissa	Parker			McMaster University, Hamilton, Canada	Canada		
Jérémie	Pasquier			Centre Hospitalier Universitaire de Martinique, Fort-de-France, Saint Martin (French)	Saint Martin (French)		
Bruno	Pastene			Hôpital de la Timone, Marseille, France	France		
Fabian	Patauner			University of Campania, Carseta, Italy	Italy		
Drashti	Patel			Long COVID India - Terna Specialty Hospital and Research Centre, Mumbai, India	India		
Mohan Dass	Pathmanathan			National Institutes of Health (NIH), Ministry of Health Malaysia, Setia Alam, Malaysia	Malaysia		
Luís	Patrão			Centro Hospitalar de Tondela-Viseu, Viseu, Portugal	Portugal		
Patricia	Patricio			Hospital Beatriz Ângelo, Loures, Portugal	Portugal		
Lisa	Patterson			Niagara Health, Niagara, Canada	Canada		
Mical	Paul			Rambam Hospital, Haifa, Israel	Israel		
Christelle	Paul			INSERM, Paris, France	France		
Jorge	Paulos			Hospital Curry Cabral - Intensive Care Unit - UCIP7, Lisbon, Portugal	Portugal		
William A.	Paxton			Institute of Infection, Veterinary and Ecological Sciences, University of Liverpool, Liverpool, UK	United Kingdom		
Jean-François	Payen			Centre Hospitalier Universitaire Grenoble-Alpes, Grenoble, France	France		
Sandra L	Peake			Monash University, Melbourne, Australia	Australia		
Kalaiarasu	Peariasamy			National Institutes of Health (NIH), Ministry of Health Malaysia, Setia Alam, Malaysia	Malaysia		
Miguel	Pedrera Jiménez			Hospital 12 de Octubre, Madrid, Spain	Spain		

*First Name and Middle Initial(s)	*Last Name	*Suffix (eg, Jr, III)	Academic Degrees	Institution	Location (city, state/province, country)	Role or Contribution, eg, chair, principal investigator	Group (if more than 1 Group listed in the byline) and/or Subgroup (eg, Steering Committee)
Giles J.	Peek			University of Florida, Gainesville, United States of America	United States of America		
Florent	Peelman			Centre Hospitalier de Périgueux, Périgueux, France	France		
Nathan	Peiffer-Smadja			INSERM, Paris, France	France		
Vincent	Peigne			Centre Hospitalier Métropole Savoie, Chambéry, France	France		
Mare	Pejkovska			Mount Sinai Hospital, Toronto, Canada	Canada		
Paolo	Pelosi			San Martino Hospital, Genoa, Italy	Italy		
Ithan D.	Peltan			US NHLBI PETAL Network, Boston, United States of America	United States of America		
Rui	Pereira			Hospital Curry Cabral - Intensive Care Unit - UCIP7, Lisbon, Portugal	Portugal		
Daniel	Perez			Universidad del Cauca, Cauca, Colombia	Colombia		
Thomas	Perpoint			Centre Hospitalier Universitaire de Lyon - HCL, Lyon, France	France		
Antonio	Pesenti			Fondazione IRCCS Ca, Milan, Italy	Italy		
Vincent	Pestre			Centre Hospitalier Henri Duffaut, Avignon, France	France		
Lenka	Petrou			University Hospital Ostrava, Ostrava-Poruba, Czechia	Czechia		
Michele	Petrovic			Humber River Hospital, Toronto, Canada	Canada		
Ventzislava	Petrov-Sanchez			INSERM, Paris, France	France		
Frank Olav	Pettersen			Oslo University Hospital, Oslo, Norway	Norway		
Gilles	Peytavin			INSERM, Paris, France	France		
Ooyanong	Phonemixay			Salavan Provincial Hospital, Salavan, Laos	Laos		
Soulichanya	Phoutthavong			Attapeu Provincial Hospital, Laos	Laos		
Michael	Piagnerelli			Civil Hospital Marie Curie, Charleroi, Belgium	Belgium		
Walter	Picard			Centre Hospitalier de Pau, Pau, France	France		
Olivier	Picone			INSERM, Paris, France	France		
Maria de	Piero			Maastricht University Medical Centre, Maastricht, Netherlands	Netherlands		

*First Name and Middle Initial(s)	*Last Name	*Suffix (eg, Jr, III)	Academic Degrees	Institution	Location (city, state/province, country)	Role or Contribution, eg, chair, principal investigator	Group (if more than 1 Group listed in the byline) and/or Subgroup (eg, Steering Committee)
Djura	Piersma			Medisch Spectrum Twente, Zutphen, Netherlands	Netherlands		
Carlos	Pimentel			Hospital Curry Cabral - Intensive Care Unit - UCIP7, Lisbon, Portugal	Portugal		
Raquel	Pinto			Hospital de Curry Cabral - Infectious Diseases, Lisbon, Portugal	Portugal		
Catarina	Pires			Hospital Curry Cabral - Intensive Care Unit - UCIP7, Lisbon, Portugal	Portugal		
Lionel	Piroth			Centre Hospitalier Universitaire Mitterrand Dijon-Bourgogne, Dijon, France	France		
Ayodhia	Pitaloka			Murni Teguh Memorial Hospital and Bunda Thamrin Hospital, North Sumatera, Indonesia	Indonesia		
Chiara	Piubelli			Department of Infectious, Tropical Diseases and Microbiology, IRCCS Sacro Cuore Don Calabria Hospital, Negrar, Italy	Italy		
Riinu	Pius			Oxford University (ISARIC4C), Oxford, United Kingdom	United Kingdom		
Simone	Piva			University of Brescia, Brescia, Italy	Italy		
Laurent	Plantier			Centre Hospitalier Régional et Universitaire de Tours, Tours, France	France		
Hon Shen	Png			Kuala Lumpur Hospital, WPKL, Malaysia	Malaysia		
Julien	Poissy			Centre Hospitalier Universitaire de Lille, Lille, France	France		
Ryadh	Pokeerbux			Hôpital Albert Calmette, Lille, France	France		
Maria	Pokorska-Spiewak			Department of Children's Infectious Diseases, Warsaw, Poland	Poland		
Sergio	Poli			Mount Sinai Medical Center, Miami, FL, United States of America	United States of America		
Georgios	Pollakis			Institute of Infection, Veterinary and Ecological Sciences, University of Liverpool, Liverpool, UK	United Kingdom		
Diane	Ponscarme			Hôpital Saint-Louis AP-HP, Paris, France	France		

*First Name and Middle Initial(s)	*Last Name	*Suffix (eg, Jr, III)	Academic Degrees	Institution	Location (city, state/province, country)	Role or Contribution, eg, chair, principal investigator	Group (if more than 1 Group listed in the byline) and/or Subgroup (eg, Steering Committee)
Jolanta	Popielska			Department of Children's Infectious Diseases, Warsaw, Poland	Poland		
Diego Bastos	Porto			Sao Camilo Cura D'ars, Fortaleza, Brazil	Brazil		
Andra-Maris	Post			North Estonia Medical Centre, Tallin, Estonia	Estonia		
Douwe F.	Postma			University Medical Center Groningen, Groningen, Netherlands	Netherlands		
Pedro	Povoa			Hospital São Francisco Xavier, Lisbon, Portugal	Portugal		
Diana	Póvoas			Hospital de Curry Cabral - Infectious Diseases, Lisbon, Portugal	Portugal		
Jeff	Powis			Michael Garron Hospital, Toronto, Canada	Canada		
Sofia	Prapa			Sotiria General Hospital, Athens, Greece	Greece		
Viladeth	Praphasiri			Xieng Khouang Provincial Hospital, Phonsavan, Laos	Laos		
Sébastien	Preau			Centre Hospitalier Universitaire de Lille, Lille, France	France		
Christian	Prebensen			Akershus University Hospital, Nordbyhagen, Norway	Norway		
Jean-Charles	Preiser			CUB-Hopital Erasme, Anderlecht, Belgium	Belgium		
Anton	Prinssen			Canisius Wilhelmina Ziekenhuis, Nijmenjen, Netherlands	Netherlands		
Mark G.	Pritchard			ISARIC, Pandemic Sciences Institute, University of Oxford, UK	United Kingdom		
Gamage Dona Dilanthi	Priyadarshani			CCA Network	Unknown		
Lucia	Proença			Hospital de São José -U.U.M., Lisbon, Portugal	Portugal		
Sravya	Pudota			University Hospital - Limerick, Limerick, Ireland	Ireland		
Bambang	Pujo Semedi			RSUD Dr. Soetomo, Surabaya, Indonesia	Indonesia		
Luisa	Quesada			Mount Sinai Medical Center, Miami, FL, United States of America	United States of America		
Vilmaris	Quinones-Cardona			St Christopher's Hospital for Children, Philadelphia, United States of America	United States of America		

*First Name and Middle Initial(s)	*Last Name	*Suffix (eg, Jr, III)	Academic Degrees	Institution	Location (city, state/province, country)	Role or Contribution, eg, chair, principal investigator	Group (if more than 1 Group listed in the byline) and/or Subgroup (eg, Steering Committee)
Víctor	Quirós González			Hospital 12 de Octubre, Madrid, Spain	Spain		
Else	Quist-Paulsen			Oslo University Hospital, Oslo, Norway	Norway		
Mohammed	Quraishi			Presbyterian Hospital Services, Alberquerque, United States of America	United States of America		
Fadi	Qutishat			Galway University Hospital, Galway, Ireland	Ireland		
Maia	Rabaa			Pratama Rada Bolo Hospital, Karitas Hospital and Waikabubak Hospital, Sumba, Indonesia	Indonesia		
Christian	Rabaud			Centre Hospitalier Régional et Universitaire de Nancy - Hôpitaux de Brabois, Nancy, France	France		
Aldo	Rafael			Baylor University Medical Centre, Dallas, United States of America	United States of America		
Marie	Rafiq			Hôpital Purpan, Toulouse, France	France		
Mutia	Rahardjani			Murni Teguh Memorial Hospital and Bunda Thamrin Hospital, North Sumatera, Indonesia	Indonesia		
Rozanah Abd	Rahman			Permai Hospital, Johor, Malaysia	Malaysia		
Ahmad Kashfi Haji Ab	Rahman			Sultanah Nur Zahirah Hospital, Terengganu, Malaysia	Malaysia		
Fernando	Rainieri			Hospital de Clínicas, Buenos Aires, Argentina	Argentina		
Giri Shan	Rajahram			Tawau Hospital, Sabah, Malaysia	Malaysia		
Nagarajan	Ramakrishnan			Apollo Hospitals Chennai, Chennai, Tamil Nadu, India	India		
José	Ramalho			Hospital Curry Cabral - Intensive Care Unit - UCIP7, Lisbon, Portugal	Portugal		
Ahmad Afiq	Ramli			Sungai Buloh Hospital, Selangor, Malaysia	Malaysia		
Blandine	Rammaert			Centre Hospitalier Universitaire de Poitiers, Poitiers, France	France		
Grazielle Viana	Ramos			National Institute of Infectious Disease Evandro Chagas, Oswaldo Cruz Foundation (INI-FIOCRUZ), Ministry of Health, and D'Or Institute of Research and Education (IDOR), Rio de Janeiro, Gaspar Viana Pavilion, Brazil	Brazil		

*First Name and Middle Initial(s)	*Last Name	*Suffix (eg, Jr, III)	Academic Degrees	Institution	Location (city, state/province, country)	Role or Contribution, eg, chair, principal investigator	Group (if more than 1 Group listed in the byline) and/or Subgroup (eg, Steering Committee)
Rajavardhan	Rangappa			Manipal Hospital Whitefield, Bangalore, India	India		
Ritika	Ranjan			Galway University Hospital, Galway, Ireland	Ireland		
Christophe	Rapp			Hôpital Américain de Paris, Neuilly-sur-Seine, France	France		
Aasiyah	Rashan			Network for Improving Critical care Systems and Training, Colombo, Sri Lanka	Unknown		
Thalha	Rashan			CCA Network	Unknown		
Menaldi	Rasmin			Persahabatan Hospital, Jakarta, Indonesia	Indonesia		
Indrek	Rätsep			Clinic of Anesthesiology and Intensive Care, North Estonia Medical Centre, Tallinn, Estonia	Estonia		
Cornelius	Rau			University Children's Hospital, University Medical Center Hamburg-Eppendorf, Hamburg, Germany	Germany		
Tharmini	Ravi			Sungai Buloh Hospital, Selangor, Malaysia	Malaysia		
Andre	Real			Hospital de Abrantes - ICU, Abrantes, Portugal	Portugal		
Stanislas	Rebaudet			Hôpital Européen Marseille, Marseille, France	France		
Sarah	Redl			Centre Hospitalier Agen-Nérac, Agen, France	France		
Brenda	Reeve			Brantford General Hospital, Brantford, Canada	Canada		
Dag Henrik	Reikvam			Oslo University Hospital, Oslo, Norway	Norway		
Renato	Reis			Hospital Santa Maria, Centro Hospitalar Universitário Lisboa Norte, Amadora, Portugal	Portugal		
Jordi	Rello			Vall d'Hebron Institute of Research, Barcelona, Spain	Spain		
Jonathan	Remppis			University Hospital of Tubingen, Tubingen, Germany	Germany		
Martine	Remy			Centre Hospitalier Universitaire de Lille, Lille, France	France		
Hongru	Ren			University of Manitoba, Manitoba, Canada	Canada		
Hanna	Renk			University Hospital of Tubingen, Tubingen, Germany	Germany		

*First Name and Middle Initial(s)	*Last Name	*Suffix (eg, Jr, III)	Academic Degrees	Institution	Location (city, state/province, country)	Role or Contribution, eg, chair, principal investigator	Group (if more than 1 Group listed in the byline) and/or Subgroup (eg, Steering Committee)
Anne-Sophie	Resseguier			Centre Hospitalier Emile Roux, Le Puy-en-Velay, France	France		
Matthieu	Revest			Centre Hospitalier Universitaire Rennes (Hôpital Pontchaillou), Rennes, France	France		
Oleksa	Rewa			University of Alberta Adult ICU, Edmonton, Canada	Canada		
Luis Felipe	Reyes			Universidad de La Sabana, Chia, Colombia	Colombia		
Maria Ines	Ribeiro			Hospital Espírito Santo de Évora, Évora, Portugal	Portugal		
Antonia	Ricchiuto			Università Cattolica del Sacro Cuore, Rome, Italy	Italy		
David	Richardson			William Osler Health Sciences System - Etobicoke General Hospital, Toronto, Canada	Canada		
Denise	Richardson			University of Cincinnati, Cincinnati, United States of America	United States of America		
Laurent	Richier			Centre Hospitalier Intercommunal Villeneuve-Saint-Georges, Villeneuve-Saint-Georges, France	France		
Siti Nurul Atikah Ahmad	Ridzuan			Sultanah Nur Zahirah Hospital, Terengganu, Malaysia	Malaysia		
Ana L	Rios			Centro Hospitalar Vila Nova de Gaia/Espinho, Espinho, Portugal	Portugal		
Asgar	Rishu			Sunnybrook Health Sciences Centre, Toronto, Canada	Canada		
Patrick	Rispal			Centre Hospitalier Agen-Nérac, Agen, France	France		
Karine	Risso			Centre Hospitalier Universitaire de Nice (Hôpital Archet), Nice, France	France		
Maria Angelica	Rivera Nuñez			La Paz Hospital, Madrid, Spain	Spain		
Chiara	Robba			San Martino Hospital, Genoa, Italy	Italy		
André	Roberto			Hospital de São José -U.U.M., Lisbon, Portugal	Portugal		
Charles	Roberts			Clinical Services Department Fajara, Gambia	Gambia		
David L.	Robertson			MRC-University of Glasgow Centre for Virus Research, 464 Bearsden Road, Glasgow, UK	United Kingdom		

*First Name and Middle Initial(s)	*Last Name	*Suffix (eg, Jr, III)	Academic Degrees	Institution	Location (city, state/province, country)	Role or Contribution, eg, chair, principal investigator	Group (if more than 1 Group listed in the byline) and/or Subgroup (eg, Steering Committee)
Olivier	Robineau			Centre Hospitalier de Tourcoing, Tourcoing, France	France		
Anna	Roca			Clinical Services Department Fajara, Gambia	Gambia		
Ferran	Roche-Campo			Hospital Verge de la Cinta, Tortosa, Spain	Spain		
Paola	Rodari			Ospedale Sacro Cuore Don Calabria, Negrar Di Valpolicella, Italy	Italy		
Simão	Rodeia			Hospital de São José -U.U.M., Lisbon, Portugal	Portugal		
Bernhard	Roessler			Medical University of Vienna, Vienna, Austria	Austria		
Claire	Roger			CHU Carémeau, Nimes, France	France		
Pierre-Marie	Roger			Centre Hospitalier Universitaire de Guadeloupe, Pointe-à-Pitre, Guadeloupe	Guadeloupe		
Emmanuel	Roilides			Hippokration Hospital, Thessaloniki, Greece	Greece		
Amanda	Rojek			Royal Melbourne Hospital, Melbourne, Australia	United Kingdom		
Roberto	Roncon-Albuquerque	Jr		São João Hospital Centre, Porto, Portugal	Portugal		
Mélanie	Roriz			Centre Hospitalier Agen-Nérac, Agen, France	France		
Manuel	Rosa-Calatrava			INSERM, Paris, France	France		
Michael	Rose			McLeod Healthcare System, Florence, United States of America	United States of America		
Dorothea	Rosenberger			University of Utah, Salt Lake City, United States of America	United States of America		
Andrea	Rossanese			Ospedale Sacro Cuore Don Calabria, Negrar Di Valpolicella, Italy	Italy		
Matteo	Rossetti			Istituto Mediterraneo per i Trapianti e Terapie ad Alta Specializzazione, Palermo, Italy	Italy		
Patrick	Rossignol			INSERM, Paris, France	France		
Carine	Roy			INSERM, Paris, France	France		
Benoît	Roze			Centre Hospitalier de Saintonge, Saintes, France	France		
Desy	Rusmawatiningtyas			Dr Sardjito Government Hospital (Paediatric), Yogyakarta, Indonesia	Indonesia		

*First Name and Middle Initial(s)	*Last Name	*Suffix (eg, Jr, III)	Academic Degrees	Institution	Location (city, state/province, country)	Role or Contribution, eg, chair, principal investigator	Group (if more than 1 Group listed in the byline) and/or Subgroup (eg, Steering Committee)
Clark D.	Russell			Centre for Inflammation Research, The Queen's Medical Research Institute, University of Edinburgh, 47 Little France Crescent, Edinburgh, UK	United Kingdom		
Maeve	Ryan			Galway University Hospital, Galway, Ireland	Ireland		
Steffi	Ryckaert			AZ Maria Middelares, Gent, Belgium	Belgium		
Aleksander	Rygh Holten			Oslo University Hospital, Oslo, Norway	Norway		
Isabela	Saba			Mount Sinai Medical Center, Miami, FL, United States of America	United States of America		
Musharaf	Sadat			King Abdulaziz Medical City, Riyadh, Saudi Arabia	Saudi Arabia		
Valla	Sahraei			Lions Gate Hospital, Vancouver, Canada	Canada		
Pranya	Sakiyalak			Siriraj Piyamaharajkarun Hospital (SiPH), Bangkok, Thailand	Thailand		
Leonardo	Salazar			Fundación Cardiovascular de Colombia, Floridablanca, Colombia	Colombia		
Gabriele	Sales			Ospedale Molinette, Torino, Italy	Italy		
Charlotte	Salmon Gandonniere			Centre Hospitalier Régional et Universitaire de Tours, Tours, France	France		
Hélène	Salvator			Hôpital Foch, Suresnes, France	France		
Emely	Sanchez			University of Nebraska Medical Centre	United States of America		
Olivier	Sanchez			Hôpital Européen Georges-Pompidou AP-HP, Paris, France	France		
Kizy	Sanchez de Oliveira			Hospital Sirio-Libanes, Sao Paulo, Brazil	Brazil		
Angel	Sanchez-Miralles			Hospital Universitari Sant Joan D'Alacant, Alicante, Spain	Spain		
Vanessa	Sancho-Shimizu			Department of Pediatrics and Virology, St Mary's Medical School Bldg, Imperial College London, London, UK	United Kingdom		
Gyan	Sandhu			Unity Health Toronto, Toronto, Canada	Canada		
Zulfiqar	Sandhu			Wexford General Hospital, Wexford, Ireland	Ireland		

*First Name and Middle Initial(s)	*Last Name	*Suffix (eg, Jr, III)	Academic Degrees	Institution	Location (city, state/province, country)	Role or Contribution, eg, chair, principal investigator	Group (if more than 1 Group listed in the byline) and/or Subgroup (eg, Steering Committee)
Pierre-François	Sandrine			Centre Hospitalier Universitaire de Martinique, Fort-de-France, Saint Martin (French)	Saint Martin (French)		
Marlene	Santos			Unity Health Toronto, Toronto, Canada	Canada		
Shirley	Sarfo-Mensah			The Ottawa Hospital, Ottawa, Canada	Canada		
Bruno	Sarmento Banheiro			Centro Hospitalar Universitário do Algarve, Portimão, Portugal	Portugal		
Iam Claire E.	Sarmiento			Northwell Health, New York, United States of America	United States of America		
Benjamine	Sarton			Hôpital Purpan, Toulouse, France	France		
Ankana	Satya			Long COVID India - Terna Specialty Hospital and Research Centre, Mumbai, India	India		
Sree	Satyapriya			Ohio State University, Columbus, United States of America	United States of America		
Rumaisah	Satyawati			RSPI Prof Dr Sulianti Saroso, Jakarta, Indonesia	Indonesia		
Egle	Saviciute			Oxford University (ISARIC4C), Oxford, United Kingdom	United Kingdom		
Parthena	Savvidou			Hippokration Hospital, Thessaloniki, Greece	Greece		
Yen Tsen	Saw			Queen Elizabeth Hospital, Sabah, Malaysia	Malaysia		
Justin	Schaffer			The Heart Hospital Baylor Plano, Plano, United States of America	United States of America		
Tjard	Schermer			Gelre Hospitals, Zutphen, Netherlands	Netherlands		
Arnaud	Scherpereel			Hôpital Albert Calmette, Lille, France	France		
Marion	Schneider			INSERM, Paris, France	France		
Michael	Schwameis			Medical University of Vienna, Vienna, Austria	Austria		
Gary	Schwartz			Baylor AllSaints Medical Centre, Fort Worth, United States of America	United States of America		
Janet T.	Scott			MRC-University of Glasgow Centre for Virus Research, 464 Bearsden Road, Glasgow, UK	United Kingdom		
James	Scott-Brown			Oxford University (ISARIC4C), Oxford, United Kingdom	United Kingdom		

*First Name and Middle Initial(s)	*Last Name	*Suffix (eg, Jr, III)	Academic Degrees	Institution	Location (city, state/province, country)	Role or Contribution, eg, chair, principal investigator	Group (if more than 1 Group listed in the byline) and/or Subgroup (eg, Steering Committee)
Nicholas	Sedillot			Centre Hospitalier de Bourg-en-Bresse, Bourg-en-Bresse, France	France		
Tamara	Seitz			Sozialmedizinisches Zentrum Sud, Vienna, Austria	Austria		
Mageswari	Selvarajoo			Sungai Buloh Hospital, Selangor, Malaysia	Malaysia		
Malcolm G.	Semple			Institute of Infection, Veterinary and Ecological Sciences, Faculty of Health and Life Sciences, University of Liverpool, Liverpool, UK	United Kingdom		
Rasidah Bt	Senian			Kuala Lumpur Hospital, WPKL, Malaysia	Malaysia		
Eric	Senneville			Centre Hospitalier de Tourcoing, Tourcoing, France	France		
Claudia	Sepulveda			Instituto Nacional Del Tórax, Santiago, Chile	Chile		
Filipa	Sequeira			Centro Hospitalar de Leiria, Leiria, Portugal	Portugal		
Tânia	Sequeira			Hospital Curry Cabral - Intensive Care Unit - UCIP7, Lisbon, Portugal	Portugal		
Ary	Serpa Neto			Monash University, Melbourne, Australia	Australia		
Pablo	Serrano Balazote			Hospital 12 de Octubre, Madrid, Spain	Spain		
Ellen	Shadowitz			Sunnybrook Health Sciences Centre, Toronto, Canada	Canada		
Syamin Asyraf	Shahidan			Kuala Lumpur Hospital, WPKL, Malaysia	Malaysia		
Anuraj	Shankar			Murni Teguh Memorial Hospital and Bunda Thamrin Hospital, North Sumatera, Indonesia	Indonesia		
Shaikh	Sharjeel			Wexford General Hospital, Wexford, Ireland	Ireland		
Pratima	Sharma			University of Michigan Schools of Medicine & Public Health, Michigan, USA	United States of America		
Catherine A.	Shaw			Oxford University (ISARIC4C), Oxford, United Kingdom	United Kingdom		
Victoria	Shaw			Oxford University (ISARIC4C), Oxford, United Kingdom	United Kingdom		
John Robert	Sheenan			Galway University Hospital, Galway, Ireland	Ireland		

*First Name and Middle Initial(s)	*Last Name	*Suffix (eg, Jr, III)	Academic Degrees	Institution	Location (city, state/province, country)	Role or Contribution, eg, chair, principal investigator	Group (if more than 1 Group listed in the byline) and/or Subgroup (eg, Steering Committee)
Rohan	Shetty			Long COVID India - Terna Specialty Hospital and Research Centre, Mumbai, India	India		
Dr. Rajesh Mohan	Shetty			Manipal Hospital Whitefield, Bangalore, India	India		
Mohiuddin	Shiekh			CCA Network	Unknown		
Nobuaki	Shime			Hiroshima University, Hiroshima, Japan	Japan		
Hiroaki	Shimizu			Hyogo Prefectural Kakogawa Medical Center, Hyogo, Japan	Japan		
Keiki	Shimizu			Tokyo Metropolitan Tama Medical Center, Tokyo, Japan	Japan		
Sally	Shrapnel			University of Queensland, Brisbane, Australia	Australia		
Hoi Ping	Shum			Pamela Youde Nethersole Eastern Hospital, Chai Wan, China	China		
Nassima	Si Mohammed			INSERM, Paris, France	France		
Ng Yong	Siang			Permai Hospital, Johor, Malaysia	Malaysia		
Jeanne	Sibiude			Hôpital Louis-Mourier, Colombes, France	France		
Bountoy	Sibounheuang			Salavan Provincial Hospital, Salavan, Laos	Laos		
Atif	Siddiqui			Grand River Hospital, Kitchener, Canada	Canada		
Louise	Sigfrid			ISARIC, Pandemic Sciences Institute, University of Oxford, UK	United Kingdom		
Fatoumata	Sillah			Clinical Services Department Fajara, Gambia	Gambia		
Piret	Sillaots			North Estonia Medical Centre, Tallin, Estonia	Estonia		
Catarina	Silva			Centro Hospital e Universitário de Coimbra, Coimbra, Portugal	Portugal		
Rogério	Silva			Unidade Local de Saúde de Alto Minho, Viana Do Castelo, Portugal	Portugal		
Maria Joao	Silva			Centro Hospitalar Universitário do Porto (CHUP), Porto, Portugal	Portugal		
Benedict	Sim Lim Heng			Hospital Sungai Buloh, Ministry of Health, Selangor, Malaysia	Malaysia		
Wai Ching	Sin			Queen Mary Hospital, Pok Fu Lam, China	China		
Dario	Sinatti			Università Cattolica del Sacro Cuore, Rome, Italy	Italy		

*First Name and Middle Initial(s)	*Last Name	*Suffix (eg, Jr, III)	Academic Degrees	Institution	Location (city, state/province, country)	Role or Contribution, eg, chair, principal investigator	Group (if more than 1 Group listed in the byline) and/or Subgroup (eg, Steering Committee)
Punam	Singh			University Hospital - Waterford, Waterford, Ireland	Ireland		
Mahendra	Singh			Department of Community and Family Medicine, AIIMS, Rishikesh, India	India		
Pompini Agustina	Sitompul			RSPI Prof Dr Sulianti Saroso, Jakarta, Indonesia	Indonesia		
Karisha	Sivam			Sungai Buloh Hospital, Selangor, Malaysia	Malaysia		
Vegard	Skogen			University Hospital of North Norway, Tromso, Norway	Norway		
Sue	Smith			ISARIC Global Support Centre, Oxford, United Kingdom	United Kingdom		
Benjamin	Smood			Perelman School of Medicine at the University of Pennsylvania, Philadelphia, United States of America	United States of America		
Coilin	Smyth			University Hospital - Waterford, Waterford, Ireland	Ireland		
Morgane	Snacken			CUB-Hopital Erasme, Anderlecht, Belgium	Belgium		
Dominic	So			Princess Margaret Hospital, Kwai Hung, China	China		
Tze Vee	Soh			Tengku Ampuan Afzan Hospital, Pahang, Malaysia	Malaysia		
Lene Bergendal	Solberg			The Norwegian Corona Cohort, Oslo, Norway	Norway		
Joshua	Solomon			McGill University Health Centre, Montreal, Canada	Canada		
Tom	Solomon			NIHR Health Protection Research Unit, Institute of Infection, Veterinary and Ecological Sciences, Faculty of Health and Life Sciences, University of Liverpool, Liverpool, UK	United Kingdom		
Emily	Somers			University of Michigan Schools of Medicine & Public Health, Michigan, USA	United States of America		
Agnès	Sommet			Centre Hospitalier Universitaire Toulouse (Larrey), Toulouse, France	France		

*First Name and Middle Initial(s)	*Last Name	*Suffix (eg, Jr, III)	Academic Degrees	Institution	Location (city, state/province, country)	Role or Contribution, eg, chair, principal investigator	Group (if more than 1 Group listed in the byline) and/or Subgroup (eg, Steering Committee)
Rima	Song			Middlemore Hospital (Canties Manukan Health), Otahuhu, New Zealand	New Zealand		
Myung Jin	Song			Seoul National University Bundang Hospital, Seoul, South Korea	South Korea		
Tae	Song			University of Chicago, Chicago, United States of America	United States of America		
Jack	Song Chia			Galway University Hospital, Galway, Ireland	Ireland		
Arne	Søraas			The Norwegian Corona Cohort, Oslo, Norway	Norway		
Albert	Sotto			Centre Hospitalier Universitaire de Nîmes, Nîmes, France	France		
Edouard	Soum			Centre Hospitalier de Périgueux, Périgueux, France	France		
Marta	Sousa			Hospital Santa Maria, Centro Hospitalar Universitário Lisboa Norte, Amadora, Portugal	Portugal		
Ana Chora	Sousa			Hospital Espírito Santo de Évora, Évora, Portugal	Portugal		
Maria	Sousa Uva			Hospital Santa Maria, Centro Hospitalar Universitário Lisboa Norte, Amadora, Portugal	Portugal		
Vicente	Souza-Dantas			Hospital Universitário Clementino Fraga Filho, Rio de Janeiro, Brazil	Brazil		
Alexandra	Sperry			Perelman School of Medicine at the University of Pennsylvania, Philadelphia, United States of America	United States of America		
Elisabetta	Spinuzza			University Hospital Policlinico Paolo Giaccone, Palermo, Italy	Italy		
B. P. Sanka Ruwan	Sri Darshana			CCA Network	Unknown		
Shiranee	Sriskandan			Department of Infectious Disease, Imperial College London, London, UK	United Kingdom		
Sarah	Stabler			Centre Hospitalier Universitaire de Lille, Lille, France	France		
Thomas	Staudinger			Medical University of Vienna, Vienna, Austria	Austria		
Stephanie-Susanne	Stecher			LMU Hospital Munich, Medical Department II, Campus Großhadern, Munich, Germany	Germany		

*First Name and Middle Initial(s)	*Last Name	*Suffix (eg, Jr, III)	Academic Degrees	Institution	Location (city, state/province, country)	Role or Contribution, eg, chair, principal investigator	Group (if more than 1 Group listed in the byline) and/or Subgroup (eg, Steering Committee)
Trude	Steinsvik			Baerum Sykehus, Gjettum, Norway	Norway		
Ymkje	Stienstra			University Medical Center Groningen, Groningen, Netherlands	Netherlands		
Birgitte	Stiksrud			Oslo University Hospital, Oslo, Norway	Norway		
Eva	Stolz			Beaumont Hospital, Dublin, Ireland	Ireland		
Amy	Stone			Cork University Hospital, Cork, Ireland	Ireland		
Adrian	Streinu-Cercel			National Institute for Infectious Diseases Matei Bals, Bucharest, Romania	Romania		
Anca	Streinu-Cercel			Carol Davila University of Medicine and Pharmacy, Bucharest, Romania	Romania		
Ami	Stuart			University of Utah, Salt Lake City, United States of America	United States of America		
David	Stuart			Division of Structural Biology, The Wellcome Centre for Human Genetics, University of Oxford, Headington, Oxford, OX3 7BN, UK	United Kingdom		
Richa	Su			Hôpital Bichat Claude-Bernard AP-HP, Paris, France	France		
Decy	Subekti			Murni Teguh Memorial Hospital and Bunda Thamrin Hospital, North Sumatera, Indonesia	Indonesia		
Gabriel	Suen			Sturgeon Community Hospital, St Albert, Canada	Canada		
Jacky Y.	Suen			University of Queensland, Brisbane, Australia	Australia		
Prasanth	Sukumar			St Vincents University Hospital, Dublin, Ireland	Ireland		
Asfia	Sultana			St Joseph's Health Center, Sherbrooke, Canada	Canada		
Charlotte	Summers			Department of Medicine, University of Cambridge, Cambridge, Cambridgeshire, UK	United Kingdom		
Dubravka	Supic			Connolly Hospital Blanchardstown, Dublin, Ireland	Ireland		
Deepashankari	Suppiah			Sarawak General Hospital, Sarawak, Malaysia	Malaysia		
Magdalena	Surovcová			University Hospital Ostrava, Ostrava-Poruba, Czechia	Czechia		

*First Name and Middle Initial(s)	*Last Name	*Suffix (eg, Jr, III)	Academic Degrees	Institution	Location (city, state/province, country)	Role or Contribution, eg, chair, principal investigator	Group (if more than 1 Group listed in the byline) and/or Subgroup (eg, Steering Committee)
Atie	Suwarti			Murni Teguh Memorial Hospital and Bunda Thamrin Hospital, North Sumatera, Indonesia	Indonesia		
Andrey	Svistunov			Sechenov University, Moscow, Russia	Russia		
Sarah	Syahrin			Sungai Buloh Hospital, Selangor, Malaysia	Malaysia		
Konstantinos	Syrigos			Sotiria General Hospital, Athens, Greece	Greece		
Jaques	Sztajnbok			Instituto de Infectologia Emílio Ribas, Sao Paulo, Brazil	Brazil		
Konstanty	Szuldrzynski			University Hospital in Krakow, Krakow, Poland	Poland		
Shirin	Tabrizi			McGill University Health Centre, Montreal, Canada	Canada		
Fabio S.	Taccone			CUB-Hôpital Erasme, Bruxelles, Belgium	Belgium		
Lysa	Tagherset			INSERM, Paris, France	France		
Shahdattul Mawarni	Taib			Kuala Lumpur Hospital, WPKL, Malaysia	Malaysia		
Ewa	Talarek			Department of Children's Infectious Diseases, Warsaw, Poland	Poland		
Sara	Taleb			Hamad General Hospital, Doha, Qatar	Qatar		
Jelmer	Talsma			Gelre Hospitals, Zutphen, Netherlands	Netherlands		
Renaud	Tamisier			Centre Hospitalier Universitaire Grenoble-Alpes_FU, Grenoble, France	France		
Maria Lawrensia	Tampubolon			RSPI Prof Dr Sulianti Saroso, Jakarta, Indonesia	Indonesia		
Kim Keat	Tan			Sungai Buloh Hospital, Selangor, Malaysia	Malaysia		
Yan Chyi	Tan			Sungai Buloh Hospital, Selangor, Malaysia	Malaysia		
Hiroyuki	Tanaka			Kyoto Medical Centre, Kyoto, Japan	Japan		
Taku	Tanaka			Nagoya University Hospital, Nagoya, Japan	Japan		
Hayato	Taniguchi			Yokohama City University Medical Center, Yokohama, Japan	Japan		
Coralie	Tardivon			INSERM, Paris, France	France		
Pierre	Tattevin			Centre Hospitalier Universitaire Rennes (Hôpital Pontchaillou), Rennes, France	France		
M Azhari	Taufik			Fatmawati Hospital, Jakarta, Indonesia	Indonesia		
Hassan	Tawfik			Galway University Hospital, Galway, Ireland	Ireland		

*First Name and Middle Initial(s)	*Last Name	*Suffix (eg, Jr, III)	Academic Degrees	Institution	Location (city, state/province, country)	Role or Contribution, eg, chair, principal investigator	Group (if more than 1 Group listed in the byline) and/or Subgroup (eg, Steering Committee)
Tze Yuan	Tee			Tawau Hospital, Sabah, Malaysia	Malaysia		
João	Teixeira			Hospital Curry Cabral - Intensive Care Unit - UCIP7, Lisbon, Portugal	Portugal		
Sofia	Tejada			Hospital Universitario de Alava, Araba, Spain	Spain		
Marie-Capucine	Tellier			INSERM, Paris, France	France		
Sze Kye	Teoh			Raja Perempuan Zainab II Hospital, Kelantan, Malaysia	Malaysia		
Vanessa	Teotonio			Complexo Hospitalar Dr. Clementino Fraga, João Pessoa city, Brazil	Brazil		
François	Téoulé			INSERM, Paris, France	France		
Olivier	Terrier			INSERM, Paris, France	France		
Nicolas	Terzi			Centre Hospitalier Universitaire Grenoble-Alpes, Grenoble, France	France		
Hubert	Tessier-Grenier			Centre hospitalier de l'université de Montréal, Montreal, Canada	Canada		
Adrian	Tey			University Hospital - Limerick, Limerick, Ireland	Ireland		
Alif Adlan Mohd	Thabit			Sungai Buloh Hospital, Selangor, Malaysia	Malaysia		
Zhang Duan	Tham			Kuala Lumpur Hospital, WPKL, Malaysia	Malaysia		
Suvintheran	Thangavelu			Sungai Buloh Hospital, Selangor, Malaysia	Malaysia		
Elmi	Theron			Galway University Hospital, Galway, Ireland	Ireland		
Vincent	Thibault			Centre Hospitalier Universitaire Rennes (Hôpital Pontchaillou), Rennes, France	France		
Simon-Djamel	Thiberville			Centre Hospitalier Louis Raffalli, Manosque, France	France		
Benoît	Thill			Centre Hospitalier de Béziers, Béziers, France	France		
Jananee	Thirumanickam			Sungai Buloh Hospital, Selangor, Malaysia	Malaysia		
Shaun	Thompson			University of Nebraska Medical Center, Omaha, United States of America	United States of America		
Niamh	Thompson			Clinical Services Department Fajara, Gambia	Gambia		
David	Thomson			Division of Critical Care, University of Cape Town and Groote Schuur Hospital, Cape Town, South Africa	South Africa		

*First Name and Middle Initial(s)	*Last Name	*Suffix (eg, Jr, III)	Academic Degrees	Institution	Location (city, state/province, country)	Role or Contribution, eg, chair, principal investigator	Group (if more than 1 Group listed in the byline) and/or Subgroup (eg, Steering Committee)
Emma C.	Thomson			MRC-University of Glasgow Centre for Virus Research, 464 Bearsden Road, Glasgow, UK	United Kingdom		
Mathew	Thorpe			Oxford University (ISARIC4C), Oxford, United Kingdom	United Kingdom		
Surain Raaj Thanga	Thurai			Sungai Buloh Hospital, Selangor, Malaysia	Malaysia		
Ryan S.	Thwaites			Oxford University (ISARIC4C), Oxford, United Kingdom	United Kingdom		
Paul	Tierney			Tallaght University Hospital, Dublin, Ireland	Ireland		
Vadim	Tieroshyn			Lugansk State Medical University - Department of Internal Medicine No2, Lugansk, Ukraine	Ukraine		
Peter S	Timashev			Sechenov University, Moscow, Russia	Russia		
Jean-François	Timsit			INSERM, Paris, France	France		
Noémie	Tissot			Centre Hospitalier Universitaire de Besançon, Besançon, France	France		
Fiona	Toal			St Vincents University Hospital, Dublin, Ireland	Ireland		
Jordan Zhien Yang	Toh			Kuala Lumpur Hospital, WPKL, Malaysia	Malaysia		
Maria	Toki			Sotiria General Hospital, Athens, Greece	Greece		
Kristian	Tonby			Oslo University Hospital, Oslo, Norway	Norway		
Sia Loong	Tonnii			Sarawak General Hospital, Sarawak, Malaysia	Malaysia		
Marta	Torre			Hospital Curry Cabral - Intensive Care Unit - UCIP7, Lisbon, Portugal	Portugal		
Margarida	Torres			Hospital de Curry Cabral - Infectious Diseases, Lisbon, Portugal	Portugal		
Antoni	Torres			Hospital Clinic, Barcelona, Spain	Spain		
Rosario Maria	Torres Santos-Olmo			La Paz Hospital, Madrid, Spain	Spain		
Hernando	Torres-Zevallos			Clínica Internacional, Lima, Peru	Peru		
Michael	Towers			Beaumont Hospital, Dublin, Ireland	Ireland		
Tony	Trapani			Albury Wodonga Health, Albury, Australia	Australia		

*First Name and Middle Initial(s)	*Last Name	*Suffix (eg, Jr, III)	Academic Degrees	Institution	Location (city, state/province, country)	Role or Contribution, eg, chair, principal investigator	Group (if more than 1 Group listed in the byline) and/or Subgroup (eg, Steering Committee)
Cécile	Tromeur			Centre Hospitalier Universitaire de Brest, Brest, France	France		
Ioannis	Trontzas			Sotiria General Hospital, Athens, Greece	Greece		
Tiffany	Trouillon			Centre Hospitalier Universitaire de Saint-Étienne, Saint-Étienne, France	France		
Jeanne	Truong			Hôpital Robert-Debré AP-HP, Paris, France	France		
Christelle	Tual			INSERM, Paris, France	France		
Sarah	Tubiana			INSERM, Paris, France	France		
Helen	Tuite			Galway University Hospital, Galway, Ireland	Ireland		
Alexis F.	Turgeon			Hôpital de l'Enfant-Jésus, Quebec, Canada	Canada		
Jean-Marie	Turmel			Centre Hospitalier Universitaire de Martinique, Fort-de-France, Saint Martin (French)	Saint Martin (French)		
Lance C.W.	Turtle			Oxford University (ISARIC4C), Oxford, United Kingdom	United Kingdom		
Anders	Tveita			Baerum Sykehus, Gjettum, Norway	Norway		
Pawel	Twardowski			Dunedin Public Hospital, Dunedin, New Zealand	New Zealand		
Makoto	Uchiyama			Legacy Emanuel Medical Center, Portland, United States of America	United States of America		
PG Ishara	Udayanga			CCA Network	Unknown		
Andrew	Udy			Monash University, Melbourne, Australia	Australia		
Roman	Ullrich			Medical University of Vienna, Vienna, Austria	Austria		
Alberto	Uribe			Ohio State University, Columbus, United States of America	United States of America		
Asad	Usman			Perelman School of Medicine at the University of Pennsylvania, Philadelphia, United States of America	United States of America		
Timothy M.	Uyeki			WHO-ISARIC Clinical Characterisation Protocol & SPRINT-SARI, United Kingdom	United Kingdom		
Michel	Vaillant			Competence Centre for Methodology and Statistics, Luxembourg Institute of Health, Strassen, Luxembourg	Luxembourg		

*First Name and Middle Initial(s)	*Last Name	*Suffix (eg, Jr, III)	Academic Degrees	Institution	Location (city, state/province, country)	Role or Contribution, eg, chair, principal investigator	Group (if more than 1 Group listed in the byline) and/or Subgroup (eg, Steering Committee)
Cristinava	Vajdovics			University Hospital, Kerry, Ireland	Ireland		
Piero	Valentini			Università Cattolica del Sacro Cuore, Rome, Italy	Italy		
Luís	Val-Flores			Hospital Curry Cabral - Intensive Care Unit - UCIP7, Lisbon, Portugal	Portugal		
Stijn	Van de Velde			AZ Maria Middelares, Gent, Belgium	Belgium		
Marcel	van den Berge			ADRZ, Amsterdam, Netherlands	Netherlands		
Machteld	van der Feltz			Alrijne Hospital, Leiden, Netherlands	Netherlands		
Job	van der Palen			Medisch Spectrum Twente, Zutphen, Netherlands	Netherlands		
Paul	van der Valk			Medisch Spectrum Twente, Zutphen, Netherlands	Netherlands		
Nicky	Van Der Vekens			AZ Maria Middelares, Gent, Belgium	Belgium		
Peter	Van der Voort			University Medical Center Groningen, Groningen, Netherlands	Netherlands		
Sylvie	Van Der Werf			INSERM, Paris, France	France		
Laura	van Gulik			Meander Medical Centre, Amersfoort, Netherlands	Netherlands		
Jarne	Van Hattem			Tergooi Hospital, Hilversum, Netherlands	Netherlands		
Carolien	van Netten			Adrz, Goes, Netherlands	Netherlands		
Frank	van Someren Gréve			PREPARE and RECOVER EU Consortium, Belgium	Belgium		
Ilonka	van Veen			Medisch Spectrum Twente, Zutphen, Netherlands	Netherlands		
Hugo	Van Willigen			PREPARE and RECOVER EU Consortium, Belgium	Belgium		
Noémie	Vanel			INSERM, Paris, France	France		
Henk	Vanoverschelde			AZ Maria Middelares, Gent, Belgium	Belgium		
Michael	Varrone			Northwell Health, New York, United States of America	United States of America		
Shoban Raj	Vasudayan			Tuanku Ja'afar, Negeri Sembilan, Malaysia	Malaysia		

*First Name and Middle Initial(s)	*Last Name	*Suffix (eg, Jr, III)	Academic Degrees	Institution	Location (city, state/province, country)	Role or Contribution, eg, chair, principal investigator	Group (if more than 1 Group listed in the byline) and/or Subgroup (eg, Steering Committee)
Charline	Vauchy			Centre Hospitalier Universitaire de Besançon, Besançon, France	France		
Pavan Kumar	Vecham			Apollo Hospitals Chennai, Chennai, India	India		
Shaminee	Veeran			Sungai Buloh Hospital, Selangor, Malaysia	Malaysia		
Aurélie	Veislinger			INSERM, Paris, France	France		
Sebastian	Vencken			St Vincents University Hospital, Dublin, Ireland	Ireland		
Sara	Ventura			Hospital de São José -U.U.M., Lisbon, Portugal	Portugal		
Annelies	Verbon			Erasmus Medical Centre, Rotterdam, Netherlands	Netherlands		
José Ernesto	Vidal			Instituto de Infectologia Emílio Ribas, Sao Paulo, Brazil	Brazil		
César	Vieira			Hospital Curry Cabral - Intensive Care Unit - UCIP7, Lisbon, Portugal	Portugal		
Judit	Villar			Hospital del Mar, Barcelona, Spain	Spain		
Pierre-Marc	Villeneuve			Grey Nun's Community Hospital, Edmonton, Canada	Canada		
Andrea	Villoldo			Mar del Plata Medical Foundation Private Community Hospital, Mar Del Plata, Argentina	Argentina		
Gayatri	Vishwanathan			Long COVID India - Terna Specialty Hospital and Research Centre, Mumbai, India	India		
Benoit	Visseaux			INSERM, Paris, France	France		
Hannah	Visser			Beatrix ziekenhuis, Gorinchem, Netherlands	Netherlands		
Chiara	Vitiello			Istituto Mediterraneo per i Trapianti e Terapie ad Alta Specializzazione, Palermo, Italy	Italy		
Manivanh	Vongsouvath			Salavan Provincial Hospital, Salavan, Laos	Laos		
Harald	Vonkeman			Medisch Spectrum Twente, Zutphen, Netherlands	Netherlands		
Fanny	Vuotto			Centre Hospitalier Universitaire de Lille, Lille, France	France		
Noor Hidayu	Wahab			Kuala Lumpur Hospital, WPKL, Malaysia	Malaysia		
Suhaila Abdul	Wahab			Tuanku Fauziah Hospital, Perlis, Malaysia	Malaysia		

*First Name and Middle Initial(s)	*Last Name	*Suffix (eg, Jr, III)	Academic Degrees	Institution	Location (city, state/province, country)	Role or Contribution, eg, chair, principal investigator	Group (if more than 1 Group listed in the byline) and/or Subgroup (eg, Steering Committee)
Nadirah Abdul	Wahid			Sungai Buloh Hospital, Selangor, Malaysia	Malaysia		
Marina	Wainstein			Caja Nacional De Salud, Trinidad, Bolivia	Bolivia		
Laura	Walsh			Galway University Hospital, Galway, Ireland	Ireland		
Chih-Hsien	Wang			National Taiwan University Hospital, Taipei City, Taiwan	Taiwan		
Steve	Webb			WHO-ISARIC Clinical Characterisation Protocol & SPRINT-SARI, United Kingdom	United Kingdom		
Jia	Wei			Big Data Institute, Nuffield Department of Medicine, University of Oxford, Oxford, UK	United Kingdom		
Katharina	Weil			University Hospital Dusseldorf, Dusseldorf, Germany	Germany		
Tan Pei	Wen			Tuanku Ja'afar, Negeri Sembilan, Malaysia	Malaysia		
Sanne	Wesselius			Franciscus Gasthuis & Vlietland, Rotterdam, Netherlands	Netherlands		
T. Eoin	West			University of Washington Medical Center - Northwest, Seattle, United States of America	United States of America		
Murray	Wham			Oxford University (ISARIC4C), Oxford, United Kingdom	United Kingdom		
Bryan	Whelan			Sligo University Hospital (Saolta), Sligo, Ireland	Ireland		
Nicole	White			University of Queensland, Brisbane, Australia	Australia		
Paul Henri	Wicky			Hôpital Bichat Claude-Bernard AP-HP, Paris, France	France		
Aurélie	Wiedemann			INSERM, Paris, France	France		
Surya Otto	Wijaya			RSPI Prof Dr Sulianti Saroso, Jakarta, Indonesia	Indonesia		
Keith	Wille			University of Alabama at Birmingham Hospital, Birmingham, United States of America	United States of America		
Sue	Willems			Royal Columbian Hospital, Vancouver, Canada	Canada		
Bailey	Williams			University of Nebraska Medical Centre	United States of America		

*First Name and Middle Initial(s)	*Last Name	*Suffix (eg, Jr, III)	Academic Degrees	Institution	Location (city, state/province, country)	Role or Contribution, eg, chair, principal investigator	Group (if more than 1 Group listed in the byline) and/or Subgroup (eg, Steering Committee)
Virginie	Williams			Hospital du Sacre Coeur, Montreal, Canada	Canada		
Patricia J	Williams			Monash University, Melbourne, Australia	Australia		
Jessica	Wittman			Royal Columbian Hospital, Vancouver, Canada	Canada		
Calvin	Wong			Universitair Ziekenhuis, Gent, Belgium	Belgium		
Xin Ci	Wong			Digital Health Research and Innovation Unit, Institute for Clinical Research, National Institutes of Health (NIH), Ministry of Health, Selangor, Malaysia	Malaysia		
Yew Sing	Wong			Sungai Buloh Hospital, Selangor, Malaysia	Malaysia		
Teck Fung	Wong			Sarawak General Hospital, Sarawak, Malaysia	Malaysia		
Natalie	Wright			St Bernard's Hospital, Gibraltar, Gibraltar	Gibraltar		
Lim Saio	Xian			Pulau Pinang Hospital, Pulau Pinang, Malaysia	Malaysia		
Ioannis	Xynogalas			Sotiria General Hospital, Athens, Greece	Greece		
Siti Rohani Binti Mohd	Yakop			Kuala Lumpur Hospital, WPKL, Malaysia	Malaysia		
Masaki	Yamazaki			Kyoto Prefectural University of Medicine, Kyoto, Japan	Japan		
Elizabeth	Yarad			Monash University, Melbourne, Australia	Australia		
Yazdan	Yazdanpanah			INSERM, Paris, France	France		
Nicholas	Yee Liang Hing			Kuala Lumpur Hospital, WPKL, Malaysia	Malaysia		
Cécile	Yelnik			Hôpital Albert Calmette, Lille, France	France		
Chian Hui	Yeoh			Sultanah Bahiyah Hospital, Kedah, Malaysia	Malaysia		
Stephanie	Yerkovich			University of Queensland, Brisbane, Australia	Australia		
Touxiong	Yiaye			Xieng Khouang Provincial Hospital, Phonsavan, Laos	Laos		
Toshiki	Yokoyama			Kouritu Tousei Hospital, Seto City, Japan	Japan		
Hodane	Yonis			Hôpital Lyon Sud - HCL, Lyon, France	France		
Obada	Yousif			Wexford General Hospital, Wexford, Ireland	Ireland		
Saptadi	Yuliarto			PICU Saiful Anwar Hospital, Malang, Indonesia	Indonesia		
Akram	Zaaqoq			MedStar Washington Hospital Centre, Washington, United States of America	United States of America		

*First Name and Middle Initial(s)	*Last Name	*Suffix (eg, Jr, III)	Academic Degrees	Institution	Location (city, state/province, country)	Role or Contribution, eg, chair, principal investigator	Group (if more than 1 Group listed in the byline) and/or Subgroup (eg, Steering Committee)
Marion	Zabbe			Centre Hospitalier de Périgueux, Périgueux, France	France		
Kai	Zacharowski			Uniklinik University Hospital, Frankfurt, Germany	Germany		
Masliza	Zahid			Sultanah Aminah Hospital, Johor, Malaysia	Malaysia		
Maram	Zahran			Rambam Hospital, Haifa, Israel	Israel		
Nor Zaila Binti	Zaidan			Melaka Hospital, Melaka, Malaysia	Malaysia		
Maria	Zambon			Oxford University (ISARIC4C), Oxford, United Kingdom	United Kingdom		
Miguel	Zambrano			Instituto Nacional Del Tórax, Santiago, Chile	Chile		
Alberto	Zanella			Fondazione IRCCS Ca, Milan, Italy	Italy		
Konrad	Zawadka			Department of Children's Infectious Diseases, Warsaw, Poland	Poland		
Nurul	Zaynah			Kuala Lumpur Hospital, WPKL, Malaysia	Malaysia		
Hiba	Zayyad			The Baruch Padeh Medical Center Poriya, Tiberias, Israel	Israel		
Alexander	Zoufaly			Sozialmedizinisches Zentrum Sud, Vienna, Austria	Austria		
David	Zucman			Hôpital Foch, Suresnes, France	France		
