# Supplementary material for: Implementation of Recommendations on the Use of Corticosteroids in Severe COVID-19
Source: JAMA Netw Open. 2023 Dec 26;6(12):e2346502. doi: 10.1001/jamanetworkopen.2023.46502 (PMC10751594; doi:10.1001/jamanetworkopen.2023.46502)
Supplement: Supplement 3. — Data Sharing Statement [file jamanetwopen-e2346502-s003.pdf]

Data Sharing Statement

Camirand-Lemyre. Implementation of Recommendations on the Use of Corticosteroids in Severe COVID-19. *JAMA Netw Open*. Published December 26, 2023.
doi:10.1001/jamanetworkopen.2023.46502

Data
Data available: Yes
Data types: Deidentified participant data, Data dictionary
How to access data: The data that underpin this analysis are highly detailed clinical data on individuals hospitalised with COVID-19. Due to the sensitive nature of these data and the associated privacy concerns, they are available via a governed data access mechanism following review of a data access committee. Data can be requested via the IDDO COVID-19 Data Sharing Platform (http://www.iddo.org/covid-19). The Data Access Application, Terms of Access and details of the Data Access Committee are available on the website. Briefly, the requirements for access are a request from a qualified researcher working with a legal entity who have a health and/or research remit; a scientifically valid reason for data access which adheres to appropriate ethical principles. The full terms are at: https://www.iddo.org/document/covid-19-data-access-guidelines. A small subset of sites who contributed data to this analysis have not agreed to pooled data sharing as above. In the case of requiring access to these data, please contact the corresponding author in the first instance who will look to facilitate access.
When available: With publication

Supporting Documents
Document types: Statistical/analytic code
How to access documents: After data-sharing agreement is approved by ISARIC, the analytic code will be made available by Pr. Félix Camirand-Lemyre (Felix.Camirand.Lemyre@USherbrooke.ca)
When available: With publication

Additional Information
Who can access the data: After data-sharing agreement is approved by ISARIC
Types of analyses: For any purpose approved by ISARIC
Mechanisms of data availability: Following fulfilment of ISARIC conditions and requirements (https://isaric.org/partner-analyses-guidance/)
